# Heme Oxygenase 1/Peroxisome Proliferator-Activated Receptor Gamma Pathway Protects Intimal Hyperplasia and Mitigates Arteriovenous Fistula Dysfunction by Regulating Oxidative Stress and Inflammatory Response

**DOI:** 10.1155/2022/7576388

**Published:** 2022-06-12

**Authors:** Tingfei Xie, Yunpeng Xu, Lecai Ji, Xiaolu Sui, Aisha Zhang, Yanzi Zhang, Jihong Chen

**Affiliations:** ^1^Department of Nephrology, Affiliated Bao'an Hospital of Shenzhen, The Second School of Clinical Medicine, Southern Medical University, Shenzhen, Guangdong 518000, China; ^2^Department of Nephrology, Bao'an Clinical Medical School of Guangdong Medical University, Shenzhen, Guangdong 518000, China; ^3^The Second School of Clinical Medicine, Southern Medical University, Shenzhen, Guangdong 518000, China; ^4^Department of Tuberculosis, Shenzhen Center for Chronic Disease Control, Shenzhen, Guangdong 518000, China; ^5^Department of Nephrology, Affiliated Bao'an Hospital of Shenzhen, Southern Medical University, Shenzhen, Guangdong 518000, China

## Abstract

**Purpose:**

An arteriovenous fistula (AVF) is the preferred vascular access mode for maintenance hemodialysis, and access stenosis and thrombosis are the primary causes of AVF dysfunction. This study is aimed at exploring the molecular mechanisms underlying AVF development and the roles of the heme oxygenase 1/peroxisome proliferator-activated receptor gamma (HO-1/PPAR-*γ*) pathway in AVF.

**Method:**

AVF model mice were established, and the vascular tissues from the arteriovenous anastomosis site were sent for mRNA sequencing. Differentially expressed mRNAs (DEmRNAs) were screened and subjected to functional analysis. Thereafter, the mice with HO-1 knockdown and coprotoporphyrin IX chloride (COPP) pretreatment were used to investigate the roles of the HO-1/PPAR-*γ* pathway in AVF.

**Results:**

By sequencing, 2514 DEmRNAs, including 1323 upregulated and 1191 downregulated genes, were identified. These DEmRNAs were significantly enriched in the PPAR signaling pathway, AMPK signaling pathway, glucagon signaling pathway, IL-17 signaling pathway, and Toll-like receptor signaling pathway. High expression of HO-1 and PPAR-*γ* reduced endothelial damage and intimal hyperplasia during AVF maturation. After AVF was established, the levels of transforming growth factor-*β* (TGF-*β*), interleukin-1*β* (IL-1*β*), interleukin-18 (IL-18), and reactive oxygen species (ROS) were significantly increased (*P* < 0.05), and *HO-1* normal expression and COPP pretreatment evidently decreased their levels in AVF (*P* < 0.05). Additionally, AVF significantly upregulated HO-1 and PPAR-*γ* and downregulated MMP9, and COPP pretreatment and HO-1 normal expression further upregulated and downregulated their expression.

**Conclusion:**

The HO-1/PPAR-*γ* pathway may suppress intimal hyperplasia induced by AVF and protect the intima of blood vessels by regulating MMP9 and ROS, thus mitigating AVF dysfunction.

## 1. Introduction

Arteriovenous fistula (AVF) dysfunction is an important independent risk factor for death in maintenance hemodialysis patients, and access stenosis and thrombosis are the primary complications leading to AVF dysfunction [1, 2]. Autologous AVF is the preferred vascular access mode for maintenance hemodialysis [3]. The one-year patency of AVF is approximately 70%, the two-year patency is 65%, and the four-year patency is 48% [4]. Among them, the number of hospitalized patients due to vascular access stenosis and embolization accounted for 15%-24% of the total number of patients hospitalized for dialysis treatment [5]. Therefore, to prolong the life of maintenance hemodialysis patients, there is an urgent need to develop novel therapeutic targets and pathways to control AVF dysfunction.

AVF maturation is complex and requires cell migration and proliferation as well as extracellular matrix deposition and remodeling to achieve outward remodeling and wall thickening [6]. The main reasons for AVF dysfunction are as follows: surgical injury and hemodynamic changes during autologous AVF establishment can contribute to the damage of endothelial cells and endometrial smooth muscle cells. Endothelial injury can lead to a series of cascading events, such as oxidative stress and inflammatory response, thereby leading to progressive abnormal persistent hyperplasia of the intima and eventually to the loss of arteriovenous stenosis [7]. Therefore, the regulation of oxidative stress and the inflammatory response may be important breakthrough targets for inhibiting intimal hyperplasia and protecting the vascular endothelium [8].

Heme oxygenase-1 (HO-1), which is widely expressed in human and mammalian tissues, is an initiator and rate-limiting enzyme that catalyzes heme and is a microsomal oxidase with multiple functions [9]. Previous studies have reported that HO-1 overexpression and the production of bilirubin and carbonic oxide (CO) can suppress the abnormal proliferation of vascular smooth muscle cells by inhibiting oxidative stress and the inflammatory response, thus playing essential roles in anti-inflammation, antioxidation, and cell homeostasis maintenance [9–11]. Durante and Lin [12] showed that HO-1 is induced in AVFs and is the key determinant of AVF survival. Another study found that HO-1 knockout enhanced neointimal hyperplasia, thickened the venous wall of the fistulas, and resulted in fistula blockage, which seriously impaired the function of AVF in mice [13]. However, the specific mechanisms by which HO-1 affects AVF function require further exploration.

Peroxisome proliferator-activated receptor gamma (PPAR-*γ*) has been shown to transcriptionally regulate HO-1 expression, suggesting that PPAR-*γ* can exert anti-inflammatory and antiproliferative effects via HO-1 upregulation [14]. Therefore, our study is aimed at exploring the molecular mechanisms of AVF development and the roles of HO-1/PPAR-*γ* pathway in AVF. First, an AVF model was constructed, and the molecular mechanisms of AVF development were analyzed using sequencing. Our sequencing results showed that HO-1 and PPAR-*γ* were both upregulated in AVF, which was in accordance with previous studies [12, 13]; thus, we chose HO-1/PPAR-*γ* as the objective for subsequent experiments. After that, HO-1 knockdown mice were pretreated with coprotoporphyrin IX chloride (COPP, an agonist of PPAR-*γ*) to investigate the roles of the HO-1/PPAR-*γ* pathway in AVF. These results provide valuable clues for identifying novel potential therapeutic targets and pathways for AVF.

## 2. Materials and Methods

### 2.1. Mice

SPF male C57BL/6 mice aged 6-8 weeks were purchased from Guangdong Medical Laboratory Animal Center (Guangdong, China), and male mice with HO-1 knockdown (HO-1^+/-^) aged 6-8 weeks were obtained from Jiangsu Gem Pharmatech Biological Technology Co. Ltd. (Nanjing, China). All the mice were bred under controlled temperature (24 ± 2°C) and humidity (50 ± 5%) conditions with a 12 h light/dark cycle. During the experiment, mice had free access to food and water. All animal experiments were performed in accordance with the ARRIVE and National Medical Advisory Committee (NMAC) guidelines using approved procedures of the Institutional Animal Care and Use Committee of Shenzhen Peking University Hong Kong University of Science and Technology Medical Center (approval no. BYL20200802).

### 2.2. Establishment of AVF Model

After acclimatization for 7 days, 20 C57BL/6 mice were randomly divided into two groups: the normal control (NC) and AVF groups. Mice in the AVF group were used to establish an AVF model, as described previously [15]. Briefly, the mice in the AVF group were anesthetized using 2%-4% isoflurane and placed under an anatomical microscope. Subsequently, the skin and subcutaneous tissues were cut along the midline from the thyroid cartilage to the upper part of the sternum, exposing the right common jugular vein and right common carotid artery. All branches of the jugular vein were ligated, and the proximal end of the common carotid artery was clipped. An incision approximately 1 mm in length was made, and the incision and common carotid artery were sutured with nylon sutures. Subsequently, the vascular clamp was removed, and the patency of the AVF was assessed visually using a mixture of venous and arterial blood. After the operation, the mice were subcutaneously injected with 1.5 mL of normal saline and irradiated under a heat-spot lamp until they awoke. However, arterial and venous sutures were not used in the NC group, and the remaining procedures were the same. At 21 days after successful establishment, all mice were killed by cervical dislocation, and vascular tissues from the arteriovenous anastomosis site were collected for mRNA sequencing.

### 2.3. mRNA Sequencing

The tissues obtained from the NC and AVF groups (*n* = 3 for each group) were sent to Beijing Novogene Technology Co. Ltd. (Beijing, China) for mRNA sequencing and proteome sequencing. Raw mRNA sequencing data were processed using an in-house Perl script, and clean data were obtained by removing reads containing adapters, reads containing ploy-N, and reads with low quality. Clean reads were then aligned to the reference genome of mice using Hisat2 v2.0.5. The fragments per kilobase of transcript sequence per million base pairs sequence (FPKM) of each sample were calculated, and differentially expressed mRNAs (DEmRNAs) were screened using the edgeR R package (3.18.1) with an adjusted *P* value (padj) < 0.05, and |log_2_ fold change (FC)| >1. Functional analyses of DEmRNAs, including gene ontology (GO) and Kyoto Encyclopedia of Genes and Genomes (KEGG), were performed using the clusterProfiler R package. Significant GO terms and KEGG pathways were considered with padj < 0.05.

### 2.4. Treatment of Mice

Ten HO-1^+/+^ mice and 30 HO-1^+/-^ mice were divided into four groups (*n* = 10) as follows: HO-1^+/+^+AVF, HO-1^+/-^+AVF, HO-1^+/-^+AVF+COPP, and HO-1^+/-^+sham groups. After acclimatization for 3 days, mice in the HO-1^+/-^+AVF+COPP group were intraperitoneally injected with 5 mg/kg COPP (cat. no. C1900-100MG, Sigma) for three days, and the other mice were injected with the same dose of saline. Mice in the HO-1^+/+^+AVF, HO-1^+/-^+AVF, and HO-1^+/-^+AVF+COPP groups were used to construct the AVF model as previously described. Twenty-one days after surgery, all mice were euthanized by cervical dislocation. Blood sera were collected, and vascular tissues from the arteriovenous anastomosis site and endothelial cells were collected for further experiments.

### 2.5. Detection of Biochemical Index

The obtained blood serum was used to determine the total cholesterol (TC), triglyceride (TG), high-density lipoprotein (HDL), low-density lipoprotein (LDL), lipoprotein A (APO-A), and total bilirubin (TBIL) levels with their corresponding assay kits (BIOSINO Co. Ltd., Beijing, China) following the manufacturer's instructions.

### 2.6. Histopathology and Immunohistochemical (IHC) Staining

One part of the vascular tissue from the arteriovenous anastomosis site was fixed in 4% paraformaldehyde (China National Pharmaceutical Group Corporation, Shanghai, China) for 24 h and embedded in paraffin. Then, 5 *μ*m sections were cut. Some of the sections were stained with hematoxylin-eosin (HE) for histopathological examination, and the other sections were used for IHC staining. For IHC staining [16], the sections were dewaxed, rehydrated, and subjected to antigen retrieval. After blocking endogenous peroxidase activity, the samples were, respectively, incubated with anti-CD31 antibody (1 : 200, Wuhan Servicebio Co. Ltd, Wuhan, China) at 4°C overnight. After washing three times with PBST, the samples were incubated with horseradish enzyme-labeled secondary antibody (1 : 200, cat. no. G1215-3, Servicebio) at 37°C for 30 min. After washing, the sections were stained with diaminobenzidine (DAB) for 1 min and stained with hematoxylin for 30 s. After dehydration and sealing, the slides were scanned and images were taken under an optical microscope (Olympus Corporation, Tokyo, Japan) at 400x magnification.

### 2.7. Detection of Reactive Oxygen Species (ROS) in Vascular Tissues

Vascular tissues were collected from the mice of the different groups, and the content of ROS in vascular tissues was examined using an ROS kit (HY-M0087, Beijing Huaying Institute of Biotechnology, Beijing, China) according to the manufacturer's protocols.

### 2.8. Enzyme-Linked Immunosorbent Assay (ELISA)

The levels of transforming growth factor-*β* (TGF-*β*), interleukin-1*β* (IL-1*β*), and interleukin-18 (IL-18) in the blood serum of different groups were determined using a mouse TGF-*β* ELISA assay kit (Shanghai mlbio Co. Ltd, Shanghai, China), a mouse IL-1*β* ELISA assay kit (Shanghai mlbio Co. Ltd), and a mouse IL-18 ELISA assay kit (Shanghai mlbio Co. Ltd), respectively, in accordance with the manufacturer's recommendations.

### 2.9. Real-Time Quantitative PCR (RT-qPCR)

Total RNA was extracted from the vascular tissues of different groups using TRIzol reagent (Takara, Beijing, China) according to the manufacturer's instructions. The total RNA was then reverse-transcribed into cDNA using the PrimeScript™ II 1st Strand cDNA synthesis kit (Takara), in accordance with the manufacturer's instructions. The sequences of all primers are shown in Table 1, and *β*-actin served as the housekeeping gene. The RT-qPCR reaction was predegenerated at 95°C for 15 min, a total of 45 cycles at 95°C for 10 s, 60°C for 30 s, and 72°C for 30 s. The relative mRNA levels of *HO-1*, matrix metallopeptidase 9 (*MMP9*), and *PPAR-γ* were calculated using the 2^-△△Ct^ method [17].

### 2.10. Western Blot

Total protein was isolated from the vascular tissues of the different groups using RIPA lysis buffer (Beyotime Biotechnology, Shanghai, China) according to the manufacturer's instructions, and the concentration of total protein was measured using a BCA assay kit (Boster Biological Technology Co. Ltd, CA, USA). Thereafter, the protein samples were separated using 10% SDS-PAGE, transferred to PVDF membranes, and blocked with 5% skim milk. Subsequently, the membranes were incubated with anti-HO-1 antibody (1 : 1000, cat. no. 70081S, CST), anti-MMP9 antibody (1 : 1000, cat. no. gtx100458, GeneTex), anti-PPAR-*γ* (1 : 1000, cat. no. 2435T, CST), and anti-*β*-actin (1 : 2000, cat. no. gtx109639; GeneTex) at 4°C overnight. After washing three times with PBST, the membranes were incubated with a secondary antibody (1 : 10000, cat. no. gtx213110-01; GeneTex) at 37°C for 2 h. After washing with PBST three times, the protein bands were visualized using a Millipore ECL system (Beyotime Biotechnology) and quantified using Image-Pro Plus software (v. 6.0, Media Cybernetics Imaging Technologies Inc., USA).

### 2.11. Statistical Analysis

Data are reported as mean ± standard deviation (SD). GraphPad Prism 5 software (version 7.0; GraphPad Software, Inc.) was used for statistical analysis. For comparisons between two groups, Student's unpaired *t*-test with a two-tailed method was used. One-way analysis of variance (ANOVA) followed by Tukey's post hoc test was used to analyze statistically significant differences between more than two groups. Statistical significance was set at *P* < 0.05.

## 3. Results

### 3.1. Identification of DEmRNAs and Functional Analysis

**Figure 1:**
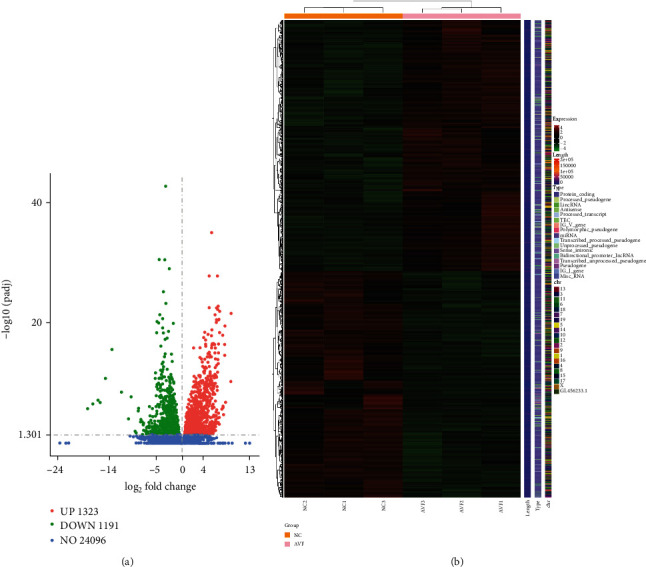
Screening of differentially expressed mRNAs (DEmRNAs) between the normal control mice and the mice with arteriovenous fistula (AVF). (a) The volcano plot of DEmRNAs. Red dots represent the upregulated genes, green dots represent the downregulated genes, and blue dots represent the genes with no significant difference. (b) Bidirectional hierarchical clustering heat map of these DEmRNAs.

**Figure 2:**
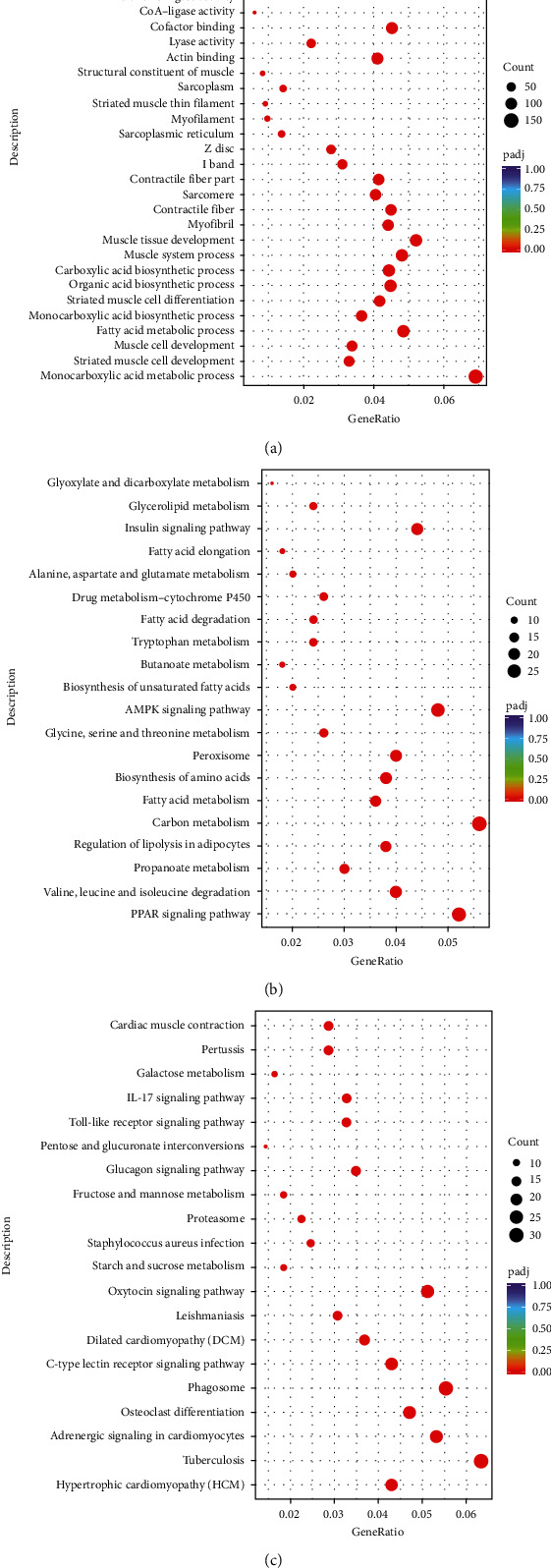
Functional analyses of these DEmRNAs. (a) The significantly enriched gene ontology (GO) terms of these DEmRNAs in molecular function, cellular component, and biological process. (b) The significantly enriched Kyoto Encyclopedia of Genes and Genomes (KEGG) of the downregulated genes. (c) The significantly enriched KEGG pathways of the upregulated genes.

Vascular tissues from mice in the NC and AVF groups were sent for mRNA sequencing to understand the molecular mechanisms of AVF progression. After sequencing and analysis, a total of 2514 DEmRNAs, including 1323 upregulated genes and 1191 downregulated genes, were identified with thresholds of padj < 0.05 and |log_2_ FC| > 1 (Figure 1(a), Supplementary table 1). The heat map of the hierarchical cluster analysis showed that these DEmRNAs could separate the NC and AVF groups, indicating that these DEmRNAs were reliable (Figure 1(b)). Subsequently, all DEGs were subjected to GO terms and KEGG analyses. Based on the criterion of padj < 0.05, 649 GO terms were significantly enriched (Supplementary table 2), and the most significantly enriched GO terms were associated with monocarboxylic acid metabolic process, muscle tissue development, fatty acid metabolic process, organic acid biosynthetic process, and striated muscle cell differentiation (Figure 2(a)). KEGG enrichment analysis for downregulated and upregulated genes was then performed. It is clear that “PPAR signaling pathway,” “insulin signaling pathway,” “AMPK signaling pathway,” “carbon metabolism,” and “valine, leucine, and isoleucine degradation” were significantly suppressed after AVF induction compared with the NC group (Figure 2(b), Supplementary table 3). Additionally, KEGG pathways of “tuberculosis,” “adrenergic signaling in cardiomyocytes,” “glucagon signaling pathway,” “galactose metabolism,” “IL-17 signaling pathway,” “phagosome,” and “Toll-like receptor signaling pathway” were significantly activated after AVF was successfully established (Figure 2(c), Supplementary table 4).

### 3.2. Verification of Sequencing Results and HO-1 Selected for Further Experiments

**Figure 3:**
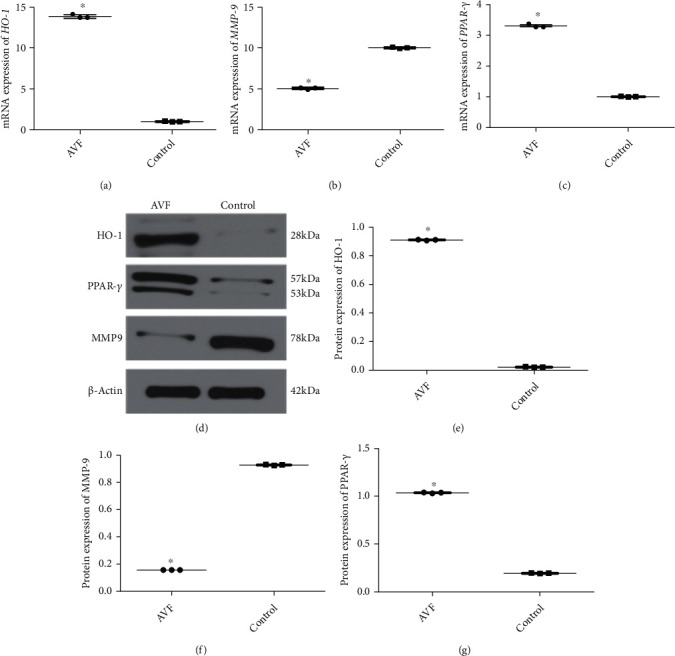
Verification of sequencing results by real-time quantitative PCR (RT-qPCR) and western blot. The mRNA expression of *HO-1* (a), *MMP9* (b), and *PPAR-γ* (c). (d) The representative protein bands visualized by western blot. The protein expression of HO-1 (e), MMP9 (f), and PPAR-*γ* (g). ^∗^*P* < 0.05, compared with the control group.

After searching the literature, we found that HO-1 and PPAR-*γ* can regulate oxidative stress reactions, and high levels of HO-1 could downregulate MMP9. Therefore, HO-1, PPAR-*γ*, and MMP9 were chosen for RT-qPCR and western blot analyses. Through sequencing, it is clear that *HO-1* and *PPAR-γ* were upregulated in the AVF group, and MMP9 was downregulated in the AVF group. RT-qPCR and western blot analyses showed that compared with the control group, the relative mRNA and protein expression levels of HO-1 and PPAR-*γ* were significantly increased in the AVF group (*P* < 0.05, Figures 3(a), 3(b), 3(e), and 3(g)). For MMP9, its mRNA and protein expression was significantly lower in the AVF group than in the control group (*P* < 0.05, Figures 3(c), 3(d), and 3(f)). These results indicated that the expression trends of HO-1, PPAR-*γ*, and MMP9 determined by RT-qPCR and western blotting were in line with those measured by mRNA sequencing, which showed a relatively high reliability of the sequencing results. Based on the sequencing results and literature search, HO-1/PPAR-*γ* was selected for subsequent experiments.

### 3.3. Upregulation of HO-1 and PPAR-*γ* Reduced the Damage and Intimal Hyperplasia Caused by AVF

**Figure 4:**
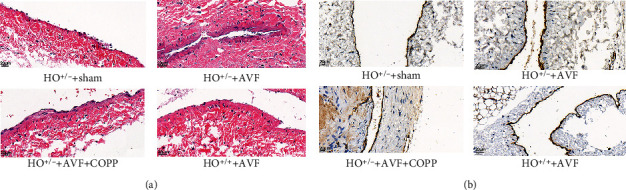
Histopathology and immunohistochemical (IHC) analyses. (a) The morphology of the vascular tissues from the arteriovenous anastomosis site stained with hematoxylin-eosin. (b) The expression of CD31 measured by IHC staining. Scale bar = 20 *μ*m.

To investigate the roles of HO-1 and PPAR-*γ* in AVFs, HO-1 knockdown mice were treated with COPP (an agonist of PPAR-*γ*). Hematoxylin and eosin (HE) staining and immunohistochemistry (IHC) staining were performed. HE staining showed that there was no damage in the endovascular layer of the HO-1^+/-^+sham group, whereas in the HO-1^+/-^+AVF+COPP and HO-1^+/+^+AVF groups, the endovascular layer was damaged and showed intimal hyperplasia; additionally, the damage and hyperplasia in the HO-1^+/-^+AVF group were more severe (Figure 4(a)). Furthermore, IHC results showed that the expression of CD31 was higher in the HO-1^+/-^+AVF group, indicating that endothelial cells were the most severely damaged, with significant intimal hyperplasia, while the expression of CD31 was relatively decreased in the HO-1^+/-^+AVF+COPP and HO-1^+/+^+AVF groups, demonstrating that intimal hyperplasia was relatively improved (Figure 4(b)). These results indicate that upregulation of HO-1 and PPAR-*γ* may be helpful in reducing damage and intimal hyperplasia caused by AVF.

### 3.4. Contents of Serum Biochemical Indexes in Different Groups

After that, the levels of TC, TG, HDL, LDL, TBIL, and APO-A in the serum of different groups were examined. The content of TC in the HO-1^+/-^+sham, HO-1^+/-^+AVF, HO-1^+/-^+AVF+COPP, and HO-1^+/+^+AVF groups was 4.05 ± 0.05 nmol/L, 3.39 ± 0.47 nmol/L, 3.93 ± 0.17 nmol/L, and 3.27 ± 0.44 nmol/L, respectively (Table 2). There was no significant difference in TG content among the four groups (*P* > 0.05). The HDL and LDL contents are shown in Table 2. For TBIL, its content in the HO-1^+/-^+AVF group (13.71 ± 0.87 *μ*mol/L) was the highest, followed by the HO-1^+/+^+AVF group (12.82 ± 0.47 *μ*mol/L), HO-1^+/-^+AVF+COPP group (12.45 ± 0.52 *μ*mol/L), and HO-1^+/-^+sham group (12.10 ± 0.17 *μ*mol/L). However, the APO-A content in these four groups was approximately 1 g/L (Table 2), which indicated that different treatments did not evidently change the APO-A content in the serum.

### 3.5. TGF-*β*, IL-1*β*, IL-18, and ROS Increased after AVF and Reduced by High Expression of HO-1 and PPAR-*γ*

**Figure 5:**
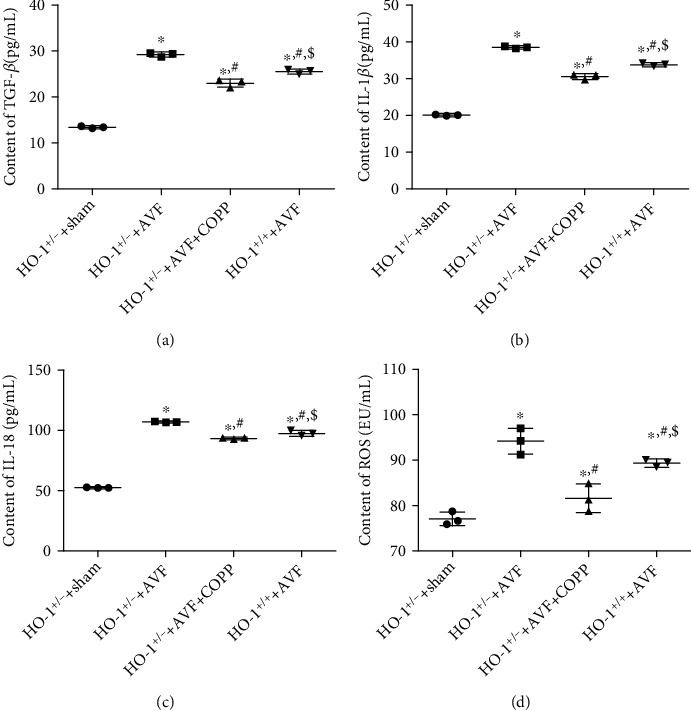
Effects of HO-1 knockdown on the levels of TGF-*β*, IL-1*β*, IL-18, and reactive oxygen species (ROS). (a) The contents of TGF-*β* in different groups. (b) The contents of IL-1*β* in different groups. (c) The contents of IL-18 in different groups. (d) The level of ROS in different groups. ^∗^*P* < 0.05, compared with the HO-1^+/-^+sham group; ^#^*P* < 0.05, compared with the HO-1^+/-^+AVF group; ^$^*P* < 0.05, compared with the HO-1^+/-^+AVF+COPP group.

To observe the inflammatory cytokines in the tissues with different treatments, corresponding ELISA assay kits were used. Compared with the HO-1^+/-^+sham group, the levels of TGF-*β* and IL-1*β* in the other three groups were significantly increased (*P* < 0.05), and their levels were significantly lower in the HO-1^+/-^+AVF+COPP and HO-1^+/+^+AVF groups than in the HO-1^+/-^+AVF group (*P* < 0.05); however, their levels were significantly higher in the HO-1^+/+^+AVF group than in the HO-1^+/-^+AVF+COPP group (*P* < 0.05, Figures 5(a) and 5(b)). IL-18 levels were markedly increased in the HO-1^+/-^+AVF group compared to those in the HO-1^+/-^+sham group (*P* < 0.05), while COPP pretreatment and HO-1 normal expression with AVF induction significantly reduced its level compared to those in the HO-1^+/-^+AVF group (*P* < 0.05), and COPP pretreatment had a better effect than that of HO-1 normal expression with AVF induction (*P* < 0.05, Figure 5(c)). Additionally, the trend of ROS content in the different groups was similar to that of the TGF-*β* and IL-1*β* levels (Figure 5(d)).

### 3.6. High Expression of HO-1 and PARP-*γ* Further Upregulated HO-1 and PPAR-*γ*, while It Downregulated MMP9

**Figure 6:**
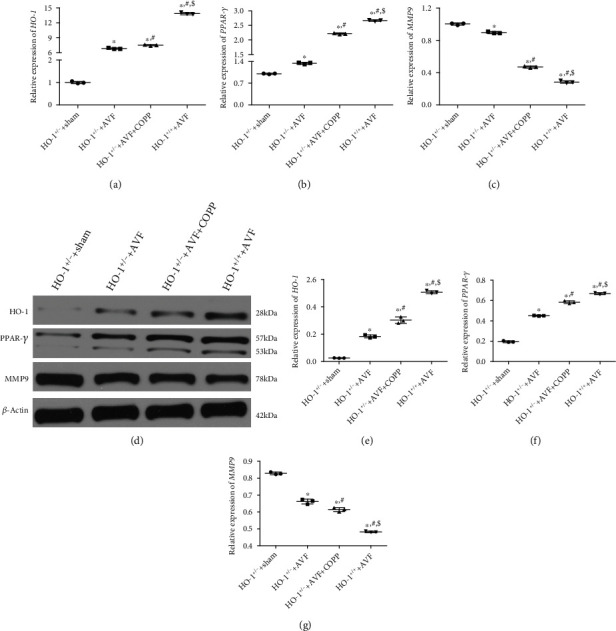
Effects of HO-1 knockdown on the expression levels of HO-1, PPAR-*γ*, and MMP9. The relative mRNA expression of *HO-1* (a), *PPAR-γ* (b), and *MMP9* (c) in different groups determined by RT-qPCR. (d) The protein bands visualized by western blot. The relative protein expression of HO-1(e), PPAR-*γ* (f), and MMP9 (g). ^∗^*P* < 0.05, compared with the HO-1^+/-^+sham group; ^#^*P* < 0.05, compared with the HO-1^+/-^+AVF group; ^$^*P* < 0.05, compared with the HO-1^+/-^+AVF+COPP group.

Finally, RT-qPCR and western blotting were employed to detect the expression levels of HO-1, PPAR-*γ*, and MMP9 in the vascular tissues after different treatments. Compared with the HO-1^+/-^+sham group, the expression of *HO-1* was significantly upregulated in the HO-1^+/-^+AVF, HO-1^+/-^+AVF+COPP, and HO-1^+/+^+AVF groups (*P* < 0.05), and COPP pretreatment and HO-1 normal expression with AVF induction evidently upregulated *HO-1* expression compared to the HO-1^+/-^+AVF group (*P* < 0.05, Figure 6(a)). The trend of *PPAR-γ* expression in the different groups was similar to that of *HO-1* expression (Figure 6(b)). MMP9 expression was significantly downregulated in the HO-1^+/-^+AVF, HO-1^+/-^+AVF+COPP, and HO-1^+/+^+AVF groups compared with the HO-1^+/-^+sham group (*P* < 0.05). COPP pretreatment and HO-1 normal expression with AVF induction further downregulated its expression (*P* < 0.05), and the downregulation was more significant in the HO-1^+/+^+AVF group (*P* < 0.05, Figure 6(c)). Additionally, the trends of HO-1, PPAR-*γ*, and MMP9 protein expression in different groups detected by western blotting were in accordance with those measured by RT-qPCR (Figures 6(d)–6(g)).

## 4. Discussion

The establishment and maturation of AVF are the best choice for hemodialysis in patients with end-stage renal disease; however, AVF dysfunction could hinder the clinical application of the fistula [18]. Therefore, it is necessary to explore the molecular mechanisms underlying AVF development and identify potential therapeutic targets. In this study, mRNA sequencing identified a total of 2514 DEmRNAs between the AVF and NC groups, including 1323 upregulated genes and 1191 downregulated genes. These DEmRNAs were significantly enriched in 649 GO terms, such as monocarboxylic acid metabolic process and muscle tissue development. KEGG results showed that after AVF was successfully established and the PPAR, insulin, and AMPK signaling pathways were suppressed, while the glucagon, IL-17, and Toll-like receptor signaling pathways were activated.

PPAR-*γ*, a ligand-activated nuclear receptor, regulates glucose and lipid metabolism, endothelial function, and inflammation [19, 20]. Liu et al. [21] found that PPAR-*γ* activation could reduce oxidative stress damage by regulating TGF-*β*1 and HGF, thus playing important roles in kidney protection. AMPK, a key mediator of energy balance, is activated when intracellular ATP production is reduced and plays a key role in regulating cell growth and reprogramming metabolism [22]. A previous study showed that tilianin pretreatment improved mitochondrial energy metabolism and decreased oxidative stress through the AMPK/SIRT1/PGC-1*α* signaling pathway, thus attenuating myocardial ischemia/reperfusion injury [23]. The insulin and glucagon signaling pathways have been reported to participate in various aspects of physiology, including glucose homeostasis, cell growth, and aging [24]. The IL-17 signaling pathway, a highly versatile proinflammatory cytokine, is critical to a variety of processes, including host defense, tissue repair, pathogenesis of inflammatory diseases, and cancer progression [25]. Cai et al. [26] found that IL-17Rb was downregulated in female AVFs. In addition, a previous study by Zhang et al. [27] found Toll-like receptor signaling pathway was associated with the osteogenic differentiation potential of bone mesenchymal stem cells through bioinformatic analysis. When these reports are combined with our results, we can speculate that the oxidative stress response (PPAR signaling pathway, insulin signaling pathway, AMPK signaling pathway, and glucagon signaling pathway) and inflammatory response (IL-17 signaling pathway and Toll-like receptor signaling pathway) may be closely associated with AVF construction and progression.

Additionally, a previous study by Sadaghianloo et al. also showed that AVF maturation was related to the oxidative stress response [28]. HO-1 has been reported to be closely associated with cellular antioxidant defenses [29, 30]; therefore, HO-1 was chosen as the subject for subsequent experiments. It was found that high expression of *HO-1* and *PPAR-γ* could reduce endothelial damage and intimal hyperplasia during AVF maturation, indicating that the function of AVF may be improved [31]. During the establishment of AVF, shear stress and hypoxia can promote inflammation and cell proliferation, which progress to the extent of stenosis and thrombosis [32, 33]. TGF-*β* signaling has been shown to play a central role in vascular remodeling of AVFs, and TGF-*β* has been proposed to be closely related to intimal hyperplasia [34, 35]. A previous study indicated that TGF-*β*-activated kinase 1 was increased in mature AVFs and regulated AVF maturation *in vivo* [34]. IL-1*β* is another major factor specific to AVF, and IL-18 is a proinflammatory cytokine. These cytokines can further trigger an activation cascade that leads to inflammation, adhesion, and ultimately, increased plaque or thrombus formation [36, 37]. Chan et al. [8] indicated that high levels of IL-1*β* result in inflammation and endothelial dysfunction, which contribute to vascular access (VA) stenosis and a shorter duration of use. In our study, after AVF was established, the levels of TGF-*β*, IL-1*β*, and IL-18 were significantly increased, and the high expression of HO-1 and PPAR-*γ* evidently reduced their levels in AVF. All these results imply that HO-1/PPAR-*γ* may improve AVF function by regulating the inflammatory response (decreasing TGF-*β*, IL-1*β*, and IL-18 levels).

In addition, the elevation of ROS levels in cells reflects oxidative stress, resulting in damage to lipids, proteins, and DNA [38]. Our experiments showed that ROS levels were higher in AVF, and high expression of HO-1 and PPAR-*γ* significantly reduced its level compared with HO-1^+/-^+AVF mice, which suggested that AVF could induce oxidative stress, and HO-1 and PPAR-*γ* could inhibit oxidative stress caused by AVF. Chen et al. [39] showed that INF2 deletion could induce cell apoptosis by decreasing ROS content and inhibiting the oxidative stress response. HO-1, a stress protein, plays a protective role in blood vessels by alleviating inflammation, proliferation, fibrosis, and oxidative stress [40]. PPAR-*γ* is a transcription factor that plays an important pleiotropic regulatory role in anti-inflammatory, antioxidative, and phagocyte-mediated clearance [41]. Wang et al. [42] reported that HO-1 could attenuate cell apoptosis induced by heat stress in bovine granulosa cells by reducing ROS production and activating antioxidant reactions. Another study demonstrated that ginsenoside Rg1 inhibited inflammation and neuronal apoptosis by activating the PPAR-*γ*/HO-1 pathway in the hippocampus of rats with cerebral ischemia/reperfusion injury [43]. In this study, HO-1 and PPAR-*γ* were significantly upregulated after AVF establishment in *HO-1* knockdown mice, and COPP pretreatment and HO-1 normal expression further upregulated their expression. MMP9 has been reported to be highly expressed in the intimal hyperplasia of blood vessels and in the inflammatory cells during vascular injury [44, 45]. A previous study indicated that HO-1 knockout enhanced macrophage infiltration in abdominal aortic aneurysm, as well as increased the activity of MMP and upregulated the expression of MMP9 [46]. Our experiments also showed that MMP9 was significantly upregulated in HO-1^+/-^ mice, and AVF decreased MMP9 expression. In addition, COPP pretreatment and HO-1 normal expression further downregulated MMP9. Taken together, the HO-1/PPAR-*γ* pathway may suppress AVF-induced intima hyperplasia and protect the intima of blood vessels by regulating MMP9 and ROS, thus mitigating AVF dysfunction.

However, there are some limitations in this study. Firstly, the specific role of the PPAR signaling pathway, insulin signaling pathway, AMPK signaling pathway, glucagon signaling pathway, IL-17 signaling pathway, and Toll-like receptor signaling pathway based on sequencing in AVF formation requires further investigation. In addition, the conclusions of our research need to be further confirmed by *in vivo* xenotransplantation experiments and should be further explored in the clinical practice, using different therapies, to enhance the AVF duration in dialysis patients in the next future.

## 5. Conclusions

In conclusion, by sequencing, 2514 DEmRNAs were identified between the AVF and NC groups, and the oxidative stress response and inflammatory response may be closely related to AVF construction and progression. Additionally, it was found that activation of the HO-1/PPAR-*γ* pathway may protect the vascular intima and mitigate AVF dysfunction by regulating oxidative stress and the inflammatory response. Our work helps improve our understanding of AVF progression and provides a basis for HO-1/PPAR-*γ* as novel potential targets and pathways for improving AVF dysfunction.

## Figures and Tables

**Table 1:** The sequences of all primers.

Primer	Sequence (5′-3′)
PPAR-*γ*	F: CTCCAAGAATACCAAAGTGCGA
	R: GCCTGATGCTTTATCCCCACA
MMP9	F: GCGTCGTGATCCCCACTTAC
	R: CAGGCCGAATAGGAGCGTC
HO-1	F: CGGGCCAGCAACAAAGTA
	R: AGTGTAAGGACCCATCGGAGAC
*β*-Actin	F: GACCCAGATCATGTTTGAGACCT
	R: TCCAGGGAGGAAGAGGATGC

**Table 2:** The contents of serum biochemical indexes in different groups.

Group	Indexes					
	TC (mmol/L)	TG (mmol/L)	HDL (mmol/L)	LDL (mmol/L)	TBIL (*μ*mol/L)	APO-A (g/L)
HO^+/-^+sham	4.05 ± 0.05	1.78 ± 0.42	5.12 ± 0.33	0.45 ± 0.04	12.10 ± 0.17	1.00 ± 0.01
HO^+/-^+AVF	3.39 ± 0.47^∗^	1.56 ± 0.49	3.78 ± 0.29^∗^	0.62 ± 0.09^∗^	13.71 ± 0.87^∗^	1.00 ± 0.01
HO^+/-^+AVF+COPP	3.93 ± 0.17^#^	1.98 ± 0.33	4.42 ± 0.18^∗^^,#^	0.55 ± 0.06	12.45 ± 0.52	0.99 ± 0.004
HO^+/+^+AVF	3.27 ± 0.44^∗^^,$^	1.14 ± 0.22	3.90 ± 0.12^∗^^,$^	0.45 ± 0.04^#^	12.82 ± 0.47	1.00 ± 0.01

TC: total cholesterol; TG: triglycerides; HDL: high-density lipoprotein; LDL: low-density lipoprotein; APO-A: lipoprotein A; TBIL: total bilirubin levels. ^∗^*P* < 0.05, compared with the HO-1^+/-^+sham group; ^#^*P* < 0.05, compared with the HO-1^+/-^+AVF group; ^$^*P* < 0.05, compared with the HO-1^+/-^+AVF+COPP group.

## Data Availability

The data supporting the findings of this study are available from the corresponding author upon reasonable request.

## Supplementary Materials

Supplementary Materials Supplementary Table 1: all differentially expressed mRNAs (DEmRNAs). Supplementary Table 2: all GO terms of DEmRNAs. Supplementary Table 3: all KEGG pathways of downregulated mRNAs. Supplementary Table 4: all KEGG pathways of upregulated mRNAs.
