# Supplementary material for: Heme Oxygenase 1/Peroxisome Proliferator-Activated Receptor Gamma Pathway Protects Intimal Hyperplasia and Mitigates Arteriovenous Fistula Dysfunction by Regulating Oxidative Stress and Inflammatory Response
Source: Cardiovasc Ther. 2022 Jun 12;2022:7576388. doi: 10.1155/2022/7576388 (PMC9207017; doi:10.1155/2022/7576388)
Supplement: Supplementary Materials — Supplementary Table 1: all differentially expressed mRNAs (DEmRNAs). Supplementary Table 2: all GO terms of DEmRNAs. Supplementary Table 3: all KEGG pathways of downregulated mRNAs. Supplementary Table 4: all KEGG pathways of upregulated mRNAs. [file 7576388.f1.docx]

**Supplementary table 1** All differentially expressed mRNAs (DEmRNAs)

| gene_id | log2FoldChange | pvalue | padj | gene_name |
| --- | --- | --- | --- | --- |
| ENSMUSG00000055254 | -3.18666 | 9.92E-48 | 1.90E-43 | Ntrk2 |
| ENSMUSG00000046818 | 5.66684 | 1.03E-39 | 9.83E-36 | Ddit4l |
| ENSMUSG00000020917 | -3.34449 | 4.97E-35 | 3.07E-31 | Acly |
| ENSMUSG00000041220 | -4.407 | 6.41E-35 | 3.07E-31 | Elovl6 |
| ENSMUSG00000033174 | -2.48761 | 2.59E-33 | 9.92E-30 | Mgll |
| ENSMUSG00000069372 | 5.234918 | 4.72E-32 | 1.51E-28 | Ctxn3 |
| ENSMUSG00000030785 | 6.820049 | 6.13E-32 | 1.68E-28 | Cox6a2 |
| ENSMUSG00000024990 | -3.63245 | 2.82E-29 | 6.75E-26 | Rbp4 |
| ENSMUSG00000029482 | -3.0942 | 2.69E-27 | 5.74E-24 | Aacs |
| ENSMUSG00000021898 | 6.931024 | 8.78E-27 | 1.68E-23 | Asb14 |
| ENSMUSG00000030401 | 5.246531 | 1.82E-26 | 3.16E-23 | Rtn2 |
| ENSMUSG00000048416 | 6.730501 | 2.16E-26 | 3.45E-23 | Mlf1 |
| ENSMUSG00000021815 | 6.857602 | 3.47E-26 | 5.12E-23 | Mss51 |
| ENSMUSG00000031097 | 7.195634 | 9.79E-26 | 1.34E-22 | Tnni2 |
| ENSMUSG00000004939 | 9.47053 | 2.09E-25 | 2.67E-22 | Nmrk2 |
| ENSMUSG00000111942 | -4.27536 | 3.54E-25 | 4.24E-22 | Gm5182 |
| ENSMUSG00000030433 | 6.365722 | 4.54E-25 | 5.12E-22 | Sbk2 |
| ENSMUSG00000027605 | -3.75856 | 1.95E-24 | 2.07E-21 | Acss2 |
| ENSMUSG00000020722 | 6.987726 | 2.90E-24 | 2.90E-21 | Cacng1 |
| ENSMUSG00000061723 | 7.123895 | 3.03E-24 | 2.90E-21 | Tnnt3 |
| ENSMUSG00000113178 | 6.676656 | 3.38E-24 | 3.08E-21 | Mylf-ps |
| ENSMUSG00000047822 | -4.80402 | 7.03E-24 | 6.13E-21 | Angptl8 |
| ENSMUSG00000061816 | 6.595987 | 9.79E-24 | 8.16E-21 | Myl1 |
| ENSMUSG00000079017 | -1.72243 | 1.46E-23 | 1.14E-20 | Ifi27l2a |
| ENSMUSG00000020532 | -4.48361 | 1.48E-23 | 1.14E-20 | Acaca |
| ENSMUSG00000047343 | 8.252116 | 4.50E-23 | 3.32E-20 | Mettl21c |
| ENSMUSG00000003528 | -3.73693 | 9.55E-23 | 6.78E-20 | Slc25a1 |
| ENSMUSG00000032495 | 5.708432 | 2.28E-22 | 1.56E-19 | Lrrc2 |
| ENSMUSG00000025792 | -2.66639 | 3.83E-22 | 2.53E-19 | Slc25a10 |
| ENSMUSG00000033213 | 4.421204 | 5.86E-22 | 3.75E-19 | AA467197 |
| ENSMUSG00000028396 | 7.247754 | 6.64E-22 | 4.11E-19 | 2310002L09Rik |
| ENSMUSG00000025153 | -4.90589 | 7.25E-22 | 4.34E-19 | Fasn |
| ENSMUSG00000078234 | -3.62427 | 8.16E-22 | 4.74E-19 | Klhdc7a |
| ENSMUSG00000032366 | 4.932777 | 1.56E-21 | 8.80E-19 | Tpm1 |
| ENSMUSG00000063821 | 7.819133 | 2.17E-21 | 1.19E-18 | Dupd1 |
| ENSMUSG00000031519 | 5.674604 | 2.45E-21 | 1.31E-18 | Asb5 |
| ENSMUSG00000101655 | 4.169488 | 5.25E-21 | 2.72E-18 | 2310040G24Rik |
| ENSMUSG00000075296 | -2.87099 | 7.42E-21 | 3.74E-18 | Aldh3b2 |
| ENSMUSG00000057614 | -3.25951 | 1.10E-20 | 5.39E-18 | Gnai1 |
| ENSMUSG00000025537 | 4.536442 | 1.64E-20 | 7.86E-18 | Phkg1 |
| ENSMUSG00000021768 | 5.999764 | 6.74E-20 | 3.15E-17 | Dusp13 |
| ENSMUSG00000005716 | 7.02548 | 7.22E-20 | 3.29E-17 | Pvalb |
| ENSMUSG00000007877 | 6.252276 | 8.15E-20 | 3.63E-17 | Tcap |
| ENSMUSG00000070424 | 5.769893 | 8.38E-20 | 3.65E-17 | Art5 |
| ENSMUSG00000085348 | 8.17123 | 9.04E-20 | 3.85E-17 | Myhas |
| ENSMUSG00000033065 | 4.514604 | 9.30E-20 | 3.87E-17 | Pfkm |
| ENSMUSG00000038239 | 5.746308 | 1.37E-19 | 5.58E-17 | Hrc |
| ENSMUSG00000031489 | -4.11051 | 1.91E-19 | 7.64E-17 | Adrb3 |
| ENSMUSG00000062694 | 4.606202 | 2.25E-19 | 8.79E-17 | Cav3 |
| ENSMUSG00000026173 | 5.664471 | 3.81E-19 | 1.45E-16 | Plcd4 |
| ENSMUSG00000042834 | 3.43403 | 3.85E-19 | 1.45E-16 | Nrep |
| ENSMUSG00000030774 | 2.246015 | 4.19E-19 | 1.54E-16 | Pak1 |
| ENSMUSG00000007122 | 6.436514 | 4.39E-19 | 1.59E-16 | Casq1 |
| ENSMUSG00000042459 | -13.4945 | 7.91E-19 | 2.81E-16 | Bpifa2 |
| ENSMUSG00000039891 | 5.265009 | 2.44E-18 | 8.49E-16 | Txlnb |
| ENSMUSG00000068697 | 6.97581 | 2.65E-18 | 9.08E-16 | Myoz1 |
| ENSMUSG00000060600 | 6.276375 | 2.90E-18 | 9.74E-16 | Eno3 |
| ENSMUSG00000047182 | -3.27801 | 4.33E-18 | 1.43E-15 | Irs3 |
| ENSMUSG00000021798 | 4.899951 | 4.60E-18 | 1.49E-15 | Ldb3 |
| ENSMUSG00000025203 | -2.0834 | 4.94E-18 | 1.58E-15 | Scd2 |
| ENSMUSG00000068614 | 5.362709 | 5.30E-18 | 1.66E-15 | Actc1 |
| ENSMUSG00000047746 | 4.94154 | 5.48E-18 | 1.69E-15 | Fbxo40 |
| ENSMUSG00000051456 | 8.236671 | 8.28E-18 | 2.52E-15 | Hspb3 |
| ENSMUSG00000001508 | 4.94717 | 1.38E-17 | 4.13E-15 | Sgca |
| ENSMUSG00000020963 | -3.41798 | 1.51E-17 | 4.46E-15 | Tshr |
| ENSMUSG00000032648 | 5.584847 | 1.55E-17 | 4.50E-15 | Pygm |
| ENSMUSG00000039376 | 5.407434 | 1.66E-17 | 4.75E-15 | Synpo2l |
| ENSMUSG00000059089 | 3.493765 | 2.12E-17 | 5.98E-15 | Fcgr4 |
| ENSMUSG00000005628 | 6.464338 | 3.71E-17 | 1.03E-14 | Tmod4 |
| ENSMUSG00000079588 | 3.782865 | 3.91E-17 | 1.07E-14 | Tmem182 |
| ENSMUSG00000011148 | 4.390211 | 4.50E-17 | 1.21E-14 | Adssl1 |
| ENSMUSG00000030672 | 6.869696 | 4.84E-17 | 1.29E-14 | Mylpf |
| ENSMUSG00000053398 | -1.86552 | 9.89E-17 | 2.60E-14 | Phgdh |
| ENSMUSG00000042254 | 3.891516 | 1.21E-16 | 3.12E-14 | Cilp |
| ENSMUSG00000108322 | 4.955789 | 1.35E-16 | 3.45E-14 | 5430431A17Rik |
| ENSMUSG00000046794 | -3.69455 | 1.54E-16 | 3.89E-14 | Ppp1r3b |
| ENSMUSG00000046345 | 6.980129 | 2.46E-16 | 6.12E-14 | Smco1 |
| ENSMUSG00000024747 | -3.83184 | 2.51E-16 | 6.16E-14 | Aldh1a7 |
| ENSMUSG00000071342 | 6.618384 | 4.97E-16 | 1.21E-13 | Lsmem1 |
| ENSMUSG00000047419 | 5.213828 | 7.84E-16 | 1.88E-13 | Cmya5 |
| ENSMUSG00000026817 | 4.009126 | 8.66E-16 | 2.05E-13 | Ak1 |
| ENSMUSG00000026922 | -3.55798 | 9.07E-16 | 2.12E-13 | Agpat2 |
| ENSMUSG00000038204 | 4.524483 | 1.10E-15 | 2.55E-13 | Asb10 |
| ENSMUSG00000020475 | 6.344978 | 1.20E-15 | 2.73E-13 | Pgam2 |
| ENSMUSG00000085779 | 4.615911 | 1.31E-15 | 2.95E-13 | Atcayos |
| ENSMUSG00000026208 | 4.29119 | 1.35E-15 | 3.00E-13 | Des |
| ENSMUSG00000022206 | -2.84865 | 2.51E-15 | 5.52E-13 | Npr3 |
| ENSMUSG00000015970 | -3.67981 | 2.65E-15 | 5.76E-13 | Chdh |
| ENSMUSG00000023828 | -2.92421 | 2.67E-15 | 5.76E-13 | Slc22a3 |
| ENSMUSG00000055546 | -4.14656 | 3.97E-15 | 8.45E-13 | Timd4 |
| ENSMUSG00000056900 | 4.371834 | 4.27E-15 | 9.00E-13 | Usp13 |
| ENSMUSG00000019933 | 5.376563 | 6.62E-15 | 1.38E-12 | Mrln |
| ENSMUSG00000040666 | 4.224342 | 6.75E-15 | 1.38E-12 | Sh3bgr |
| ENSMUSG00000048142 | -3.79103 | 6.75E-15 | 1.38E-12 | Nat8l |
| ENSMUSG00000041476 | 6.755015 | 8.20E-15 | 1.65E-12 | Smpx |
| ENSMUSG00000028464 | 4.227579 | 9.90E-15 | 1.98E-12 | Tpm2 |
| ENSMUSG00000037071 | -5.07194 | 1.14E-14 | 2.26E-12 | Scd1 |
| ENSMUSG00000027470 | 5.395344 | 1.57E-14 | 3.06E-12 | Mylk2 |
| ENSMUSG00000035376 | -2.12915 | 1.80E-14 | 3.49E-12 | Hacd2 |
| ENSMUSG00000025934 | -4.00671 | 1.93E-14 | 3.71E-12 | Gsta3 |
| ENSMUSG00000079278 | 6.390947 | 2.29E-14 | 4.35E-12 | Tmem233 |
| ENSMUSG00000079055 | 3.251959 | 3.12E-14 | 5.83E-12 | Slc8a3 |
| ENSMUSG00000033044 | 6.444568 | 3.13E-14 | 5.83E-12 | Dhrs7c |
| ENSMUSG00000020882 | 3.427541 | 3.62E-14 | 6.66E-12 | Cacnb1 |
| ENSMUSG00000044938 | 5.861972 | 3.93E-14 | 7.17E-12 | Klhl31 |
| ENSMUSG00000025141 | 4.980784 | 4.48E-14 | 8.06E-12 | Myadml2 |
| ENSMUSG00000034641 | 2.344583 | 4.50E-14 | 8.06E-12 | Cd300ld |
| ENSMUSG00000040287 | 5.43785 | 4.56E-14 | 8.08E-12 | Stac3 |
| ENSMUSG00000031382 | 6.257025 | 4.80E-14 | 8.45E-12 | Asb11 |
| ENSMUSG00000001604 | 3.451736 | 5.01E-14 | 8.73E-12 | Tcea3 |
| ENSMUSG00000021957 | -2.62215 | 5.49E-14 | 9.40E-12 | Tkt |
| ENSMUSG00000041202 | -3.41541 | 5.50E-14 | 9.40E-12 | Pla2g2d |
| ENSMUSG00000025006 | -2.56396 | 5.86E-14 | 9.93E-12 | Sorbs1 |
| ENSMUSG00000033022 | -3.69015 | 6.31E-14 | 1.06E-11 | Cdo1 |
| ENSMUSG00000030399 | 6.727103 | 7.30E-14 | 1.22E-11 | Ckm |
| ENSMUSG00000066361 | -2.09015 | 7.55E-14 | 1.25E-11 | Serpina3c |
| ENSMUSG00000043122 | -4.21559 | 8.33E-14 | 1.36E-11 | A530016L24Rik |
| ENSMUSG00000002500 | 6.253973 | 8.49E-14 | 1.38E-11 | Rpl3l |
| ENSMUSG00000031791 | 4.625388 | 9.48E-14 | 1.53E-11 | Tmem38a |
| ENSMUSG00000062778 | -14.7675 | 1.09E-13 | 1.75E-11 | Chia1 |
| ENSMUSG00000029195 | -4.41193 | 1.12E-13 | 1.77E-11 | Klb |
| ENSMUSG00000041695 | 2.638327 | 1.16E-13 | 1.83E-11 | Kcnj2 |
| ENSMUSG00000023078 | -5.18599 | 1.28E-13 | 2.00E-11 | Cxcl13 |
| ENSMUSG00000055430 | -3.99901 | 1.30E-13 | 2.01E-11 | Nap1l5 |
| ENSMUSG00000055027 | 5.65702 | 1.42E-13 | 2.17E-11 | Smyd1 |
| ENSMUSG00000055116 | -1.90509 | 1.45E-13 | 2.21E-11 | Arntl |
| ENSMUSG00000028671 | -2.43612 | 1.62E-13 | 2.45E-11 | Gale |
| ENSMUSG00000030144 | 5.116368 | 1.76E-13 | 2.63E-11 | Clec4d |
| ENSMUSG00000071847 | -2.07762 | 1.80E-13 | 2.67E-11 | Apcdd1 |
| ENSMUSG00000031780 | 5.140882 | 2.18E-13 | 3.21E-11 | Ccl17 |
| ENSMUSG00000056973 | -3.03741 | 2.80E-13 | 4.09E-11 | Ces1d |
| ENSMUSG00000100599 | 3.603023 | 3.02E-13 | 4.38E-11 | 1700120C14Rik |
| ENSMUSG00000028328 | 3.984788 | 3.24E-13 | 4.67E-11 | Tmod1 |
| ENSMUSG00000042895 | 3.763938 | 3.34E-13 | 4.78E-11 | Abra |
| ENSMUSG00000025129 | 5.659098 | 3.43E-13 | 4.86E-11 | Ppp1r27 |
| ENSMUSG00000022519 | 4.750823 | 3.52E-13 | 4.96E-11 | Srl |
| ENSMUSG00000107585 | 9.367964 | 4.11E-13 | 5.75E-11 | 3300002P13Rik |
| ENSMUSG00000030088 | -2.36008 | 4.22E-13 | 5.87E-11 | Aldh1l1 |
| ENSMUSG00000025190 | 2.68151 | 4.35E-13 | 5.97E-11 | Got1 |
| ENSMUSG00000031204 | 5.370264 | 4.36E-13 | 5.97E-11 | Asb12 |
| ENSMUSG00000071657 | -1.68092 | 4.57E-13 | 6.22E-11 | Bscl2 |
| ENSMUSG00000027010 | 2.285102 | 4.93E-13 | 6.65E-11 | Slc25a12 |
| ENSMUSG00000018740 | -2.3503 | 7.00E-13 | 9.37E-11 | Slc25a35 |
| ENSMUSG00000021403 | 4.469443 | 7.53E-13 | 9.99E-11 | Serpinb9b |
| ENSMUSG00000000253 | 3.17462 | 7.56E-13 | 9.99E-11 | Gmpr |
| ENSMUSG00000010492 | 4.359039 | 7.96E-13 | 1.04E-10 | Uckl1os |
| ENSMUSG00000000392 | 2.968985 | 8.16E-13 | 1.06E-10 | Fap |
| ENSMUSG00000040653 | 3.80873 | 8.28E-13 | 1.07E-10 | Ppp1r14c |
| ENSMUSG00000029455 | -1.62133 | 8.90E-13 | 1.14E-10 | Aldh2 |
| ENSMUSG00000042451 | 4.784018 | 9.37E-13 | 1.20E-10 | Mybph |
| ENSMUSG00000029685 | 5.618869 | 9.52E-13 | 1.21E-10 | Asb15 |
| ENSMUSG00000031312 | 6.182604 | 9.83E-13 | 1.24E-10 | Itgb1bp2 |
| ENSMUSG00000087410 | 6.449023 | 9.88E-13 | 1.24E-10 | 2310065F04Rik |
| ENSMUSG00000086298 | 5.42411 | 1.00E-12 | 1.24E-10 | Gm11716 |
| ENSMUSG00000054013 | -3.73362 | 1.06E-12 | 1.31E-10 | Tmem179 |
| ENSMUSG00000067235 | -4.5944 | 1.08E-12 | 1.33E-10 | H2-Q10 |
| ENSMUSG00000031633 | 2.848616 | 1.18E-12 | 1.44E-10 | Slc25a4 |
| ENSMUSG00000041653 | -4.38938 | 1.24E-12 | 1.51E-10 | Pnpla3 |
| ENSMUSG00000040694 | 5.405458 | 1.25E-12 | 1.51E-10 | Apobec2 |
| ENSMUSG00000038170 | 2.685546 | 1.38E-12 | 1.66E-10 | Pde4dip |
| ENSMUSG00000008658 | 5.063011 | 1.41E-12 | 1.68E-10 | Rbfox1 |
| ENSMUSG00000003476 | 3.191793 | 1.44E-12 | 1.71E-10 | Crhr2 |
| ENSMUSG00000006457 | 6.067151 | 1.63E-12 | 1.92E-10 | Actn3 |
| ENSMUSG00000030972 | -4.87139 | 1.69E-12 | 1.98E-10 | Acsm5 |
| ENSMUSG00000006435 | 4.273307 | 2.29E-12 | 2.66E-10 | Neurl1a |
| ENSMUSG00000028834 | 5.802837 | 2.43E-12 | 2.81E-10 | Trim63 |
| ENSMUSG00000044951 | 4.85188 | 2.45E-12 | 2.82E-10 | Mylk4 |
| ENSMUSG00000026564 | 4.341231 | 2.50E-12 | 2.85E-10 | Dusp27 |
| ENSMUSG00000040037 | -2.39057 | 2.57E-12 | 2.91E-10 | Negr1 |
| ENSMUSG00000060429 | -2.44269 | 2.61E-12 | 2.95E-10 | Sntb1 |
| ENSMUSG00000079110 | 4.980965 | 2.76E-12 | 3.09E-10 | Capn3 |
| ENSMUSG00000022126 | 5.118263 | 2.85E-12 | 3.18E-10 | Acod1 |
| ENSMUSG00000040016 | -4.23437 | 2.95E-12 | 3.27E-10 | Ptger3 |
| ENSMUSG00000029664 | 2.303427 | 3.10E-12 | 3.42E-10 | Tfpi2 |
| ENSMUSG00000059824 | 2.384439 | 3.23E-12 | 3.53E-10 | Dbp |
| ENSMUSG00000081194 | 6.132165 | 3.32E-12 | 3.62E-10 | Gm8424 |
| ENSMUSG00000020402 | 2.205121 | 3.44E-12 | 3.72E-10 | Vdac1 |
| ENSMUSG00000028976 | -4.89719 | 3.48E-12 | 3.75E-10 | Slc2a5 |
| ENSMUSG00000045776 | 2.537955 | 3.61E-12 | 3.87E-10 | Lrtm1 |
| ENSMUSG00000020333 | 3.498157 | 3.69E-12 | 3.92E-10 | Acsl6 |
| ENSMUSG00000049173 | 6.020781 | 3.87E-12 | 4.07E-10 | Myoz3 |
| ENSMUSG00000095438 | 6.742138 | 3.87E-12 | 4.07E-10 | Mir133a-1hg |
| ENSMUSG00000024679 | 2.101618 | 4.11E-12 | 4.31E-10 | Ms4a6d |
| ENSMUSG00000084843 | 6.12853 | 4.49E-12 | 4.68E-10 | B230312C02Rik |
| ENSMUSG00000021388 | 2.864715 | 5.01E-12 | 5.19E-10 | Aspn |
| ENSMUSG00000065987 | -4.26564 | 5.12E-12 | 5.28E-10 | Cd209b |
| ENSMUSG00000001918 | -1.98897 | 6.09E-12 | 6.24E-10 | Slc1a5 |
| ENSMUSG00000042686 | 4.609882 | 7.07E-12 | 7.20E-10 | Jph1 |
| ENSMUSG00000062077 | 5.52076 | 7.74E-12 | 7.85E-10 | Trim54 |
| ENSMUSG00000035606 | 4.284929 | 7.96E-12 | 8.03E-10 | Ky |
| ENSMUSG00000030996 | 6.001441 | 8.24E-12 | 8.27E-10 | Art1 |
| ENSMUSG00000021069 | -2.31839 | 9.04E-12 | 9.03E-10 | Pygl |
| ENSMUSG00000045294 | -2.8748 | 9.79E-12 | 9.72E-10 | Insig1 |
| ENSMUSG00000037151 | 3.084781 | 1.01E-11 | 1.00E-09 | Lrrc20 |
| ENSMUSG00000046764 | -4.70671 | 1.02E-11 | 1.01E-09 | A530053G22Rik |
| ENSMUSG00000049265 | -2.44429 | 1.22E-11 | 1.20E-09 | Kcnk3 |
| ENSMUSG00000031373 | -2.34605 | 1.37E-11 | 1.34E-09 | Car5b |
| ENSMUSG00000064225 | -3.76784 | 1.47E-11 | 1.42E-09 | Paqr9 |
| ENSMUSG00000074001 | 5.796189 | 1.52E-11 | 1.47E-09 | Klhl40 |
| ENSMUSG00000027077 | 5.726769 | 1.56E-11 | 1.50E-09 | Smtnl1 |
| ENSMUSG00000032018 | -1.83983 | 1.60E-11 | 1.52E-09 | Sc5d |
| ENSMUSG00000027750 | 3.534614 | 1.61E-11 | 1.52E-09 | Postn |
| ENSMUSG00000022215 | 5.674895 | 1.65E-11 | 1.55E-09 | Fitm1 |
| ENSMUSG00000023832 | -2.32833 | 1.65E-11 | 1.55E-09 | Acat2 |
| ENSMUSG00000026077 | -3.21856 | 1.67E-11 | 1.56E-09 | Npas2 |
| ENSMUSG00000021792 | -3.07143 | 1.74E-11 | 1.62E-09 | Fam213a |
| ENSMUSG00000084929 | 4.814635 | 1.88E-11 | 1.74E-09 | Foxo6os |
| ENSMUSG00000051980 | 6.760586 | 1.98E-11 | 1.82E-09 | Casr |
| ENSMUSG00000022860 | 4.414377 | 2.20E-11 | 2.02E-09 | Chodl |
| ENSMUSG00000033105 | -2.0097 | 2.22E-11 | 2.03E-09 | Lss |
| ENSMUSG00000023044 | -1.89687 | 2.25E-11 | 2.04E-09 | Csad |
| ENSMUSG00000035686 | -4.179 | 2.30E-11 | 2.08E-09 | Thrsp |
| ENSMUSG00000068740 | -2.30704 | 2.48E-11 | 2.23E-09 | Celsr2 |
| ENSMUSG00000097487 | 3.102097 | 2.83E-11 | 2.53E-09 | Ptges3l |
| ENSMUSG00000035948 | -2.84746 | 2.85E-11 | 2.54E-09 | Acss3 |
| ENSMUSG00000031543 | 4.664106 | 2.93E-11 | 2.60E-09 | Ank1 |
| ENSMUSG00000009633 | -2.46145 | 3.08E-11 | 2.72E-09 | G0s2 |
| ENSMUSG00000029153 | 2.796316 | 3.20E-11 | 2.81E-09 | Ociad2 |
| ENSMUSG00000024538 | 2.477489 | 3.37E-11 | 2.95E-09 | Ppic |
| ENSMUSG00000055632 | 1.937674 | 3.55E-11 | 3.10E-09 | Hmcn2 |
| ENSMUSG00000033576 | -2.63385 | 3.67E-11 | 3.18E-09 | Apol6 |
| ENSMUSG00000005980 | -11.6858 | 3.73E-11 | 3.22E-09 | Dnase1 |
| ENSMUSG00000032643 | 3.418764 | 3.96E-11 | 3.40E-09 | Fhl3 |
| ENSMUSG00000020538 | -2.22031 | 3.97E-11 | 3.40E-09 | Srebf1 |
| ENSMUSG00000075307 | 6.100635 | 4.16E-11 | 3.55E-09 | Klhl41 |
| ENSMUSG00000036854 | 3.00731 | 4.33E-11 | 3.67E-09 | Hspb6 |
| ENSMUSG00000025885 | -2.58731 | 4.34E-11 | 3.67E-09 | Myo5b |
| ENSMUSG00000028348 | 5.040303 | 4.43E-11 | 3.72E-09 | Cavin4 |
| ENSMUSG00000037613 | 2.140252 | 4.45E-11 | 3.72E-09 | Tnfrsf23 |
| ENSMUSG00000024471 | 6.037892 | 4.81E-11 | 4.00E-09 | Myot |
| ENSMUSG00000027499 | 3.163089 | 4.91E-11 | 4.07E-09 | Pkia |
| ENSMUSG00000072720 | 4.21605 | 5.00E-11 | 4.13E-09 | Myo18b |
| ENSMUSG00000005514 | -1.47915 | 7.50E-11 | 6.15E-09 | Por |
| ENSMUSG00000063873 | -2.24955 | 7.51E-11 | 6.15E-09 | Slc24a3 |
| ENSMUSG00000005124 | 3.289866 | 7.78E-11 | 6.35E-09 | Wisp1 |
| ENSMUSG00000051747 | 5.290472 | 7.89E-11 | 6.41E-09 | Ttn |
| ENSMUSG00000021775 | 1.695829 | 8.76E-11 | 7.08E-09 | Nr1d2 |
| ENSMUSG00000057003 | 6.013011 | 8.88E-11 | 7.15E-09 | Myh4 |
| ENSMUSG00000027221 | -2.32616 | 8.99E-11 | 7.21E-09 | Chst1 |
| ENSMUSG00000050071 | 6.298639 | 9.24E-11 | 7.38E-09 | Bex1 |
| ENSMUSG00000016349 | 5.372752 | 9.53E-11 | 7.58E-09 | Eef1a2 |
| ENSMUSG00000038132 | 3.920778 | 9.62E-11 | 7.62E-09 | Rbm24 |
| ENSMUSG00000034055 | 3.59062 | 1.07E-10 | 8.46E-09 | Phka1 |
| ENSMUSG00000051373 | 3.694706 | 1.25E-10 | 9.81E-09 | Plpp7 |
| ENSMUSG00000002104 | 3.50327 | 1.30E-10 | 1.02E-08 | Rapsn |
| ENSMUSG00000033182 | 4.411362 | 1.35E-10 | 1.05E-08 | Kbtbd12 |
| ENSMUSG00000026621 | -4.04751 | 1.52E-10 | 1.18E-08 | 1-Mar |
| ENSMUSG00000033308 | -1.79395 | 1.75E-10 | 1.35E-08 | Dpyd |
| ENSMUSG00000085653 | 5.309523 | 1.76E-10 | 1.35E-08 | Gm15179 |
| ENSMUSG00000035472 | -4.98324 | 1.76E-10 | 1.35E-08 | Slc25a21 |
| ENSMUSG00000023349 | 3.930138 | 1.81E-10 | 1.38E-08 | Clec4n |
| ENSMUSG00000023456 | 2.649372 | 1.83E-10 | 1.39E-08 | Tpi1 |
| ENSMUSG00000002550 | -2.04972 | 1.99E-10 | 1.51E-08 | Uck1 |
| ENSMUSG00000028359 | -4.19536 | 2.28E-10 | 1.72E-08 | Orm3 |
| ENSMUSG00000057715 | -3.23748 | 2.33E-10 | 1.75E-08 | A830018L16Rik |
| ENSMUSG00000019883 | -2.79737 | 2.38E-10 | 1.78E-08 | Echdc1 |
| ENSMUSG00000028871 | -3.02067 | 2.43E-10 | 1.81E-08 | Rspo1 |
| ENSMUSG00000058914 | 4.83126 | 2.44E-10 | 1.82E-08 | C1qtnf3 |
| ENSMUSG00000025216 | 4.092576 | 2.59E-10 | 1.92E-08 | Lbx1 |
| ENSMUSG00000000031 | 4.684553 | 2.60E-10 | 1.92E-08 | H19 |
| ENSMUSG00000020216 | 5.190929 | 2.68E-10 | 1.97E-08 | Jsrp1 |
| ENSMUSG00000041889 | 3.528801 | 2.72E-10 | 1.99E-08 | Shisa4 |
| ENSMUSG00000074264 | -9.80743 | 2.96E-10 | 2.16E-08 | Amy1 |
| ENSMUSG00000027386 | 2.855937 | 3.01E-10 | 2.18E-08 | Fbln7 |
| ENSMUSG00000030935 | -4.17697 | 3.02E-10 | 2.18E-08 | Acsm3 |
| ENSMUSG00000039956 | -3.8069 | 3.08E-10 | 2.22E-08 | Mrap |
| ENSMUSG00000010064 | 3.089479 | 3.20E-10 | 2.30E-08 | Slc38a3 |
| ENSMUSG00000051906 | -3.06232 | 3.25E-10 | 2.33E-08 | Cd209f |
| ENSMUSG00000076435 | -1.89971 | 3.30E-10 | 2.35E-08 | Acsf2 |
| ENSMUSG00000064105 | -1.57959 | 3.51E-10 | 2.49E-08 | Cnnm2 |
| ENSMUSG00000037112 | -2.46121 | 3.53E-10 | 2.50E-08 | Sik2 |
| ENSMUSG00000006057 | 1.895911 | 3.64E-10 | 2.57E-08 | Atp5g1 |
| ENSMUSG00000046480 | 4.12709 | 3.88E-10 | 2.72E-08 | Scn4b |
| ENSMUSG00000001420 | -2.3901 | 4.00E-10 | 2.80E-08 | Tmem79 |
| ENSMUSG00000063430 | -3.76473 | 4.32E-10 | 3.01E-08 | Wscd2 |
| ENSMUSG00000071540 | 4.257492 | 4.64E-10 | 3.22E-08 | 3425401B19Rik |
| ENSMUSG00000031972 | 5.692371 | 4.66E-10 | 3.22E-08 | Acta1 |
| ENSMUSG00000042476 | 3.626189 | 4.77E-10 | 3.29E-08 | Abcb4 |
| ENSMUSG00000020386 | 2.502088 | 4.84E-10 | 3.33E-08 | Sar1b |
| ENSMUSG00000000142 | -1.91404 | 5.51E-10 | 3.77E-08 | Axin2 |
| ENSMUSG00000039987 | 2.098727 | 5.65E-10 | 3.86E-08 | Phtf2 |
| ENSMUSG00000070385 | 5.679145 | 5.97E-10 | 4.06E-08 | Ampd1 |
| ENSMUSG00000090066 | 4.380186 | 6.06E-10 | 4.11E-08 | 1110002E22Rik |
| ENSMUSG00000031351 | -2.32346 | 6.93E-10 | 4.68E-08 | Zfp185 |
| ENSMUSG00000030806 | -2.08361 | 7.12E-10 | 4.79E-08 | Stx1b |
| ENSMUSG00000079168 | -3.19835 | 7.69E-10 | 5.15E-08 | Cd209g |
| ENSMUSG00000030168 | -1.69007 | 7.83E-10 | 5.23E-08 | Adipor2 |
| ENSMUSG00000074771 | -3.46955 | 8.02E-10 | 5.33E-08 | Ankef1 |
| ENSMUSG00000027887 | 4.846458 | 8.52E-10 | 5.65E-08 | Sypl2 |
| ENSMUSG00000027327 | -1.9886 | 8.75E-10 | 5.78E-08 | 1700037H04Rik |
| ENSMUSG00000022965 | 1.361804 | 9.38E-10 | 6.18E-08 | Ifngr2 |
| ENSMUSG00000024672 | 2.400406 | 9.57E-10 | 6.28E-08 | Ms4a7 |
| ENSMUSG00000021215 | -1.51382 | 9.77E-10 | 6.39E-08 | Net1 |
| ENSMUSG00000044405 | -3.10043 | 1.06E-09 | 6.92E-08 | Adig |
| ENSMUSG00000024892 | -3.44501 | 1.06E-09 | 6.92E-08 | Pcx |
| ENSMUSG00000022197 | -1.72904 | 1.20E-09 | 7.79E-08 | Pdzd2 |
| ENSMUSG00000027107 | 4.142688 | 1.24E-09 | 7.98E-08 | Chrna1 |
| ENSMUSG00000096445 | -16.2145 | 1.24E-09 | 7.98E-08 | Dcpp1 |
| ENSMUSG00000039989 | -1.46998 | 1.35E-09 | 8.63E-08 | Cbx4 |
| ENSMUSG00000030554 | 2.775651 | 1.38E-09 | 8.82E-08 | Synm |
| ENSMUSG00000038403 | 5.395256 | 1.53E-09 | 9.74E-08 | Hfe2 |
| ENSMUSG00000022525 | 4.697518 | 1.54E-09 | 9.77E-08 | Hrasls |
| ENSMUSG00000012705 | -3.01991 | 1.55E-09 | 9.78E-08 | Retn |
| ENSMUSG00000028584 | 6.407462 | 1.59E-09 | 1.00E-07 | Lrrc38 |
| ENSMUSG00000030695 | 2.754337 | 1.62E-09 | 1.02E-07 | Aldoa |
| ENSMUSG00000027680 | -1.86745 | 1.63E-09 | 1.02E-07 | Fxr1 |
| ENSMUSG00000018930 | 2.818898 | 1.64E-09 | 1.02E-07 | Ccl4 |
| ENSMUSG00000025509 | -2.55887 | 1.64E-09 | 1.02E-07 | Pnpla2 |
| ENSMUSG00000109674 | -3.71081 | 1.69E-09 | 1.05E-07 | Gm45470 |
| ENSMUSG00000019437 | -2.22298 | 1.74E-09 | 1.07E-07 | Tlcd1 |
| ENSMUSG00000019088 | 1.705966 | 1.74E-09 | 1.07E-07 | Dnase1l1 |
| ENSMUSG00000089665 | -2.91963 | 1.85E-09 | 1.14E-07 | Fcor |
| ENSMUSG00000028024 | -1.85608 | 2.16E-09 | 1.32E-07 | Enpep |
| ENSMUSG00000059201 | -4.05021 | 2.17E-09 | 1.33E-07 | Lep |
| ENSMUSG00000116056 | 3.099803 | 2.32E-09 | 1.41E-07 | Gm4544 |
| ENSMUSG00000051048 | 3.392993 | 2.37E-09 | 1.44E-07 | P4ha3 |
| ENSMUSG00000039349 | -1.44962 | 2.43E-09 | 1.46E-07 | C130074G19Rik |
| ENSMUSG00000026858 | -1.85262 | 2.43E-09 | 1.46E-07 | Miga2 |
| ENSMUSG00000006542 | 5.456826 | 2.49E-09 | 1.50E-07 | Prkag3 |
| ENSMUSG00000030546 | -3.62367 | 2.50E-09 | 1.50E-07 | Plin1 |
| ENSMUSG00000027805 | 2.404474 | 2.62E-09 | 1.56E-07 | Pfn2 |
| ENSMUSG00000031673 | 2.485802 | 2.68E-09 | 1.59E-07 | Cdh11 |
| ENSMUSG00000058952 | 5.120386 | 2.73E-09 | 1.62E-07 | Cfi |
| ENSMUSG00000044499 | 8.393562 | 2.95E-09 | 1.75E-07 | Hs3st5 |
| ENSMUSG00000105888 | -15.7854 | 2.97E-09 | 1.75E-07 | BC037156 |
| ENSMUSG00000026938 | -2.26441 | 3.33E-09 | 1.96E-07 | Fcna |
| ENSMUSG00000035131 | 4.789788 | 3.61E-09 | 2.12E-07 | Brinp3 |
| ENSMUSG00000031495 | -3.44783 | 3.81E-09 | 2.23E-07 | Cd209d |
| ENSMUSG00000033400 | 2.872784 | 3.91E-09 | 2.28E-07 | Agl |
| ENSMUSG00000023992 | 3.231828 | 4.12E-09 | 2.39E-07 | Trem2 |
| ENSMUSG00000027257 | 2.682615 | 4.14E-09 | 2.40E-07 | Pacsin3 |
| ENSMUSG00000087523 | 3.54061 | 4.16E-09 | 2.40E-07 | Gm12319 |
| ENSMUSG00000061397 | -2.00845 | 4.22E-09 | 2.43E-07 | Krt79 |
| ENSMUSG00000034652 | 2.398989 | 4.40E-09 | 2.52E-07 | Cd300a |
| ENSMUSG00000024803 | 3.464936 | 4.48E-09 | 2.56E-07 | Ankrd1 |
| ENSMUSG00000024228 | -2.43462 | 4.76E-09 | 2.71E-07 | Nudt12 |
| ENSMUSG00000090799 | 2.835854 | 4.93E-09 | 2.80E-07 | Klhl33 |
| ENSMUSG00000022546 | -2.12592 | 5.19E-09 | 2.94E-07 | Gpt |
| ENSMUSG00000021097 | -2.1535 | 5.28E-09 | 2.99E-07 | Clmn |
| ENSMUSG00000025777 | 5.344661 | 5.31E-09 | 2.99E-07 | Gdap1 |
| ENSMUSG00000029280 | -17.1973 | 5.33E-09 | 2.99E-07 | Smr3a |
| ENSMUSG00000020085 | -2.0965 | 5.38E-09 | 3.02E-07 | Aifm2 |
| ENSMUSG00000056938 | -1.72326 | 5.61E-09 | 3.14E-07 | Acbd4 |
| ENSMUSG00000112329 | 5.989164 | 5.87E-09 | 3.27E-07 | Gm47908 |
| ENSMUSG00000030711 | -1.51269 | 6.05E-09 | 3.36E-07 | Sult1a1 |
| ENSMUSG00000045667 | 3.291561 | 6.17E-09 | 3.42E-07 | Smtnl2 |
| ENSMUSG00000018171 | 1.254393 | 6.30E-09 | 3.48E-07 | Vmp1 |
| ENSMUSG00000045211 | -1.08559 | 6.37E-09 | 3.51E-07 | Nudt18 |
| ENSMUSG00000031448 | 3.72603 | 6.39E-09 | 3.51E-07 | Adprhl1 |
| ENSMUSG00000054434 | -2.28858 | 6.52E-09 | 3.57E-07 | Tmem120b |
| ENSMUSG00000037686 | -4.60748 | 6.57E-09 | 3.59E-07 | Aspg |
| ENSMUSG00000018822 | -4.32211 | 8.19E-09 | 4.46E-07 | Sfrp5 |
| ENSMUSG00000027952 | -1.92005 | 8.38E-09 | 4.55E-07 | Pmvk |
| ENSMUSG00000019194 | 2.357368 | 8.86E-09 | 4.79E-07 | Scn1b |
| ENSMUSG00000028011 | -3.66257 | 9.17E-09 | 4.95E-07 | Tdo2 |
| ENSMUSG00000032418 | -2.849 | 9.72E-09 | 5.23E-07 | Me1 |
| ENSMUSG00000045875 | -2.65207 | 1.01E-08 | 5.43E-07 | Adra1a |
| ENSMUSG00000024617 | 4.705967 | 1.02E-08 | 5.44E-07 | Camk2a |
| ENSMUSG00000024076 | -3.36252 | 1.09E-08 | 5.81E-07 | Vit |
| ENSMUSG00000026950 | 4.90082 | 1.11E-08 | 5.89E-07 | Neb |
| ENSMUSG00000029553 | 1.74367 | 1.12E-08 | 5.95E-07 | Tfec |
| ENSMUSG00000027868 | 2.53778 | 1.14E-08 | 6.03E-07 | Tbx15 |
| ENSMUSG00000021373 | 3.175009 | 1.22E-08 | 6.43E-07 | Cap2 |
| ENSMUSG00000032092 | -3.42717 | 1.23E-08 | 6.46E-07 | Mpzl2 |
| ENSMUSG00000028139 | 2.698827 | 1.23E-08 | 6.47E-07 | Riiad1 |
| ENSMUSG00000044086 | 5.098987 | 1.29E-08 | 6.78E-07 | Lmod3 |
| ENSMUSG00000030579 | 1.839014 | 1.41E-08 | 7.34E-07 | Tyrobp |
| ENSMUSG00000100783 | 5.778254 | 1.50E-08 | 7.79E-07 | 2310047D07Rik |
| ENSMUSG00000020123 | -2.28141 | 1.52E-08 | 7.88E-07 | Avpr1a |
| ENSMUSG00000054204 | -5.02772 | 1.53E-08 | 7.91E-07 | Alkal2 |
| ENSMUSG00000099552 | 7.892577 | 1.53E-08 | 7.91E-07 | 9830004L10Rik |
| ENSMUSG00000012187 | -4.47713 | 1.57E-08 | 8.10E-07 | Mogat1 |
| ENSMUSG00000059146 | -2.73438 | 1.58E-08 | 8.11E-07 | Ntrk3 |
| ENSMUSG00000021702 | 4.280599 | 1.66E-08 | 8.52E-07 | Thbs4 |
| ENSMUSG00000003123 | -2.99781 | 1.69E-08 | 8.63E-07 | Lipe |
| ENSMUSG00000094930 | -6.26303 | 1.73E-08 | 8.84E-07 | Igkv6-25 |
| ENSMUSG00000049134 | 4.509234 | 1.80E-08 | 9.15E-07 | Nrap |
| ENSMUSG00000042429 | -3.61695 | 1.93E-08 | 9.78E-07 | Adora1 |
| ENSMUSG00000006784 | -3.33431 | 2.00E-08 | 1.01E-06 | Ttc25 |
| ENSMUSG00000057719 | 3.052056 | 2.02E-08 | 1.02E-06 | Sh3rf2 |
| ENSMUSG00000020253 | -1.2978 | 2.07E-08 | 1.04E-06 | Ppm1m |
| ENSMUSG00000033068 | -1.81045 | 2.09E-08 | 1.05E-06 | Entpd6 |
| ENSMUSG00000038150 | -1.42641 | 2.10E-08 | 1.05E-06 | Ormdl3 |
| ENSMUSG00000021200 | 3.048718 | 2.11E-08 | 1.06E-06 | Asb2 |
| ENSMUSG00000049112 | -2.2731 | 2.17E-08 | 1.08E-06 | Oxtr |
| ENSMUSG00000019932 | 7.858324 | 2.18E-08 | 1.08E-06 | Kera |
| ENSMUSG00000046844 | -4.43912 | 2.24E-08 | 1.11E-06 | Vat1l |
| ENSMUSG00000039518 | -3.03525 | 2.29E-08 | 1.13E-06 | Cdsn |
| ENSMUSG00000049892 | -3.09673 | 2.33E-08 | 1.15E-06 | Rasd1 |
| ENSMUSG00000061086 | 3.693578 | 2.49E-08 | 1.22E-06 | Myl4 |
| ENSMUSG00000029371 | 4.021511 | 2.53E-08 | 1.24E-06 | Cxcl5 |
| ENSMUSG00000046727 | 2.581768 | 2.57E-08 | 1.26E-06 | Cystm1 |
| ENSMUSG00000113909 | 6.134453 | 2.62E-08 | 1.28E-06 | Gm36377 |
| ENSMUSG00000025981 | 1.07073 | 2.72E-08 | 1.32E-06 | Coq10b |
| ENSMUSG00000008136 | 2.501539 | 2.85E-08 | 1.38E-06 | Fhl2 |
| ENSMUSG00000032523 | 4.446532 | 2.87E-08 | 1.39E-06 | Hhatl |
| ENSMUSG00000061974 | -8.37262 | 3.27E-08 | 1.58E-06 | Cldn24 |
| ENSMUSG00000028393 | -1.53695 | 3.37E-08 | 1.62E-06 | Alad |
| ENSMUSG00000027792 | -2.17283 | 3.47E-08 | 1.67E-06 | Bche |
| ENSMUSG00000039196 | -3.52389 | 3.66E-08 | 1.75E-06 | Orm1 |
| ENSMUSG00000059027 | -2.60092 | 3.74E-08 | 1.79E-06 | 9630013D21Rik |
| ENSMUSG00000046275 | -3.0526 | 3.81E-08 | 1.82E-06 | Tusc5 |
| ENSMUSG00000001773 | -5.69428 | 3.90E-08 | 1.85E-06 | Folh1 |
| ENSMUSG00000089718 | 5.882271 | 3.91E-08 | 1.85E-06 | 2310075C17Rik |
| ENSMUSG00000045246 | 4.758482 | 3.93E-08 | 1.86E-06 | Kcng4 |
| ENSMUSG00000031239 | 2.84694 | 3.94E-08 | 1.86E-06 | Itm2a |
| ENSMUSG00000042251 | -3.45862 | 3.95E-08 | 1.86E-06 | Pm20d1 |
| ENSMUSG00000033156 | -18.182 | 4.00E-08 | 1.88E-06 | Cst10 |
| ENSMUSG00000079293 | 2.994187 | 4.05E-08 | 1.90E-06 | Clec7a |
| ENSMUSG00000030747 | -3.46136 | 4.29E-08 | 2.01E-06 | Dgat2 |
| ENSMUSG00000023074 | 1.297976 | 4.53E-08 | 2.11E-06 | Mospd1 |
| ENSMUSG00000069516 | 2.066055 | 4.53E-08 | 2.11E-06 | Lyz2 |
| ENSMUSG00000065460 | 7.658413 | 4.58E-08 | 2.12E-06 | Mir133a-2 |
| ENSMUSG00000028076 | -2.35532 | 4.92E-08 | 2.28E-06 | Cd1d1 |
| ENSMUSG00000020326 | 2.061483 | 5.21E-08 | 2.40E-06 | Ccng1 |
| ENSMUSG00000031530 | -2.62156 | 5.23E-08 | 2.41E-06 | Dusp4 |
| ENSMUSG00000020277 | -1.6545 | 5.32E-08 | 2.44E-06 | Pfkl |
| ENSMUSG00000042041 | -3.35208 | 5.53E-08 | 2.54E-06 | 2010003K11Rik |
| ENSMUSG00000038642 | 2.309584 | 6.00E-08 | 2.74E-06 | Ctss |
| ENSMUSG00000073412 | 1.822435 | 6.04E-08 | 2.75E-06 | Lst1 |
| ENSMUSG00000019848 | 4.962725 | 6.10E-08 | 2.78E-06 | Popdc3 |
| ENSMUSG00000060913 | 4.96406 | 6.13E-08 | 2.79E-06 | Trim55 |
| ENSMUSG00000040118 | 2.549726 | 6.19E-08 | 2.80E-06 | Cacna2d1 |
| ENSMUSG00000102573 | 6.679702 | 6.39E-08 | 2.89E-06 | Gm7265 |
| ENSMUSG00000114515 | 4.180617 | 6.43E-08 | 2.90E-06 | Aldoa |
| ENSMUSG00000044338 | 1.328147 | 6.47E-08 | 2.91E-06 | Aplnr |
| ENSMUSG00000053411 | -1.18104 | 6.77E-08 | 3.04E-06 | Cbx7 |
| ENSMUSG00000060002 | -2.18083 | 6.95E-08 | 3.11E-06 | Chpt1 |
| ENSMUSG00000099398 | 2.076083 | 6.97E-08 | 3.11E-06 | Ms4a14 |
| ENSMUSG00000036106 | -1.34109 | 7.08E-08 | 3.15E-06 | Prr5 |
| ENSMUSG00000030539 | -1.4106 | 7.25E-08 | 3.22E-06 | Sema4b |
| ENSMUSG00000033544 | 3.733726 | 7.54E-08 | 3.35E-06 | Angptl1 |
| ENSMUSG00000023982 | 1.682049 | 7.92E-08 | 3.50E-06 | Guca1a |
| ENSMUSG00000031906 | -3.38435 | 8.05E-08 | 3.55E-06 | Smpd3 |
| ENSMUSG00000022865 | -2.92867 | 8.99E-08 | 3.96E-06 | Cxadr |
| ENSMUSG00000027022 | 4.999129 | 9.19E-08 | 4.04E-06 | Xirp2 |
| ENSMUSG00000020829 | -1.60127 | 9.29E-08 | 4.08E-06 | Slc46a1 |
| ENSMUSG00000033825 | -2.80575 | 9.92E-08 | 4.34E-06 | Tpsb2 |
| ENSMUSG00000022440 | 2.22787 | 1.02E-07 | 4.44E-06 | C1qtnf6 |
| ENSMUSG00000022303 | 3.975263 | 1.04E-07 | 4.52E-06 | Dcstamp |
| ENSMUSG00000020102 | -1.13684 | 1.06E-07 | 4.62E-06 | Slc16a7 |
| ENSMUSG00000028111 | 2.371848 | 1.07E-07 | 4.63E-06 | Ctsk |
| ENSMUSG00000115759 | 2.572325 | 1.07E-07 | 4.64E-06 | Gm18787 |
| ENSMUSG00000037053 | -8.48084 | 1.09E-07 | 4.70E-06 | Azgp1 |
| ENSMUSG00000026860 | -1.12579 | 1.12E-07 | 4.84E-06 | Sh3glb2 |
| ENSMUSG00000061068 | -2.7134 | 1.15E-07 | 4.94E-06 | Mcpt4 |
| ENSMUSG00000027762 | -3.10606 | 1.16E-07 | 4.99E-06 | Sucnr1 |
| ENSMUSG00000032854 | -4.87854 | 1.18E-07 | 5.03E-06 | Ugt8a |
| ENSMUSG00000075122 | 1.863853 | 1.21E-07 | 5.17E-06 | Cd80 |
| ENSMUSG00000043154 | 2.104842 | 1.23E-07 | 5.25E-06 | Ppp2r3a |
| ENSMUSG00000022676 | 1.466056 | 1.24E-07 | 5.26E-06 | Snai2 |
| ENSMUSG00000024381 | 2.0606 | 1.25E-07 | 5.29E-06 | Bin1 |
| ENSMUSG00000027895 | 2.972962 | 1.25E-07 | 5.31E-06 | Kcnc4 |
| ENSMUSG00000020458 | 1.688676 | 1.29E-07 | 5.43E-06 | Rtn4 |
| ENSMUSG00000067081 | 5.102741 | 1.30E-07 | 5.46E-06 | Asb18 |
| ENSMUSG00000026385 | -2.42115 | 1.30E-07 | 5.48E-06 | Dbi |
| ENSMUSG00000037110 | -1.74724 | 1.36E-07 | 5.70E-06 | Ralgapa2 |
| ENSMUSG00000100502 | 3.636512 | 1.38E-07 | 5.77E-06 | Gm28286 |
| ENSMUSG00000023960 | -1.75994 | 1.41E-07 | 5.90E-06 | Enpp5 |
| ENSMUSG00000026944 | -1.54279 | 1.45E-07 | 6.04E-06 | Abca2 |
| ENSMUSG00000028023 | 4.168358 | 1.45E-07 | 6.04E-06 | Pitx2 |
| ENSMUSG00000038763 | 3.288683 | 1.50E-07 | 6.23E-06 | Alpk3 |
| ENSMUSG00000005413 | 2.203727 | 1.66E-07 | 6.88E-06 | Hmox1 |
| ENSMUSG00000009207 | 1.406951 | 1.66E-07 | 6.88E-06 | Lnpk |
| ENSMUSG00000043126 | 6.543842 | 1.68E-07 | 6.91E-06 | D830039M14Rik |
| ENSMUSG00000067653 | 3.827972 | 1.68E-07 | 6.91E-06 | Ankrd23 |
| ENSMUSG00000041707 | 2.111927 | 1.68E-07 | 6.91E-06 | Tmem273 |
| ENSMUSG00000008892 | 1.382203 | 1.69E-07 | 6.93E-06 | Vdac3 |
| ENSMUSG00000096617 | 2.248223 | 1.70E-07 | 6.95E-06 | Gm5559 |
| ENSMUSG00000046329 | -1.40266 | 1.71E-07 | 6.99E-06 | Slc25a23 |
| ENSMUSG00000014075 | 1.144984 | 1.74E-07 | 7.07E-06 | Tctex1d2 |
| ENSMUSG00000030108 | -2.29038 | 1.77E-07 | 7.17E-06 | Slc6a13 |
| ENSMUSG00000004558 | 2.451579 | 1.80E-07 | 7.28E-06 | Ndrg2 |
| ENSMUSG00000024208 | 1.126673 | 1.85E-07 | 7.48E-06 | Uqcc2 |
| ENSMUSG00000043157 | 2.033559 | 1.87E-07 | 7.53E-06 | Arl11 |
| ENSMUSG00000020087 | -1.18222 | 1.95E-07 | 7.86E-06 | Tysnd1 |
| ENSMUSG00000002997 | -2.3633 | 1.97E-07 | 7.91E-06 | Prkar2b |
| ENSMUSG00000037709 | -2.24571 | 1.99E-07 | 7.99E-06 | Fam13a |
| ENSMUSG00000055541 | 1.699422 | 2.04E-07 | 8.15E-06 | Lair1 |
| ENSMUSG00000020836 | 2.762282 | 2.05E-07 | 8.20E-06 | Coro6 |
| ENSMUSG00000031736 | 2.253982 | 2.07E-07 | 8.27E-06 | Crnde |
| ENSMUSG00000101389 | 1.962361 | 2.13E-07 | 8.47E-06 | Ms4a4a |
| ENSMUSG00000036352 | 2.051439 | 2.14E-07 | 8.48E-06 | Ubac1 |
| ENSMUSG00000030142 | 3.931672 | 2.19E-07 | 8.66E-06 | Clec4e |
| ENSMUSG00000024726 | 1.904942 | 2.21E-07 | 8.75E-06 | Carnmt1 |
| ENSMUSG00000029862 | 4.432418 | 2.23E-07 | 8.78E-06 | Clcn1 |
| ENSMUSG00000103409 | 3.415361 | 2.27E-07 | 8.92E-06 | Lsmem2 |
| ENSMUSG00000040705 | 4.829461 | 2.36E-07 | 9.27E-06 | A930016O22Rik |
| ENSMUSG00000031250 | 3.331194 | 2.38E-07 | 9.32E-06 | Tnmd |
| ENSMUSG00000041697 | -2.08243 | 2.41E-07 | 9.43E-06 | Cox6a1 |
| ENSMUSG00000038550 | 2.941258 | 2.42E-07 | 9.45E-06 | Ciart |
| ENSMUSG00000043953 | 1.485562 | 2.64E-07 | 1.03E-05 | Ccrl2 |
| ENSMUSG00000073988 | -2.81442 | 2.67E-07 | 1.04E-05 | Ttpa |
| ENSMUSG00000050663 | -2.44886 | 2.76E-07 | 1.07E-05 | Trhde |
| ENSMUSG00000041607 | -3.80132 | 2.77E-07 | 1.07E-05 | Mbp |
| ENSMUSG00000097451 | 2.370744 | 2.80E-07 | 1.08E-05 | Rian |
| ENSMUSG00000073002 | 1.269109 | 2.85E-07 | 1.10E-05 | Vamp5 |
| ENSMUSG00000101257 | 4.414005 | 2.86E-07 | 1.10E-05 | 2310015K22Rik |
| ENSMUSG00000028399 | -1.34387 | 2.88E-07 | 1.10E-05 | Ptprd |
| ENSMUSG00000059498 | 1.627369 | 3.05E-07 | 1.17E-05 | Fcgr3 |
| ENSMUSG00000046245 | 2.263247 | 3.11E-07 | 1.19E-05 | Pilra |
| ENSMUSG00000039345 | 1.78813 | 3.18E-07 | 1.21E-05 | Mettl22 |
| ENSMUSG00000045551 | 4.161821 | 3.18E-07 | 1.21E-05 | Fpr1 |
| ENSMUSG00000022048 | 1.4241 | 3.22E-07 | 1.23E-05 | Dpysl2 |
| ENSMUSG00000036438 | 0.893304 | 3.27E-07 | 1.24E-05 | Calm2 |
| ENSMUSG00000068587 | 3.383417 | 3.29E-07 | 1.25E-05 | Mgam |
| ENSMUSG00000018893 | 7.000189 | 3.33E-07 | 1.26E-05 | Mb |
| ENSMUSG00000019927 | 2.170411 | 3.50E-07 | 1.32E-05 | Ube2d1 |
| ENSMUSG00000110613 | -2.79489 | 3.54E-07 | 1.33E-05 | Lncbate1 |
| ENSMUSG00000075053 | 1.696564 | 3.55E-07 | 1.33E-05 | Vdac3-ps1 |
| ENSMUSG00000079105 | -3.21206 | 3.60E-07 | 1.35E-05 | C7 |
| ENSMUSG00000103965 | -4.8646 | 3.63E-07 | 1.36E-05 | Gm30173 |
| ENSMUSG00000087382 | -3.7647 | 3.64E-07 | 1.36E-05 | Ctcflos |
| ENSMUSG00000057457 | -4.11649 | 3.73E-07 | 1.39E-05 | Phex |
| ENSMUSG00000051855 | 3.593947 | 3.76E-07 | 1.40E-05 | Mest |
| ENSMUSG00000036278 | 1.881056 | 3.78E-07 | 1.40E-05 | Macrod1 |
| ENSMUSG00000017817 | 3.154115 | 3.84E-07 | 1.43E-05 | Jph2 |
| ENSMUSG00000032058 | -1.49017 | 3.88E-07 | 1.44E-05 | Ppp2r1b |
| ENSMUSG00000048852 | 2.0223 | 4.08E-07 | 1.51E-05 | Gm12185 |
| ENSMUSG00000025429 | 1.979101 | 4.11E-07 | 1.51E-05 | Pstpip2 |
| ENSMUSG00000020059 | -2.77375 | 4.14E-07 | 1.52E-05 | Sycp3 |
| ENSMUSG00000027398 | 3.139899 | 4.26E-07 | 1.57E-05 | Il1b |
| ENSMUSG00000034220 | 3.1546 | 4.39E-07 | 1.61E-05 | Gpc1 |
| ENSMUSG00000023913 | 1.817765 | 4.39E-07 | 1.61E-05 | Pla2g7 |
| ENSMUSG00000021223 | -2.04111 | 4.73E-07 | 1.73E-05 | Papln |
| ENSMUSG00000020390 | 1.596101 | 4.75E-07 | 1.73E-05 | Ube2b |
| ENSMUSG00000022555 | -1.91802 | 4.84E-07 | 1.76E-05 | Dgat1 |
| ENSMUSG00000032348 | -1.88567 | 4.99E-07 | 1.81E-05 | Gsta4 |
| ENSMUSG00000000440 | 2.706334 | 5.28E-07 | 1.91E-05 | Pparg |
| ENSMUSG00000033610 | -1.94144 | 5.29E-07 | 1.91E-05 | Pank1 |
| ENSMUSG00000087080 | 7.956389 | 5.38E-07 | 1.94E-05 | Gm12199 |
| ENSMUSG00000049225 | 1.584648 | 5.51E-07 | 1.98E-05 | Pdp1 |
| ENSMUSG00000002228 | 2.976995 | 5.53E-07 | 1.99E-05 | Ppm1j |
| ENSMUSG00000031637 | 4.266273 | 5.63E-07 | 2.02E-05 | Lrp2bp |
| ENSMUSG00000020010 | -2.24692 | 5.73E-07 | 2.05E-05 | Vnn3 |
| ENSMUSG00000030630 | -1.83944 | 5.79E-07 | 2.07E-05 | Fah |
| ENSMUSG00000009471 | 4.638615 | 5.94E-07 | 2.12E-05 | Myod1 |
| ENSMUSG00000049241 | -3.03406 | 6.12E-07 | 2.18E-05 | Hcar1 |
| ENSMUSG00000025277 | -1.40716 | 6.18E-07 | 2.20E-05 | Abhd6 |
| ENSMUSG00000030592 | 4.083959 | 6.24E-07 | 2.21E-05 | Ryr1 |
| ENSMUSG00000100410 | 6.206824 | 6.28E-07 | 2.23E-05 | 2310020H05Rik |
| ENSMUSG00000044811 | 2.860203 | 6.31E-07 | 2.23E-05 | Cd300c2 |
| ENSMUSG00000053192 | 1.944855 | 6.37E-07 | 2.25E-05 | Mllt11 |
| ENSMUSG00000018845 | 2.787556 | 6.41E-07 | 2.26E-05 | Unc45b |
| ENSMUSG00000038663 | 2.663053 | 6.61E-07 | 2.32E-05 | Fsd2 |
| ENSMUSG00000030545 | -2.09619 | 6.80E-07 | 2.39E-05 | Pex11a |
| ENSMUSG00000033006 | -2.96136 | 6.85E-07 | 2.40E-05 | Sox10 |
| ENSMUSG00000114088 | 6.03595 | 7.01E-07 | 2.45E-05 | Gm47898 |
| ENSMUSG00000078486 | 2.534764 | 7.08E-07 | 2.47E-05 | Perm1 |
| ENSMUSG00000022853 | -1.67981 | 7.17E-07 | 2.50E-05 | Ehhadh |
| ENSMUSG00000001131 | 2.489628 | 7.78E-07 | 2.70E-05 | Timp1 |
| ENSMUSG00000005360 | -1.83226 | 7.79E-07 | 2.70E-05 | Slc1a3 |
| ENSMUSG00000038388 | 1.323626 | 7.84E-07 | 2.72E-05 | Mpp6 |
| ENSMUSG00000034457 | 2.11773 | 7.89E-07 | 2.73E-05 | Eda2r |
| ENSMUSG00000021622 | 6.415611 | 8.00E-07 | 2.76E-05 | Ckmt2 |
| ENSMUSG00000037455 | -1.29386 | 8.00E-07 | 2.76E-05 | Slc18b1 |
| ENSMUSG00000052270 | 1.966086 | 8.05E-07 | 2.77E-05 | Fpr2 |
| ENSMUSG00000086432 | -5.12011 | 8.33E-07 | 2.86E-05 | B430119L08Rik |
| ENSMUSG00000025917 | 1.095859 | 8.33E-07 | 2.86E-05 | Cops5 |
| ENSMUSG00000032845 | 4.328083 | 8.37E-07 | 2.86E-05 | Alpk2 |
| ENSMUSG00000003526 | -2.02877 | 8.41E-07 | 2.87E-05 | Prodh |
| ENSMUSG00000029610 | 2.257029 | 8.58E-07 | 2.92E-05 | Aimp2 |
| ENSMUSG00000035896 | -6.11837 | 8.58E-07 | 2.92E-05 | Rnase1 |
| ENSMUSG00000048911 | -1.7317 | 9.67E-07 | 3.29E-05 | Rnf24 |
| ENSMUSG00000003948 | -1.92 | 9.88E-07 | 3.35E-05 | Mmd |
| ENSMUSG00000040564 | -2.93918 | 9.94E-07 | 3.36E-05 | Apoc1 |
| ENSMUSG00000002102 | 0.802414 | 9.97E-07 | 3.37E-05 | Psmc3 |
| ENSMUSG00000062070 | 1.99311 | 1.05E-06 | 3.54E-05 | Pgk1 |
| ENSMUSG00000039496 | 2.165837 | 1.06E-06 | 3.57E-05 | Cdnf |
| ENSMUSG00000026043 | 1.888155 | 1.09E-06 | 3.65E-05 | Col3a1 |
| ENSMUSG00000110547 | 4.936477 | 1.09E-06 | 3.65E-05 | Gm29773 |
| ENSMUSG00000041696 | -2.04362 | 1.09E-06 | 3.66E-05 | Rasl12 |
| ENSMUSG00000021493 | 2.113892 | 1.12E-06 | 3.73E-05 | Pdlim7 |
| ENSMUSG00000027737 | 5.047195 | 1.13E-06 | 3.77E-05 | Slc7a11 |
| ENSMUSG00000025754 | 6.190866 | 1.14E-06 | 3.79E-05 | Agbl1 |
| ENSMUSG00000075217 | -5.5468 | 1.16E-06 | 3.86E-05 | 4833423E24Rik |
| ENSMUSG00000066306 | -0.94007 | 1.20E-06 | 3.99E-05 | Numa1 |
| ENSMUSG00000028364 | 2.62187 | 1.26E-06 | 4.19E-05 | Tnc |
| ENSMUSG00000054510 | 5.29817 | 1.34E-06 | 4.42E-05 | Gm14461 |
| ENSMUSG00000085277 | 4.738024 | 1.35E-06 | 4.47E-05 | Gm13943 |
| ENSMUSG00000001755 | -1.43468 | 1.42E-06 | 4.68E-05 | Coasy |
| ENSMUSG00000068196 | 3.377305 | 1.43E-06 | 4.69E-05 | Col8a1 |
| ENSMUSG00000030148 | 2.024068 | 1.43E-06 | 4.69E-05 | Clec4a2 |
| ENSMUSG00000024039 | -2.64572 | 1.48E-06 | 4.85E-05 | Cbs |
| ENSMUSG00000021136 | -2.46222 | 1.53E-06 | 5.01E-05 | Smoc1 |
| ENSMUSG00000038112 | 2.610391 | 1.56E-06 | 5.09E-05 | AW551984 |
| ENSMUSG00000023064 | -1.48527 | 1.56E-06 | 5.10E-05 | Sncg |
| ENSMUSG00000096146 | 4.350185 | 1.59E-06 | 5.19E-05 | Kcnj11 |
| ENSMUSG00000000435 | 5.90386 | 1.61E-06 | 5.24E-05 | Myf5 |
| ENSMUSG00000113940 | 5.425312 | 1.61E-06 | 5.24E-05 | Gm40477 |
| ENSMUSG00000028116 | 3.877869 | 1.62E-06 | 5.24E-05 | Myoz2 |
| ENSMUSG00000071317 | 3.785069 | 1.63E-06 | 5.26E-05 | Bves |
| ENSMUSG00000029158 | 7.12968 | 1.64E-06 | 5.28E-05 | Yipf7 |
| ENSMUSG00000031198 | 1.242396 | 1.65E-06 | 5.32E-05 | Fundc2 |
| ENSMUSG00000028405 | -1.33772 | 1.66E-06 | 5.34E-05 | Aco1 |
| ENSMUSG00000029380 | 2.780437 | 1.67E-06 | 5.35E-05 | Cxcl1 |
| ENSMUSG00000021033 | -1.96926 | 1.67E-06 | 5.35E-05 | Gstz1 |
| ENSMUSG00000022464 | 3.111447 | 1.69E-06 | 5.42E-05 | Slc38a4 |
| ENSMUSG00000035504 | -2.1466 | 1.70E-06 | 5.42E-05 | Reep6 |
| ENSMUSG00000029778 | -1.81238 | 1.70E-06 | 5.42E-05 | Adcyap1r1 |
| ENSMUSG00000035900 | -1.32998 | 1.70E-06 | 5.42E-05 | Gramd4 |
| ENSMUSG00000106847 | -1.42497 | 1.71E-06 | 5.45E-05 | Peg13 |
| ENSMUSG00000023153 | 3.164591 | 1.71E-06 | 5.45E-05 | Tmem52 |
| ENSMUSG00000034127 | 3.488619 | 1.75E-06 | 5.57E-05 | Tspan8 |
| ENSMUSG00000041957 | -1.6632 | 1.77E-06 | 5.61E-05 | Pkp2 |
| ENSMUSG00000029059 | 1.692458 | 1.77E-06 | 5.61E-05 | Fam213b |
| ENSMUSG00000046070 | -2.03725 | 1.83E-06 | 5.78E-05 | Igfals |
| ENSMUSG00000042828 | 4.487898 | 1.83E-06 | 5.78E-05 | Trim72 |
| ENSMUSG00000016194 | -2.51128 | 1.84E-06 | 5.80E-05 | Hsd11b1 |
| ENSMUSG00000051504 | -2.01834 | 1.85E-06 | 5.82E-05 | Siglech |
| ENSMUSG00000035199 | 0.789925 | 1.87E-06 | 5.85E-05 | Arl6ip5 |
| ENSMUSG00000020190 | -1.22432 | 1.92E-06 | 6.00E-05 | Mknk2 |
| ENSMUSG00000038155 | 1.660305 | 2.03E-06 | 6.34E-05 | Gstp2 |
| ENSMUSG00000034768 | 4.538706 | 2.05E-06 | 6.40E-05 | Asb16 |
| ENSMUSG00000097730 | -3.84651 | 2.14E-06 | 6.66E-05 | Gm26588 |
| ENSMUSG00000036446 | 2.410554 | 2.14E-06 | 6.66E-05 | Lum |
| ENSMUSG00000030319 | 2.428102 | 2.20E-06 | 6.83E-05 | Cand2 |
| ENSMUSG00000025993 | 2.302068 | 2.20E-06 | 6.83E-05 | Slc40a1 |
| ENSMUSG00000029432 | 1.432125 | 2.23E-06 | 6.89E-05 | Nipsnap2 |
| ENSMUSG00000028517 | -1.94253 | 2.27E-06 | 7.01E-05 | Plpp3 |
| ENSMUSG00000025791 | 2.433518 | 2.28E-06 | 7.04E-05 | Pgm2 |
| ENSMUSG00000113739 | 6.772572 | 2.29E-06 | 7.05E-05 | Gm48584 |
| ENSMUSG00000041347 | 2.802281 | 2.32E-06 | 7.15E-05 | Bdkrb1 |
| ENSMUSG00000031672 | 1.458926 | 2.37E-06 | 7.27E-05 | Got2 |
| ENSMUSG00000025161 | 3.022989 | 2.38E-06 | 7.29E-05 | Slc16a3 |
| ENSMUSG00000019970 | 1.227324 | 2.41E-06 | 7.37E-05 | Sgk1 |
| ENSMUSG00000029759 | -1.1345 | 2.43E-06 | 7.42E-05 | Pon3 |
| ENSMUSG00000053117 | 3.905568 | 2.44E-06 | 7.44E-05 | E330013P04Rik |
| ENSMUSG00000085295 | 3.447792 | 2.45E-06 | 7.47E-05 | 4930430E12Rik |
| ENSMUSG00000050541 | -3.53744 | 2.46E-06 | 7.47E-05 | Adra1b |
| ENSMUSG00000021929 | 1.620015 | 2.49E-06 | 7.56E-05 | Kpna3 |
| ENSMUSG00000044461 | 2.737128 | 2.52E-06 | 7.64E-05 | Shisa2 |
| ENSMUSG00000031938 | -1.68043 | 2.59E-06 | 7.85E-05 | 4931406C07Rik |
| ENSMUSG00000025932 | 2.283055 | 2.60E-06 | 7.87E-05 | Eya1 |
| ENSMUSG00000031099 | 1.87607 | 2.64E-06 | 7.97E-05 | Smarca1 |
| ENSMUSG00000000056 | 1.789227 | 2.66E-06 | 8.03E-05 | Narf |
| ENSMUSG00000036879 | 2.168603 | 2.67E-06 | 8.05E-05 | Phkb |
| ENSMUSG00000029752 | -1.87321 | 2.75E-06 | 8.26E-05 | Asns |
| ENSMUSG00000020264 | -3.3163 | 2.86E-06 | 8.59E-05 | Slc36a2 |
| ENSMUSG00000055493 | 1.889514 | 2.88E-06 | 8.62E-05 | Epm2a |
| ENSMUSG00000020916 | -3.38501 | 2.90E-06 | 8.67E-05 | Krt36 |
| ENSMUSG00000064215 | -1.40169 | 2.93E-06 | 8.76E-05 | Ifi27 |
| ENSMUSG00000041737 | -3.07101 | 2.97E-06 | 8.84E-05 | Tmem45b |
| ENSMUSG00000021087 | -2.27877 | 2.98E-06 | 8.87E-05 | Rtn1 |
| ENSMUSG00000057417 | -10.3315 | 3.09E-06 | 9.19E-05 | Dcpp3 |
| ENSMUSG00000024481 | -2.56875 | 3.23E-06 | 9.58E-05 | Lvrn |
| ENSMUSG00000032311 | -2.86892 | 3.24E-06 | 9.58E-05 | Nrg4 |
| ENSMUSG00000038569 | -2.50054 | 3.24E-06 | 9.58E-05 | Rad9b |
| ENSMUSG00000068735 | -1.09342 | 3.26E-06 | 9.64E-05 | Trp53i11 |
| ENSMUSG00000008035 | -1.30763 | 3.35E-06 | 9.87E-05 | Mid1ip1 |
| ENSMUSG00000030278 | -2.72378 | 3.37E-06 | 9.91E-05 | Cidec |
| ENSMUSG00000109904 | 2.78434 | 3.41E-06 | 0.0001 | Gm45819 |
| ENSMUSG00000062929 | 1.711572 | 3.43E-06 | 0.000101 | Cfl2 |
| ENSMUSG00000028417 | 4.813905 | 3.48E-06 | 0.000102 | Tal2 |
| ENSMUSG00000015312 | 1.919971 | 3.54E-06 | 0.000104 | Gadd45b |
| ENSMUSG00000025085 | -1.0042 | 3.68E-06 | 0.000108 | Ablim1 |
| ENSMUSG00000101089 | -2.70255 | 3.71E-06 | 0.000108 | 2610016A17Rik |
| ENSMUSG00000026383 | -1.61644 | 3.75E-06 | 0.000109 | Epb41l5 |
| ENSMUSG00000076549 | -7.91654 | 3.75E-06 | 0.000109 | Igkv4-68 |
| ENSMUSG00000027222 | -1.4782 | 3.76E-06 | 0.000109 | Pex16 |
| ENSMUSG00000025044 | 1.794923 | 3.77E-06 | 0.000109 | Msr1 |
| ENSMUSG00000018846 | -1.42119 | 3.82E-06 | 0.00011 | Pank3 |
| ENSMUSG00000014496 | 1.088358 | 3.82E-06 | 0.00011 | Ankrd28 |
| ENSMUSG00000041417 | -0.86978 | 3.92E-06 | 0.000113 | Pik3r1 |
| ENSMUSG00000021732 | -1.91142 | 3.93E-06 | 0.000113 | Fgf10 |
| ENSMUSG00000066682 | 1.386938 | 3.94E-06 | 0.000114 | Pilrb2 |
| ENSMUSG00000044167 | -1.19369 | 3.98E-06 | 0.000114 | Foxo1 |
| ENSMUSG00000004270 | -1.06125 | 3.99E-06 | 0.000114 | Lpcat3 |
| ENSMUSG00000054034 | 3.034211 | 4.00E-06 | 0.000115 | Tceal5 |
| ENSMUSG00000042712 | 1.210424 | 4.02E-06 | 0.000115 | Tceal9 |
| ENSMUSG00000021996 | 0.958076 | 4.22E-06 | 0.000121 | Esd |
| ENSMUSG00000080850 | 4.827051 | 4.25E-06 | 0.000121 | Gm12439 |
| ENSMUSG00000030137 | 2.543672 | 4.35E-06 | 0.000124 | Tuba8 |
| ENSMUSG00000066621 | -0.9985 | 4.43E-06 | 0.000126 | Tecpr1 |
| ENSMUSG00000059040 | -1.39598 | 4.50E-06 | 0.000128 | Eno1b |
| ENSMUSG00000048772 | -1.3808 | 4.56E-06 | 0.000129 | Tmem53 |
| ENSMUSG00000018920 | 1.378502 | 4.59E-06 | 0.00013 | Cxcl16 |
| ENSMUSG00000023243 | -1.79113 | 4.60E-06 | 0.00013 | Kcnk5 |
| ENSMUSG00000101086 | 6.428282 | 4.62E-06 | 0.00013 | Gm28651 |
| ENSMUSG00000086763 | 1.809456 | 4.68E-06 | 0.000132 | Plxna4os1 |
| ENSMUSG00000070730 | -1.30633 | 4.69E-06 | 0.000132 | Rmdn3 |
| ENSMUSG00000005583 | 1.789278 | 4.69E-06 | 0.000132 | Mef2c |
| ENSMUSG00000040147 | -1.42937 | 4.70E-06 | 0.000132 | Maob |
| ENSMUSG00000091478 | 1.487518 | 4.75E-06 | 0.000133 | Gm10039 |
| ENSMUSG00000037568 | 2.658002 | 4.77E-06 | 0.000133 | Vash2 |
| ENSMUSG00000048126 | 1.560659 | 4.81E-06 | 0.000134 | Col6a3 |
| ENSMUSG00000028278 | 3.078737 | 4.86E-06 | 0.000136 | Rragd |
| ENSMUSG00000068523 | 0.951324 | 4.88E-06 | 0.000136 | Gng5 |
| ENSMUSG00000040972 | -2.85888 | 4.90E-06 | 0.000136 | Igsf21 |
| ENSMUSG00000015733 | 0.958004 | 4.91E-06 | 0.000136 | Capza2 |
| ENSMUSG00000025137 | -1.05321 | 4.96E-06 | 0.000137 | Pcyt2 |
| ENSMUSG00000024397 | 1.347652 | 4.98E-06 | 0.000138 | Aif1 |
| ENSMUSG00000030256 | 2.167498 | 5.06E-06 | 0.00014 | Bhlhe41 |
| ENSMUSG00000039103 | 2.944673 | 5.10E-06 | 0.000141 | Nexn |
| ENSMUSG00000018932 | 1.579821 | 5.12E-06 | 0.000141 | Map2k3 |
| ENSMUSG00000022357 | 2.859472 | 5.14E-06 | 0.000141 | Klhl38 |
| ENSMUSG00000052374 | 3.771921 | 5.19E-06 | 0.000143 | Actn2 |
| ENSMUSG00000020839 | -2.01823 | 5.22E-06 | 0.000143 | Tmigd1 |
| ENSMUSG00000072949 | -1.76868 | 5.24E-06 | 0.000144 | Acot1 |
| ENSMUSG00000028378 | 1.250273 | 5.34E-06 | 0.000146 | Ptgr1 |
| ENSMUSG00000032281 | -2.49888 | 5.46E-06 | 0.000149 | Acsbg1 |
| ENSMUSG00000053214 | -3.89376 | 5.48E-06 | 0.00015 | Gm9899 |
| ENSMUSG00000039620 | -2.01066 | 5.49E-06 | 0.00015 | Trmt9b |
| ENSMUSG00000024526 | -5.9993 | 5.54E-06 | 0.000151 | Cidea |
| ENSMUSG00000112375 | 2.829178 | 5.64E-06 | 0.000153 | Gm38560 |
| ENSMUSG00000039099 | -2.63909 | 5.65E-06 | 0.000153 | Wdr93 |
| ENSMUSG00000060143 | 1.392436 | 5.66E-06 | 0.000153 | Gm10076 |
| ENSMUSG00000048330 | -2.36664 | 5.68E-06 | 0.000154 | Ric3 |
| ENSMUSG00000004730 | 1.484717 | 5.86E-06 | 0.000158 | Adgre1 |
| ENSMUSG00000042529 | 3.234535 | 6.05E-06 | 0.000163 | Kcnj12 |
| ENSMUSG00000035004 | 1.627561 | 6.21E-06 | 0.000167 | Igsf6 |
| ENSMUSG00000019990 | 1.42967 | 6.31E-06 | 0.00017 | Pde7b |
| ENSMUSG00000029722 | -1.2427 | 6.46E-06 | 0.000174 | Agfg2 |
| ENSMUSG00000058818 | 1.272992 | 6.78E-06 | 0.000182 | Pirb |
| ENSMUSG00000047446 | -1.21411 | 7.11E-06 | 0.000191 | Arl4a |
| ENSMUSG00000054428 | 1.295749 | 7.22E-06 | 0.000193 | Atpif1 |
| ENSMUSG00000048965 | -1.42056 | 7.29E-06 | 0.000195 | Mrgpre |
| ENSMUSG00000079434 | 3.336666 | 7.42E-06 | 0.000198 | Neu2 |
| ENSMUSG00000029869 | -2.07392 | 7.43E-06 | 0.000198 | Ephb6 |
| ENSMUSG00000040649 | -4.58132 | 7.49E-06 | 0.000199 | Rimklb |
| ENSMUSG00000000326 | -0.83454 | 7.55E-06 | 0.000201 | Comt |
| ENSMUSG00000027375 | -3.55982 | 7.75E-06 | 0.000206 | Mal |
| ENSMUSG00000031422 | 1.038829 | 7.87E-06 | 0.000209 | Morf4l2 |
| ENSMUSG00000043969 | 3.028957 | 7.88E-06 | 0.000209 | Emx2 |
| ENSMUSG00000050737 | -1.20856 | 8.11E-06 | 0.000214 | Ptges |
| ENSMUSG00000083773 | 2.211158 | 8.13E-06 | 0.000214 | Gm13394 |
| ENSMUSG00000031605 | -1.29524 | 8.13E-06 | 0.000214 | Klhl2 |
| ENSMUSG00000040552 | 1.636825 | 8.17E-06 | 0.000215 | C3ar1 |
| ENSMUSG00000022878 | -2.44609 | 8.31E-06 | 0.000218 | Adipoq |
| ENSMUSG00000038295 | -1.77125 | 8.61E-06 | 0.000226 | Atg9b |
| ENSMUSG00000031209 | -1.44088 | 8.86E-06 | 0.000232 | Heph |
| ENSMUSG00000051727 | -2.12839 | 8.95E-06 | 0.000234 | Kctd14 |
| ENSMUSG00000021474 | -1.21677 | 9.00E-06 | 0.000235 | Sfxn1 |
| ENSMUSG00000022610 | 1.636875 | 9.09E-06 | 0.000237 | Mapk12 |
| ENSMUSG00000043939 | 2.920599 | 9.11E-06 | 0.000238 | A530064D06Rik |
| ENSMUSG00000031400 | -1.2382 | 9.14E-06 | 0.000238 | G6pdx |
| ENSMUSG00000051596 | -4.09689 | 9.19E-06 | 0.000239 | Otop1 |
| ENSMUSG00000026202 | 2.590756 | 9.20E-06 | 0.000239 | Tuba4a |
| ENSMUSG00000056569 | -3.67257 | 9.24E-06 | 0.00024 | Mpz |
| ENSMUSG00000039063 | -2.03561 | 9.48E-06 | 0.000245 | Echdc3 |
| ENSMUSG00000066357 | -1.04967 | 9.49E-06 | 0.000245 | Wdr6 |
| ENSMUSG00000026626 | -1.42229 | 9.63E-06 | 0.000249 | Ppp2r5a |
| ENSMUSG00000038670 | 5.815684 | 9.70E-06 | 0.00025 | Mybpc2 |
| ENSMUSG00000024855 | -0.94513 | 9.70E-06 | 0.00025 | Pacs1 |
| ENSMUSG00000027995 | 1.132509 | 9.71E-06 | 0.00025 | Tlr2 |
| ENSMUSG00000027309 | -1.14164 | 9.75E-06 | 0.00025 | 4930402H24Rik |
| ENSMUSG00000021932 | 1.044038 | 1.01E-05 | 0.000259 | Rnaseh2b |
| ENSMUSG00000025229 | 4.498379 | 1.02E-05 | 0.000261 | Pitx3 |
| ENSMUSG00000023809 | 1.815579 | 1.02E-05 | 0.000261 | Rps6ka2 |
| ENSMUSG00000079227 | 1.64495 | 1.05E-05 | 0.000267 | Ccr5 |
| ENSMUSG00000109237 | -5.30881 | 1.07E-05 | 0.000273 | 9130214F15Rik |
| ENSMUSG00000032503 | 4.550801 | 1.07E-05 | 0.000273 | Arpp21 |
| ENSMUSG00000074813 | 1.585793 | 1.08E-05 | 0.000275 | Gm14005 |
| ENSMUSG00000042082 | -1.11476 | 1.10E-05 | 0.000279 | Arsb |
| ENSMUSG00000025815 | -1.9313 | 1.13E-05 | 0.000288 | Dhtkd1 |
| ENSMUSG00000098814 | -7.81819 | 1.18E-05 | 0.000298 | Igkv19-93 |
| ENSMUSG00000024737 | 1.891753 | 1.18E-05 | 0.000298 | Slc15a3 |
| ENSMUSG00000038086 | 2.560862 | 1.21E-05 | 0.000306 | Hspb2 |
| ENSMUSG00000032666 | 1.322892 | 1.22E-05 | 0.000307 | 1700025G04Rik |
| ENSMUSG00000028691 | 1.27456 | 1.23E-05 | 0.000309 | Prdx1 |
| ENSMUSG00000075555 | -2.36799 | 1.23E-05 | 0.00031 | Gm10863 |
| ENSMUSG00000025375 | -2.47613 | 1.23E-05 | 0.00031 | Aatk |
| ENSMUSG00000020374 | -4.08911 | 1.24E-05 | 0.000312 | Rasgef1c |
| ENSMUSG00000024346 | 0.850516 | 1.25E-05 | 0.000313 | Pfdn1 |
| ENSMUSG00000042029 | 1.209171 | 1.26E-05 | 0.000315 | Ncapg2 |
| ENSMUSG00000002588 | -2.48748 | 1.26E-05 | 0.000316 | Pon1 |
| ENSMUSG00000011257 | 1.53348 | 1.28E-05 | 0.00032 | Pabpc4 |
| ENSMUSG00000090145 | -3.351 | 1.28E-05 | 0.000321 | Ugt1a6b |
| ENSMUSG00000079428 | 4.395974 | 1.29E-05 | 0.000322 | Tceal7 |
| ENSMUSG00000035268 | 1.007625 | 1.30E-05 | 0.000323 | Pkig |
| ENSMUSG00000031963 | 1.679999 | 1.30E-05 | 0.000323 | Bmper |
| ENSMUSG00000034623 | 6.596034 | 1.31E-05 | 0.000324 | Prss55 |
| ENSMUSG00000111204 | 6.446853 | 1.32E-05 | 0.000327 | Gm47320 |
| ENSMUSG00000039914 | 1.670268 | 1.33E-05 | 0.000328 | Coq10a |
| ENSMUSG00000029094 | -0.92748 | 1.35E-05 | 0.000333 | Afap1 |
| ENSMUSG00000076563 | -7.99979 | 1.35E-05 | 0.000334 | Igkv5-48 |
| ENSMUSG00000075330 | 4.525612 | 1.36E-05 | 0.000335 | A930003A15Rik |
| ENSMUSG00000028957 | 1.404804 | 1.36E-05 | 0.000335 | Per3 |
| ENSMUSG00000000686 | -2.46494 | 1.38E-05 | 0.00034 | Abhd15 |
| ENSMUSG00000001247 | -2.45433 | 1.39E-05 | 0.000341 | Lsr |
| ENSMUSG00000069939 | 1.915956 | 1.39E-05 | 0.000341 | Gm12070 |
| ENSMUSG00000038527 | -1.54965 | 1.39E-05 | 0.000341 | C1rl |
| ENSMUSG00000103149 | -2.17093 | 1.40E-05 | 0.000343 | AA914427 |
| ENSMUSG00000104453 | 2.028251 | 1.41E-05 | 0.000344 | Gm37829 |
| ENSMUSG00000047591 | 4.290511 | 1.44E-05 | 0.000351 | Mafa |
| ENSMUSG00000030621 | 1.754274 | 1.45E-05 | 0.000352 | Me3 |
| ENSMUSG00000035545 | -1.04947 | 1.46E-05 | 0.000355 | Leng8 |
| ENSMUSG00000020052 | -6.3135 | 1.46E-05 | 0.000356 | Ascl1 |
| ENSMUSG00000079465 | -4.16652 | 1.48E-05 | 0.000359 | Col4a3 |
| ENSMUSG00000078636 | 1.92357 | 1.49E-05 | 0.000362 | Gm7336 |
| ENSMUSG00000035373 | 2.844847 | 1.49E-05 | 0.000362 | Ccl7 |
| ENSMUSG00000020986 | 1.179495 | 1.50E-05 | 0.000364 | Sec23a |
| ENSMUSG00000046805 | 2.126064 | 1.51E-05 | 0.000365 | Mpeg1 |
| ENSMUSG00000042155 | 1.938911 | 1.51E-05 | 0.000365 | Klhl23 |
| ENSMUSG00000020024 | 0.826398 | 1.52E-05 | 0.000367 | Cep83 |
| ENSMUSG00000019326 | -1.36292 | 1.52E-05 | 0.000367 | Aoc3 |
| ENSMUSG00000035274 | 1.871602 | 1.53E-05 | 0.000368 | Tpbg |
| ENSMUSG00000024193 | -0.9576 | 1.54E-05 | 0.000369 | Phf1 |
| ENSMUSG00000037977 | 3.058273 | 1.54E-05 | 0.000369 | 6430571L13Rik |
| ENSMUSG00000063275 | 2.266626 | 1.54E-05 | 0.000369 | Hacd1 |
| ENSMUSG00000022899 | -2.03768 | 1.54E-05 | 0.00037 | Slc15a2 |
| ENSMUSG00000055214 | 3.481389 | 1.55E-05 | 0.000371 | Pld5 |
| ENSMUSG00000055866 | 1.751821 | 1.56E-05 | 0.000371 | Per2 |
| ENSMUSG00000030317 | -2.4689 | 1.56E-05 | 0.000372 | Timp4 |
| ENSMUSG00000044502 | 1.211523 | 1.56E-05 | 0.000372 | Bod1 |
| ENSMUSG00000033096 | -1.18648 | 1.59E-05 | 0.000378 | Apmap |
| ENSMUSG00000026617 | -0.96156 | 1.60E-05 | 0.00038 | Bpnt1 |
| ENSMUSG00000071001 | -1.36327 | 1.63E-05 | 0.000388 | Hrct1 |
| ENSMUSG00000085069 | -3.71567 | 1.64E-05 | 0.000388 | Gm13111 |
| ENSMUSG00000002763 | -0.98148 | 1.64E-05 | 0.000388 | Pex6 |
| ENSMUSG00000020053 | 1.549322 | 1.68E-05 | 0.000396 | Igf1 |
| ENSMUSG00000024972 | -2.82248 | 1.72E-05 | 0.000407 | Lgals12 |
| ENSMUSG00000105837 | -5.87117 | 1.75E-05 | 0.000413 | Gm35986 |
| ENSMUSG00000022018 | -2.16547 | 1.76E-05 | 0.000415 | Rgcc |
| ENSMUSG00000086679 | -2.54538 | 1.77E-05 | 0.000417 | Gm15551 |
| ENSMUSG00000079471 | 4.68768 | 1.78E-05 | 0.000419 | Mymx |
| ENSMUSG00000022151 | 0.904713 | 1.80E-05 | 0.000422 | Ttc33 |
| ENSMUSG00000030693 | -4.21396 | 1.81E-05 | 0.000424 | Klk10 |
| ENSMUSG00000035963 | 6.86892 | 1.83E-05 | 0.000428 | Odf3l2 |
| ENSMUSG00000022494 | -3.57174 | 1.83E-05 | 0.000428 | Shisa9 |
| ENSMUSG00000063130 | -2.85136 | 1.86E-05 | 0.000433 | Calml3 |
| ENSMUSG00000050471 | 1.094267 | 1.87E-05 | 0.000434 | Fam118b |
| ENSMUSG00000028967 | -1.5649 | 1.87E-05 | 0.000434 | Errfi1 |
| ENSMUSG00000001436 | -1.20311 | 1.87E-05 | 0.000434 | Slc19a1 |
| ENSMUSG00000022225 | -2.38368 | 1.88E-05 | 0.000436 | Cma1 |
| ENSMUSG00000108260 | 6.521716 | 1.89E-05 | 0.000438 | Gm3793 |
| ENSMUSG00000005054 | 1.0213 | 1.91E-05 | 0.000443 | Cstb |
| ENSMUSG00000035226 | -3.87965 | 1.91E-05 | 0.000443 | Rims4 |
| ENSMUSG00000069421 | 6.360931 | 1.93E-05 | 0.000446 | Olfr810 |
| ENSMUSG00000053198 | -3.34774 | 1.98E-05 | 0.000457 | Prx |
| ENSMUSG00000038754 | -3.43617 | 1.99E-05 | 0.000459 | Elovl3 |
| ENSMUSG00000053199 | -1.73351 | 2.03E-05 | 0.000466 | Arhgap20 |
| ENSMUSG00000028031 | 2.033667 | 2.03E-05 | 0.000466 | Dkk2 |
| ENSMUSG00000029919 | 1.423388 | 2.03E-05 | 0.000467 | Hpgds |
| ENSMUSG00000027195 | -1.14826 | 2.04E-05 | 0.000468 | Hsd17b12 |
| ENSMUSG00000028909 | -2.80615 | 2.04E-05 | 0.000468 | Ptpru |
| ENSMUSG00000032323 | -2.74928 | 2.06E-05 | 0.000472 | Cyp11a1 |
| ENSMUSG00000041571 | 1.16008 | 2.10E-05 | 0.000481 | Selenow |
| ENSMUSG00000025736 | -1.1035 | 2.12E-05 | 0.000485 | Jmjd8 |
| ENSMUSG00000062591 | -1.67954 | 2.18E-05 | 0.000496 | Tubb4a |
| ENSMUSG00000022698 | 1.2466 | 2.18E-05 | 0.000496 | Naa50 |
| ENSMUSG00000046828 | 5.420958 | 2.18E-05 | 0.000497 | Mettl21e |
| ENSMUSG00000032028 | -1.43673 | 2.19E-05 | 0.000497 | Nxpe2 |
| ENSMUSG00000050211 | 3.24244 | 2.24E-05 | 0.000508 | Pla2g4e |
| ENSMUSG00000056895 | -2.45681 | 2.24E-05 | 0.000509 | Hist3h2ba |
| ENSMUSG00000039741 | -1.3594 | 2.25E-05 | 0.000509 | Bahcc1 |
| ENSMUSG00000028551 | -1.45768 | 2.25E-05 | 0.000509 | Cdkn2c |
| ENSMUSG00000067242 | -2.66059 | 2.26E-05 | 0.00051 | Lgi1 |
| ENSMUSG00000022066 | 3.068299 | 2.26E-05 | 0.000511 | Entpd4b |
| ENSMUSG00000087029 | -7.51038 | 2.28E-05 | 0.000513 | Gm14133 |
| ENSMUSG00000030189 | 1.834761 | 2.31E-05 | 0.00052 | Ybx3 |
| ENSMUSG00000062082 | 2.097337 | 2.31E-05 | 0.00052 | Cd200r4 |
| ENSMUSG00000055865 | 3.6431 | 2.32E-05 | 0.000522 | Fam19a3 |
| ENSMUSG00000067916 | 1.228666 | 2.34E-05 | 0.000525 | Zfp991 |
| ENSMUSG00000037999 | -2.21333 | 2.35E-05 | 0.000526 | Arap2 |
| ENSMUSG00000027709 | -1.51827 | 2.36E-05 | 0.000528 | Mccc1 |
| ENSMUSG00000030849 | -2.01265 | 2.37E-05 | 0.000529 | Fgfr2 |
| ENSMUSG00000074971 | 2.824075 | 2.38E-05 | 0.000532 | Fibin |
| ENSMUSG00000028475 | -1.64113 | 2.39E-05 | 0.000534 | Spaar |
| ENSMUSG00000017167 | -1.80673 | 2.41E-05 | 0.000536 | Cntnap1 |
| ENSMUSG00000049719 | 6.629916 | 2.50E-05 | 0.000557 | Prss46 |
| ENSMUSG00000117310 | -2.05815 | 2.52E-05 | 0.000559 | Ptp4a1 |
| ENSMUSG00000052852 | 2.099447 | 2.53E-05 | 0.000563 | Reep1 |
| ENSMUSG00000026879 | -1.54956 | 2.55E-05 | 0.000565 | Gsn |
| ENSMUSG00000033200 | -3.84235 | 2.55E-05 | 0.000565 | Tpsg1 |
| ENSMUSG00000025479 | -2.82667 | 2.55E-05 | 0.000565 | Cyp2e1 |
| ENSMUSG00000004187 | -1.4016 | 2.59E-05 | 0.000573 | Kifc2 |
| ENSMUSG00000038543 | 1.415255 | 2.61E-05 | 0.000577 | BC028528 |
| ENSMUSG00000063952 | -0.99321 | 2.62E-05 | 0.000578 | Brpf3 |
| ENSMUSG00000056737 | 1.022421 | 2.64E-05 | 0.000582 | Capg |
| ENSMUSG00000022940 | 0.941221 | 2.71E-05 | 0.000596 | Pigp |
| ENSMUSG00000027560 | 3.267962 | 2.72E-05 | 0.000598 | Dok5 |
| ENSMUSG00000019787 | 5.388379 | 2.77E-05 | 0.000608 | Trdn |
| ENSMUSG00000032294 | 1.51984 | 2.80E-05 | 0.000614 | Pkm |
| ENSMUSG00000010651 | -3.01402 | 2.85E-05 | 0.000625 | Acaa1b |
| ENSMUSG00000026308 | 3.108568 | 2.86E-05 | 0.000626 | Klhl30 |
| ENSMUSG00000031634 | 0.848488 | 2.86E-05 | 0.000626 | Ufsp2 |
| ENSMUSG00000006589 | 0.793049 | 2.89E-05 | 0.000631 | Aprt |
| ENSMUSG00000021131 | 0.811602 | 2.89E-05 | 0.000631 | Erh |
| ENSMUSG00000028273 | 1.469919 | 2.90E-05 | 0.000632 | Pdlim5 |
| ENSMUSG00000109559 | -3.08542 | 2.92E-05 | 0.000634 | Gm34280 |
| ENSMUSG00000099411 | 3.758079 | 2.92E-05 | 0.000634 | 2310015D24Rik |
| ENSMUSG00000008153 | -3.28935 | 2.94E-05 | 0.000638 | Clstn3 |
| ENSMUSG00000019139 | -0.98477 | 2.97E-05 | 0.000644 | Isyna1 |
| ENSMUSG00000002688 | -1.16906 | 3.04E-05 | 0.000658 | Prkd1 |
| ENSMUSG00000090486 | -5.2839 | 3.07E-05 | 0.000665 | BC035947 |
| ENSMUSG00000026255 | -1.65637 | 3.08E-05 | 0.000665 | Efhd1 |
| ENSMUSG00000073600 | 3.085133 | 3.08E-05 | 0.000665 | Prob1 |
| ENSMUSG00000021643 | 1.484761 | 3.10E-05 | 0.000669 | Serf1 |
| ENSMUSG00000048277 | 1.114632 | 3.16E-05 | 0.00068 | Syngr2 |
| ENSMUSG00000045287 | -1.3623 | 3.17E-05 | 0.000682 | Rtn4rl1 |
| ENSMUSG00000031376 | 5.648448 | 3.17E-05 | 0.000682 | Atp2b3 |
| ENSMUSG00000047409 | -0.97882 | 3.18E-05 | 0.000682 | Ctdspl |
| ENSMUSG00000047797 | -3.40686 | 3.20E-05 | 0.000685 | Gjb1 |
| ENSMUSG00000033538 | 0.880351 | 3.24E-05 | 0.000694 | Casp4 |
| ENSMUSG00000061780 | -2.14753 | 3.27E-05 | 0.000699 | Cfd |
| ENSMUSG00000047104 | 7.162524 | 3.28E-05 | 0.000702 | Pbp2 |
| ENSMUSG00000019122 | 1.620712 | 3.30E-05 | 0.000705 | Ccl9 |
| ENSMUSG00000008845 | -1.46258 | 3.32E-05 | 0.000707 | Cd163 |
| ENSMUSG00000058022 | -2.65357 | 3.35E-05 | 0.000713 | Adtrp |
| ENSMUSG00000061462 | 3.313584 | 3.36E-05 | 0.000714 | Obscn |
| ENSMUSG00000035385 | 2.44647 | 3.37E-05 | 0.000716 | Ccl2 |
| ENSMUSG00000042073 | -1.37428 | 3.39E-05 | 0.000719 | Abhd14b |
| ENSMUSG00000072591 | 2.894589 | 3.43E-05 | 0.000728 | 5930412G12Rik |
| ENSMUSG00000022595 | -3.69115 | 3.46E-05 | 0.000733 | Lypd2 |
| ENSMUSG00000020925 | 1.492663 | 3.48E-05 | 0.000737 | Ccdc43 |
| ENSMUSG00000102329 | -2.1804 | 3.49E-05 | 0.000737 | Gm10851 |
| ENSMUSG00000018593 | 1.307278 | 3.49E-05 | 0.000737 | Sparc |
| ENSMUSG00000049521 | -1.20179 | 3.50E-05 | 0.000737 | Cdc42ep1 |
| ENSMUSG00000085014 | 5.664695 | 3.53E-05 | 0.000743 | Gm13490 |
| ENSMUSG00000000223 | -3.64449 | 3.55E-05 | 0.000747 | Drp2 |
| ENSMUSG00000025499 | 1.219795 | 3.58E-05 | 0.000753 | Hras |
| ENSMUSG00000027296 | -3.26785 | 3.63E-05 | 0.000763 | Itpka |
| ENSMUSG00000036186 | -1.45883 | 3.64E-05 | 0.000764 | Fam69b |
| ENSMUSG00000033365 | 1.18322 | 3.65E-05 | 0.000764 | Ipo13 |
| ENSMUSG00000036181 | 0.837856 | 3.65E-05 | 0.000764 | Hist1h1c |
| ENSMUSG00000043683 | 1.822301 | 3.73E-05 | 0.000779 | Fem1a |
| ENSMUSG00000055862 | -1.1359 | 3.76E-05 | 0.000785 | Izumo4 |
| ENSMUSG00000027359 | -3.31314 | 3.78E-05 | 0.000788 | Slc27a2 |
| ENSMUSG00000110755 | -3.26248 | 3.81E-05 | 0.000793 | BC049987 |
| ENSMUSG00000005087 | 1.457649 | 3.82E-05 | 0.000795 | Cd44 |
| ENSMUSG00000024219 | -1.17091 | 3.85E-05 | 0.0008 | Anks1 |
| ENSMUSG00000036390 | 1.46743 | 3.87E-05 | 0.000803 | Gadd45a |
| ENSMUSG00000042515 | 2.074629 | 3.90E-05 | 0.000808 | Mum1l1 |
| ENSMUSG00000031349 | -1.56903 | 3.93E-05 | 0.000815 | Nsdhl |
| ENSMUSG00000076545 | -7.48933 | 4.02E-05 | 0.000831 | Igkv4-72 |
| ENSMUSG00000093930 | -1.47157 | 4.04E-05 | 0.000836 | Hmgcs1 |
| ENSMUSG00000002831 | -2.26746 | 4.07E-05 | 0.000841 | Plin4 |
| ENSMUSG00000039395 | 2.664074 | 4.09E-05 | 0.000843 | Mreg |
| ENSMUSG00000114018 | 5.405663 | 4.10E-05 | 0.000845 | Gm36495 |
| ENSMUSG00000073409 | -1.6034 | 4.11E-05 | 0.000845 | H2-Q6 |
| ENSMUSG00000029994 | 0.996477 | 4.13E-05 | 0.000849 | Anxa4 |
| ENSMUSG00000018169 | -1.64651 | 4.14E-05 | 0.00085 | Mfng |
| ENSMUSG00000060548 | 1.999497 | 4.25E-05 | 0.000872 | Tnfrsf19 |
| ENSMUSG00000048794 | -3.6085 | 4.28E-05 | 0.000877 | Cfap100 |
| ENSMUSG00000031808 | -1.83076 | 4.32E-05 | 0.000885 | Slc27a1 |
| ENSMUSG00000019913 | 6.276226 | 4.33E-05 | 0.000885 | Sim1 |
| ENSMUSG00000035936 | -1.54028 | 4.37E-05 | 0.000892 | Aldh5a1 |
| ENSMUSG00000044468 | -2.52884 | 4.38E-05 | 0.000894 | Tent5c |
| ENSMUSG00000027313 | 2.262848 | 4.44E-05 | 0.000905 | Chac1 |
| ENSMUSG00000005338 | -1.61455 | 4.45E-05 | 0.000906 | Cadm3 |
| ENSMUSG00000087638 | -3.80735 | 4.45E-05 | 0.000906 | Gm11773 |
| ENSMUSG00000028249 | 0.776599 | 4.49E-05 | 0.000913 | Sdcbp |
| ENSMUSG00000021340 | -2.09943 | 4.54E-05 | 0.000921 | Gpld1 |
| ENSMUSG00000038028 | 2.198152 | 4.57E-05 | 0.000927 | Tigar |
| ENSMUSG00000041372 | -5.05316 | 4.64E-05 | 0.00094 | B4galnt3 |
| ENSMUSG00000039428 | -1.04201 | 4.68E-05 | 0.000948 | Tmem135 |
| ENSMUSG00000036078 | -0.87634 | 4.70E-05 | 0.00095 | Sigmar1 |
| ENSMUSG00000039929 | -1.47192 | 4.72E-05 | 0.000954 | Urb1 |
| ENSMUSG00000041058 | 1.179187 | 4.74E-05 | 0.000956 | Wwp1 |
| ENSMUSG00000058056 | 1.573382 | 4.75E-05 | 0.000957 | Palld |
| ENSMUSG00000013629 | -1.01806 | 4.76E-05 | 0.000959 | Cad |
| ENSMUSG00000058715 | 1.359562 | 4.78E-05 | 0.000961 | Fcer1g |
| ENSMUSG00000024866 | -1.37493 | 4.84E-05 | 0.000973 | Acy3 |
| ENSMUSG00000046223 | 1.170271 | 4.87E-05 | 0.000977 | Plaur |
| ENSMUSG00000117222 | 6.312946 | 4.87E-05 | 0.000977 | AC060761.1 |
| ENSMUSG00000024339 | -0.74849 | 4.90E-05 | 0.00098 | Tap2 |
| ENSMUSG00000039886 | -1.3204 | 4.90E-05 | 0.00098 | Tmem120a |
| ENSMUSG00000028496 | 1.599133 | 4.91E-05 | 0.00098 | Mllt3 |
| ENSMUSG00000073987 | -5.19721 | 4.91E-05 | 0.00098 | Ggh |
| ENSMUSG00000042182 | 1.439271 | 4.91E-05 | 0.00098 | Bend6 |
| ENSMUSG00000027335 | -4.2871 | 4.92E-05 | 0.00098 | Adra1d |
| ENSMUSG00000006587 | 3.021071 | 4.93E-05 | 0.000982 | Snai3 |
| ENSMUSG00000030087 | -1.41282 | 4.96E-05 | 0.000986 | Klf15 |
| ENSMUSG00000053063 | 1.774521 | 5.03E-05 | 0.000999 | Clec12a |
| ENSMUSG00000097124 | -2.21763 | 5.06E-05 | 0.001003 | A530020G20Rik |
| ENSMUSG00000035473 | -1.13951 | 5.09E-05 | 0.001009 | Galm |
| ENSMUSG00000026989 | -4.37385 | 5.12E-05 | 0.001014 | Dapl1 |
| ENSMUSG00000091575 | -3.85609 | 5.13E-05 | 0.001015 | 2010016I18Rik |
| ENSMUSG00000107066 | -2.39988 | 5.14E-05 | 0.001015 | Gm4482 |
| ENSMUSG00000102059 | -1.67541 | 5.14E-05 | 0.001015 | Gm20257 |
| ENSMUSG00000029456 | -1.39201 | 5.24E-05 | 0.001034 | Acad10 |
| ENSMUSG00000060550 | -2.05434 | 5.25E-05 | 0.001034 | H2-Q7 |
| ENSMUSG00000021416 | -2.29466 | 5.30E-05 | 0.001043 | Eci3 |
| ENSMUSG00000030662 | 0.89115 | 5.38E-05 | 0.001056 | Ipo5 |
| ENSMUSG00000047528 | -2.41423 | 5.38E-05 | 0.001056 | Als2cr12 |
| ENSMUSG00000097317 | 3.769102 | 5.41E-05 | 0.001061 | Gm17281 |
| ENSMUSG00000031443 | 3.575662 | 5.42E-05 | 0.001062 | F7 |
| ENSMUSG00000096596 | 2.034979 | 5.51E-05 | 0.001077 | Gm10591 |
| ENSMUSG00000028841 | 3.348346 | 5.51E-05 | 0.001077 | Cnksr1 |
| ENSMUSG00000078532 | 2.007938 | 5.51E-05 | 0.001077 | Nkain1 |
| ENSMUSG00000040652 | 1.336309 | 5.53E-05 | 0.001079 | Oaz2 |
| ENSMUSG00000040105 | -1.21511 | 5.54E-05 | 0.00108 | Plpp6 |
| ENSMUSG00000014776 | 1.636137 | 5.55E-05 | 0.001082 | Nol3 |
| ENSMUSG00000075224 | 5.664028 | 5.57E-05 | 0.001085 | Lrrc55 |
| ENSMUSG00000028029 | 0.817383 | 5.58E-05 | 0.001085 | Aimp1 |
| ENSMUSG00000044199 | -3.24535 | 5.65E-05 | 0.001097 | S1pr4 |
| ENSMUSG00000002769 | -3.02553 | 5.65E-05 | 0.001097 | Gnmt |
| ENSMUSG00000033059 | -0.94709 | 5.67E-05 | 0.001099 | Pygb |
| ENSMUSG00000010461 | 2.25664 | 5.74E-05 | 0.00111 | Eya4 |
| ENSMUSG00000025911 | -1.53714 | 5.74E-05 | 0.00111 | Adhfe1 |
| ENSMUSG00000096956 | 0.897926 | 5.76E-05 | 0.001113 | Snhg18 |
| ENSMUSG00000022040 | -1.882 | 5.77E-05 | 0.001114 | Ephx2 |
| ENSMUSG00000024049 | 3.022915 | 5.90E-05 | 0.001137 | Myom1 |
| ENSMUSG00000055675 | -1.59595 | 5.93E-05 | 0.001142 | Kbtbd11 |
| ENSMUSG00000051412 | 0.851005 | 5.97E-05 | 0.001149 | Vamp7 |
| ENSMUSG00000005672 | -1.48163 | 5.99E-05 | 0.001151 | Kit |
| ENSMUSG00000024033 | 2.347815 | 6.01E-05 | 0.001155 | Rsph1 |
| ENSMUSG00000042010 | -1.8314 | 6.04E-05 | 0.001158 | Acacb |
| ENSMUSG00000019528 | 1.513286 | 6.10E-05 | 0.001168 | Gyg |
| ENSMUSG00000026051 | 2.38465 | 6.14E-05 | 0.001175 | 1500015O10Rik |
| ENSMUSG00000063382 | -1.00068 | 6.16E-05 | 0.001178 | Bcl9l |
| ENSMUSG00000060904 | 0.80849 | 6.16E-05 | 0.001178 | Arl1 |
| ENSMUSG00000106755 | 2.141593 | 6.19E-05 | 0.001182 | Tpi-rs11 |
| ENSMUSG00000042644 | -1.38499 | 6.36E-05 | 0.001212 | Itpr3 |
| ENSMUSG00000021061 | 1.895246 | 6.52E-05 | 0.001243 | Sptb |
| ENSMUSG00000039205 | -0.8129 | 6.55E-05 | 0.001246 | Ciz1 |
| ENSMUSG00000085965 | 4.234184 | 6.58E-05 | 0.001251 | 2310016D23Rik |
| ENSMUSG00000096326 | -6.34 | 6.71E-05 | 0.001274 | Ighv1-78 |
| ENSMUSG00000083307 | 1.833656 | 6.75E-05 | 0.001281 | AA414768 |
| ENSMUSG00000042607 | 2.385257 | 6.80E-05 | 0.00129 | Asb4 |
| ENSMUSG00000031725 | -2.50733 | 6.82E-05 | 0.001292 | Ces1f |
| ENSMUSG00000031147 | 1.678426 | 6.83E-05 | 0.001293 | Magix |
| ENSMUSG00000067616 | -5.01026 | 6.89E-05 | 0.001301 | Klk11 |
| ENSMUSG00000070547 | -2.16442 | 6.89E-05 | 0.001301 | Mrgprb1 |
| ENSMUSG00000035916 | 5.328284 | 6.89E-05 | 0.001301 | Ptprq |
| ENSMUSG00000029128 | 0.777937 | 6.96E-05 | 0.001312 | Rab28 |
| ENSMUSG00000034460 | 2.271894 | 6.97E-05 | 0.001312 | Six4 |
| ENSMUSG00000029596 | -2.19178 | 7.02E-05 | 0.001321 | Sdsl |
| ENSMUSG00000044986 | -1.8466 | 7.07E-05 | 0.001328 | Tst |
| ENSMUSG00000085071 | -2.73183 | 7.16E-05 | 0.001344 | Gm14066 |
| ENSMUSG00000049349 | 6.303887 | 7.19E-05 | 0.001347 | Gm5105 |
| ENSMUSG00000074121 | 3.053956 | 7.19E-05 | 0.001347 | Ntf5 |
| ENSMUSG00000030086 | -1.07388 | 7.22E-05 | 0.001352 | Chchd6 |
| ENSMUSG00000029392 | 1.895913 | 7.31E-05 | 0.001367 | Rilpl1 |
| ENSMUSG00000025856 | 1.236071 | 7.39E-05 | 0.00138 | Pdgfa |
| ENSMUSG00000031523 | -1.03949 | 7.44E-05 | 0.001388 | Dlc1 |
| ENSMUSG00000056313 | -1.37667 | 7.45E-05 | 0.001388 | Tcim |
| ENSMUSG00000019082 | -1.15787 | 7.54E-05 | 0.001403 | Slc25a22 |
| ENSMUSG00000061740 | -1.61698 | 7.55E-05 | 0.001403 | Cyp2d22 |
| ENSMUSG00000020744 | -1.54118 | 7.55E-05 | 0.001403 | Slc25a19 |
| ENSMUSG00000036395 | -2.37712 | 7.73E-05 | 0.001435 | Glb1l2 |
| ENSMUSG00000050335 | 2.15215 | 7.74E-05 | 0.001435 | Lgals3 |
| ENSMUSG00000112023 | 2.131766 | 7.84E-05 | 0.001454 | Lilr4b |
| ENSMUSG00000050967 | 3.618364 | 7.96E-05 | 0.001473 | Creg2 |
| ENSMUSG00000054619 | -1.0717 | 8.24E-05 | 0.001525 | Mettl7a1 |
| ENSMUSG00000050777 | 1.094537 | 8.33E-05 | 0.001539 | Tmem37 |
| ENSMUSG00000033777 | 2.139405 | 8.35E-05 | 0.001543 | Tlr13 |
| ENSMUSG00000038721 | 1.980393 | 8.38E-05 | 0.001546 | Hoxb7 |
| ENSMUSG00000069633 | -1.24384 | 8.39E-05 | 0.001546 | Pex11g |
| ENSMUSG00000023951 | -1.20611 | 8.43E-05 | 0.001552 | Vegfa |
| ENSMUSG00000030089 | 1.768329 | 8.47E-05 | 0.001559 | Slc41a3 |
| ENSMUSG00000023328 | 2.270461 | 8.52E-05 | 0.001565 | Ache |
| ENSMUSG00000021737 | 1.034028 | 8.53E-05 | 0.001565 | Psmd6 |
| ENSMUSG00000028838 | 2.373792 | 8.54E-05 | 0.001565 | Extl1 |
| ENSMUSG00000024426 | -1.18968 | 8.55E-05 | 0.001566 | Atat1 |
| ENSMUSG00000041423 | -2.92119 | 8.57E-05 | 0.001567 | Paqr6 |
| ENSMUSG00000058258 | -1.35709 | 8.57E-05 | 0.001567 | Idi1 |
| ENSMUSG00000030124 | -2.07744 | 8.59E-05 | 0.001569 | Lag3 |
| ENSMUSG00000026480 | 1.322328 | 8.68E-05 | 0.001585 | Ncf2 |
| ENSMUSG00000025246 | -0.78953 | 8.71E-05 | 0.001588 | Tbl1x |
| ENSMUSG00000042359 | 1.878638 | 8.73E-05 | 0.001591 | Osbpl6 |
| ENSMUSG00000005373 | -1.99118 | 8.83E-05 | 0.001606 | Mlxipl |
| ENSMUSG00000060063 | 1.292945 | 8.83E-05 | 0.001606 | Alox5ap |
| ENSMUSG00000115924 | -5.37206 | 8.96E-05 | 0.001628 | Gm19277 |
| ENSMUSG00000032527 | -1.32657 | 9.00E-05 | 0.001633 | Pccb |
| ENSMUSG00000025351 | 1.122477 | 9.13E-05 | 0.001655 | Cd63 |
| ENSMUSG00000026971 | 3.543497 | 9.14E-05 | 0.001655 | Itgb6 |
| ENSMUSG00000030789 | 3.12159 | 9.16E-05 | 0.001657 | Itgax |
| ENSMUSG00000113010 | 1.655891 | 9.26E-05 | 0.001675 | Gm34084 |
| ENSMUSG00000051439 | 1.344443 | 9.27E-05 | 0.001675 | Cd14 |
| ENSMUSG00000022840 | -1.83851 | 9.38E-05 | 0.001692 | Adcy5 |
| ENSMUSG00000073411 | -0.79603 | 9.40E-05 | 0.001695 | H2-D1 |
| ENSMUSG00000102824 | 1.027893 | 9.47E-05 | 0.001706 | Pdcd5-ps |
| ENSMUSG00000110453 | 4.065633 | 9.56E-05 | 0.001718 | Gm32352 |
| ENSMUSG00000075590 | -1.79621 | 9.56E-05 | 0.001718 | Nrbp2 |
| ENSMUSG00000021281 | 1.475837 | 9.65E-05 | 0.001733 | Tnfaip2 |
| ENSMUSG00000030111 | -3.77032 | 9.81E-05 | 0.00176 | A2m |
| ENSMUSG00000001027 | 3.409301 | 9.90E-05 | 0.001775 | Scn4a |
| ENSMUSG00000022389 | 1.380269 | 9.94E-05 | 0.00178 | Tef |
| ENSMUSG00000099364 | 4.605371 | 9.96E-05 | 0.001782 | 5730419F03Rik |
| ENSMUSG00000002341 | -5.11877 | 9.98E-05 | 0.001783 | Ncan |
| ENSMUSG00000045252 | -1.02137 | 9.98E-05 | 0.001783 | Zfp574 |
| ENSMUSG00000116498 | -4.45508 | 0.000101 | 0.001795 | Gm38563 |
| ENSMUSG00000019823 | -0.89008 | 0.000101 | 0.001795 | Mical1 |
| ENSMUSG00000046323 | 3.607581 | 0.000101 | 0.001802 | Dppa3 |
| ENSMUSG00000018796 | -1.94066 | 0.000102 | 0.001806 | Acsl1 |
| ENSMUSG00000028033 | 2.591412 | 0.000102 | 0.001817 | Kcnq5 |
| ENSMUSG00000043924 | -4.4923 | 0.000103 | 0.001824 | Ncmap |
| ENSMUSG00000020547 | 1.315486 | 0.000103 | 0.001827 | Bzw2 |
| ENSMUSG00000040613 | 1.175167 | 0.000104 | 0.001846 | Apobec1 |
| ENSMUSG00000032898 | -1.13026 | 0.000105 | 0.001857 | Fbxo21 |
| ENSMUSG00000000386 | 2.539577 | 0.000105 | 0.001857 | Mx1 |
| ENSMUSG00000061877 | 6.545815 | 0.000105 | 0.001859 | BC048679 |
| ENSMUSG00000054196 | 2.872366 | 0.000105 | 0.001861 | Cthrc1 |
| ENSMUSG00000018841 | -1.02425 | 0.000106 | 0.001866 | Rad51d |
| ENSMUSG00000028248 | -1.03501 | 0.000106 | 0.001871 | Pnisr |
| ENSMUSG00000110439 | 4.211077 | 0.000107 | 0.001881 | Gm21320 |
| ENSMUSG00000041482 | 2.13019 | 0.000107 | 0.001885 | Piezo2 |
| ENSMUSG00000052373 | 2.993264 | 0.000107 | 0.001885 | Mpp3 |
| ENSMUSG00000047747 | 1.177407 | 0.000108 | 0.001891 | Rnf150 |
| ENSMUSG00000051000 | 1.872298 | 0.000109 | 0.001912 | Fam160a1 |
| ENSMUSG00000031461 | 5.583423 | 0.00011 | 0.001922 | Myom2 |
| ENSMUSG00000062729 | -0.75062 | 0.00011 | 0.001923 | Ppox |
| ENSMUSG00000036894 | 1.194958 | 0.00011 | 0.001925 | Rap2b |
| ENSMUSG00000021936 | 0.828513 | 0.000111 | 0.001936 | Mapk8 |
| ENSMUSG00000062209 | -4.50846 | 0.000111 | 0.001945 | Erbb4 |
| ENSMUSG00000028792 | -1.13806 | 0.000111 | 0.001946 | Ak2 |
| ENSMUSG00000093805 | -6.61269 | 0.000112 | 0.001948 | Gal3st2b |
| ENSMUSG00000029915 | 1.5717 | 0.000112 | 0.001952 | Clec5a |
| ENSMUSG00000029123 | -2.37546 | 0.000112 | 0.001952 | Stk32b |
| ENSMUSG00000090942 | 1.931622 | 0.000113 | 0.00197 | F830016B08Rik |
| ENSMUSG00000031636 | 2.092239 | 0.000114 | 0.001977 | Pdlim3 |
| ENSMUSG00000028736 | 5.711765 | 0.000115 | 0.001998 | Pax7 |
| ENSMUSG00000041911 | -6.09341 | 0.000115 | 0.002003 | Dlx1 |
| ENSMUSG00000049641 | 4.059203 | 0.000116 | 0.002007 | Vgll2 |
| ENSMUSG00000040181 | -1.33277 | 0.000116 | 0.002012 | Fmo1 |
| ENSMUSG00000045441 | -2.32501 | 0.000116 | 0.002012 | Gprin3 |
| ENSMUSG00000034353 | 1.339294 | 0.000117 | 0.002021 | Ramp1 |
| ENSMUSG00000020865 | 1.385987 | 0.000117 | 0.002021 | Abcc3 |
| ENSMUSG00000002064 | 0.803675 | 0.000118 | 0.002027 | Sdf2 |
| ENSMUSG00000051098 | -1.31163 | 0.000118 | 0.002032 | Mblac2 |
| ENSMUSG00000031799 | 1.126405 | 0.000118 | 0.002034 | Tpm4 |
| ENSMUSG00000032827 | -1.59503 | 0.000118 | 0.002034 | Ppp1r9a |
| ENSMUSG00000102918 | -1.57234 | 0.000119 | 0.002037 | Pcdhgc3 |
| ENSMUSG00000029772 | -1.19421 | 0.000119 | 0.00204 | Ahcyl2 |
| ENSMUSG00000029373 | 1.182453 | 0.00012 | 0.002062 | Pf4 |
| ENSMUSG00000047592 | 2.044036 | 0.00012 | 0.002062 | Nxpe5 |
| ENSMUSG00000019820 | -1.17728 | 0.00012 | 0.002062 | Utrn |
| ENSMUSG00000097617 | -1.65274 | 0.000121 | 0.002066 | Gm10687 |
| ENSMUSG00000055737 | -1.22842 | 0.000122 | 0.002078 | Ghr |
| ENSMUSG00000028518 | 1.644372 | 0.000123 | 0.002094 | Prkaa2 |
| ENSMUSG00000031428 | -2.7528 | 0.000123 | 0.002107 | Zcchc18 |
| ENSMUSG00000055322 | -1.14835 | 0.000124 | 0.002112 | Tns1 |
| ENSMUSG00000030730 | 5.049557 | 0.000125 | 0.002123 | Atp2a1 |
| ENSMUSG00000090000 | 0.810183 | 0.000125 | 0.002123 | Ier3ip1 |
| ENSMUSG00000020778 | 1.045104 | 0.000125 | 0.002123 | Ten1 |
| ENSMUSG00000027236 | 1.01979 | 0.000128 | 0.002175 | Eif3j1 |
| ENSMUSG00000063388 | 2.690426 | 0.000129 | 0.002188 | BC023105 |
| ENSMUSG00000073968 | -0.91122 | 0.000129 | 0.002189 | Trim68 |
| ENSMUSG00000048371 | -1.54237 | 0.000129 | 0.002189 | Pdp2 |
| ENSMUSG00000028955 | 0.661328 | 0.000129 | 0.002189 | Vamp3 |
| ENSMUSG00000030126 | 1.022966 | 0.000132 | 0.002224 | Tmcc1 |
| ENSMUSG00000021190 | 1.174861 | 0.000132 | 0.002224 | Lgmn |
| ENSMUSG00000027489 | -2.13373 | 0.000132 | 0.002232 | Necab3 |
| ENSMUSG00000026502 | 0.82638 | 0.000134 | 0.002263 | Desi2 |
| ENSMUSG00000031066 | -1.21234 | 0.000134 | 0.002266 | Usp11 |
| ENSMUSG00000050628 | 0.791784 | 0.000135 | 0.002266 | Ubald2 |
| ENSMUSG00000007030 | 2.509722 | 0.000136 | 0.00229 | Vwa7 |
| ENSMUSG00000003282 | -2.14225 | 0.000137 | 0.002295 | Plag1 |
| ENSMUSG00000056515 | 1.266024 | 0.000139 | 0.002328 | Rab31 |
| ENSMUSG00000026874 | 4.945953 | 0.000139 | 0.002335 | Hc |
| ENSMUSG00000044349 | -2.47643 | 0.000139 | 0.002336 | Snhg11 |
| ENSMUSG00000064120 | -1.11311 | 0.000141 | 0.002358 | Mocs1 |
| ENSMUSG00000020743 | -1.01935 | 0.000143 | 0.002393 | Mif4gd |
| ENSMUSG00000112246 | -5.60227 | 0.000143 | 0.002396 | Gm40761 |
| ENSMUSG00000027333 | 2.996222 | 0.000144 | 0.00241 | Smox |
| ENSMUSG00000034957 | -1.59161 | 0.000145 | 0.002419 | Cebpa |
| ENSMUSG00000043833 | -1.60592 | 0.000145 | 0.002419 | 2900005J15Rik |
| ENSMUSG00000026656 | 1.284944 | 0.000145 | 0.00242 | Fcgr2b |
| ENSMUSG00000039067 | 0.780317 | 0.000146 | 0.002428 | Psmd7 |
| ENSMUSG00000075027 | -3.48229 | 0.000147 | 0.002443 | 4631405J19Rik |
| ENSMUSG00000031490 | -0.83827 | 0.000147 | 0.002443 | Eif4ebp1 |
| ENSMUSG00000023885 | 1.787193 | 0.000147 | 0.002443 | Thbs2 |
| ENSMUSG00000025648 | 1.195853 | 0.000148 | 0.002455 | Pfkfb4 |
| ENSMUSG00000085939 | 1.006981 | 0.000148 | 0.002455 | Cd63-ps |
| ENSMUSG00000094707 | 2.259331 | 0.000151 | 0.002493 | A830019P07Rik |
| ENSMUSG00000076672 | -5.00748 | 0.000151 | 0.002493 | Ighv3-6 |
| ENSMUSG00000086448 | -2.30151 | 0.000151 | 0.002501 | 9330162012Rik |
| ENSMUSG00000024867 | -3.08064 | 0.000152 | 0.002513 | Pip5k1b |
| ENSMUSG00000049643 | 1.382343 | 0.000152 | 0.002517 | 2310022A10Rik |
| ENSMUSG00000075232 | 2.604933 | 0.000153 | 0.002518 | Amd1 |
| ENSMUSG00000004085 | 1.694073 | 0.000153 | 0.00252 | Map3k20 |
| ENSMUSG00000040782 | 0.871659 | 0.000153 | 0.00252 | Cop1 |
| ENSMUSG00000074486 | -6.31234 | 0.000153 | 0.002521 | Bglap2 |
| ENSMUSG00000110344 | -1.75995 | 0.000154 | 0.002533 | Gm45716 |
| ENSMUSG00000020307 | 1.280394 | 0.000155 | 0.00255 | Cdc34 |
| ENSMUSG00000032064 | -1.4316 | 0.000155 | 0.002551 | Dixdc1 |
| ENSMUSG00000023764 | -0.9195 | 0.000156 | 0.002564 | Sfi1 |
| ENSMUSG00000099974 | 2.161565 | 0.000157 | 0.002564 | Bcl2a1d |
| ENSMUSG00000031253 | 1.700777 | 0.000157 | 0.00257 | Srpx2 |
| ENSMUSG00000031536 | 0.891276 | 0.000157 | 0.002573 | Polb |
| ENSMUSG00000115414 | 3.535176 | 0.000157 | 0.002573 | Gm49257 |
| ENSMUSG00000003119 | -0.81615 | 0.000159 | 0.002597 | Cdk12 |
| ENSMUSG00000052949 | 1.503245 | 0.00016 | 0.002605 | Rnf157 |
| ENSMUSG00000056880 | 3.861581 | 0.000161 | 0.002617 | Gadl1 |
| ENSMUSG00000027852 | 0.745056 | 0.000161 | 0.002617 | Nras |
| ENSMUSG00000035297 | 0.86755 | 0.000161 | 0.002618 | Cops4 |
| ENSMUSG00000026042 | 1.530339 | 0.000162 | 0.002629 | Col5a2 |
| ENSMUSG00000049940 | -0.85454 | 0.000162 | 0.002631 | Pgrmc2 |
| ENSMUSG00000058013 | 0.99653 | 0.000163 | 0.002647 | 11-Sep |
| ENSMUSG00000046079 | -0.93233 | 0.000163 | 0.002649 | Lrrc8d |
| ENSMUSG00000027346 | 1.468433 | 0.000164 | 0.002649 | Gpcpd1 |
| ENSMUSG00000027901 | -2.67311 | 0.000164 | 0.002655 | Dennd2d |
| ENSMUSG00000051786 | -0.93869 | 0.000164 | 0.002657 | Tubgcp6 |
| ENSMUSG00000110604 | -6.80965 | 0.000166 | 0.002675 | Gm45824 |
| ENSMUSG00000030495 | -2.08707 | 0.000167 | 0.002695 | Slc7a10 |
| ENSMUSG00000029406 | -1.41696 | 0.000167 | 0.002702 | Pitpnm2 |
| ENSMUSG00000111085 | 4.028602 | 0.000168 | 0.002702 | Gm7607 |
| ENSMUSG00000037960 | 0.825688 | 0.000168 | 0.002703 | Card19 |
| ENSMUSG00000058427 | 3.901947 | 0.000168 | 0.002703 | Cxcl2 |
| ENSMUSG00000085888 | 6.135536 | 0.000169 | 0.002711 | Gm12224 |
| ENSMUSG00000086727 | -4.01316 | 0.00017 | 0.002737 | 4931428L18Rik |
| ENSMUSG00000032717 | 1.721281 | 0.000171 | 0.002744 | Mdfi |
| ENSMUSG00000078771 | 1.371451 | 0.000172 | 0.002762 | Evi2a |
| ENSMUSG00000015291 | -0.83924 | 0.000173 | 0.002769 | Gdi1 |
| ENSMUSG00000038506 | 1.295208 | 0.000173 | 0.00277 | Dcun1d2 |
| ENSMUSG00000037353 | -1.62519 | 0.000173 | 0.002775 | Letmd1 |
| ENSMUSG00000048240 | -1.52042 | 0.000174 | 0.002787 | Gng7 |
| ENSMUSG00000059422 | 1.525092 | 0.000175 | 0.002789 | Gm8116 |
| ENSMUSG00000031962 | 4.431651 | 0.000175 | 0.002789 | Cdh15 |
| ENSMUSG00000045102 | -3.03878 | 0.000183 | 0.002916 | Poln |
| ENSMUSG00000079056 | -1.84044 | 0.000184 | 0.002939 | Kcnip3 |
| ENSMUSG00000048175 | 1.32314 | 0.000185 | 0.002939 | Asb8 |
| ENSMUSG00000035357 | 1.332027 | 0.000185 | 0.002939 | Pdzrn3 |
| ENSMUSG00000043740 | 2.329052 | 0.000187 | 0.002966 | B430306N03Rik |
| ENSMUSG00000001497 | -2.96686 | 0.000187 | 0.002967 | Pax9 |
| ENSMUSG00000015652 | 1.999409 | 0.000187 | 0.002969 | Steap1 |
| ENSMUSG00000060147 | 1.156983 | 0.000188 | 0.002983 | Serpinb6a |
| ENSMUSG00000001865 | -2.10914 | 0.000189 | 0.002994 | Cpa3 |
| ENSMUSG00000008540 | -1.08504 | 0.000189 | 0.002999 | Mgst1 |
| ENSMUSG00000005057 | -1.75217 | 0.00019 | 0.003008 | Sh2b2 |
| ENSMUSG00000028479 | -0.93278 | 0.00019 | 0.003009 | Gne |
| ENSMUSG00000000581 | 0.797683 | 0.000191 | 0.003013 | C1d |
| ENSMUSG00000036169 | -2.79603 | 0.000192 | 0.003029 | Sostdc1 |
| ENSMUSG00000109130 | -1.81714 | 0.000193 | 0.003037 | Gm45187 |
| ENSMUSG00000020061 | 5.089836 | 0.000197 | 0.003098 | Mybpc1 |
| ENSMUSG00000063229 | 1.375727 | 0.000198 | 0.003109 | Ldha |
| ENSMUSG00000022994 | -1.06175 | 0.000199 | 0.00313 | Adcy6 |
| ENSMUSG00000021922 | -3.11175 | 0.000201 | 0.003161 | Itih4 |
| ENSMUSG00000110545 | 0.955851 | 0.000203 | 0.003187 | Gm7730 |
| ENSMUSG00000043719 | 1.603782 | 0.000204 | 0.003202 | Col6a6 |
| ENSMUSG00000001891 | 1.277422 | 0.000205 | 0.003207 | Ugp2 |
| ENSMUSG00000031885 | 1.131465 | 0.000205 | 0.003207 | Cbfb |
| ENSMUSG00000048138 | -1.57827 | 0.000205 | 0.003213 | Dmrt2 |
| ENSMUSG00000049176 | 2.853646 | 0.000206 | 0.003218 | Frmpd4 |
| ENSMUSG00000061313 | -1.02308 | 0.000207 | 0.003238 | Ddhd2 |
| ENSMUSG00000042190 | -1.02823 | 0.000207 | 0.003238 | Cmklr1 |
| ENSMUSG00000110539 | -5.60347 | 0.000208 | 0.003243 | Gm45864 |
| ENSMUSG00000028212 | 1.508981 | 0.00021 | 0.003269 | Ccne2 |
| ENSMUSG00000006360 | 1.244383 | 0.000211 | 0.00328 | Crip1 |
| ENSMUSG00000020363 | -1.49121 | 0.000212 | 0.003291 | Gfpt2 |
| ENSMUSG00000024759 | -0.7523 | 0.000213 | 0.003314 | Atl3 |
| ENSMUSG00000051579 | 1.12163 | 0.000218 | 0.003379 | Tceal8 |
| ENSMUSG00000029925 | 1.079942 | 0.00022 | 0.003421 | Tbxas1 |
| ENSMUSG00000061232 | -1.08726 | 0.000222 | 0.003438 | H2-K1 |
| ENSMUSG00000037316 | -0.96166 | 0.000222 | 0.003438 | Bag4 |
| ENSMUSG00000035923 | 5.599426 | 0.000222 | 0.003438 | Myf6 |
| ENSMUSG00000028788 | 0.949018 | 0.000222 | 0.003441 | Ptp4a2 |
| ENSMUSG00000051185 | 0.987353 | 0.000223 | 0.003447 | Fam174a |
| ENSMUSG00000017737 | -1.32335 | 0.000224 | 0.00346 | Mmp9 |
| ENSMUSG00000036745 | 3.050717 | 0.000224 | 0.00346 | Ttll7 |
| ENSMUSG00000027832 | 1.903627 | 0.000224 | 0.00346 | Ptx3 |
| ENSMUSG00000026456 | 1.056375 | 0.000227 | 0.0035 | Cyb5r1 |
| ENSMUSG00000027206 | 0.728298 | 0.000229 | 0.003529 | Cops2 |
| ENSMUSG00000026981 | 2.941892 | 0.00023 | 0.003534 | Il1rn |
| ENSMUSG00000030560 | 1.044218 | 0.00023 | 0.003534 | Ctsc |
| ENSMUSG00000035929 | -0.79831 | 0.00023 | 0.003538 | H2-Q4 |
| ENSMUSG00000022707 | -1.4264 | 0.000232 | 0.003554 | Gbe1 |
| ENSMUSG00000030664 | -2.20568 | 0.000232 | 0.003554 | Sox6os |
| ENSMUSG00000063558 | -1.20759 | 0.000232 | 0.00356 | Aox1 |
| ENSMUSG00000070605 | 1.230005 | 0.000234 | 0.00358 | Zfp992 |
| ENSMUSG00000025511 | 1.020788 | 0.000237 | 0.003621 | Tspan4 |
| ENSMUSG00000030244 | -3.63789 | 0.000238 | 0.003634 | Gys2 |
| ENSMUSG00000024169 | -0.90451 | 0.000238 | 0.003637 | Ift140 |
| ENSMUSG00000025909 | -3.14779 | 0.000238 | 0.003639 | Sntg1 |
| ENSMUSG00000016552 | -0.94165 | 0.00024 | 0.003657 | Foxred2 |
| ENSMUSG00000019039 | -0.87071 | 0.00024 | 0.003661 | Dalrd3 |
| ENSMUSG00000038205 | 1.703064 | 0.000243 | 0.003704 | Prkab2 |
| ENSMUSG00000048040 | -2.18592 | 0.000244 | 0.003707 | Arxes2 |
| ENSMUSG00000030671 | -1.70793 | 0.000245 | 0.003716 | Pde3b |
| ENSMUSG00000073375 | 5.464153 | 0.000245 | 0.003716 | Lrrc30 |
| ENSMUSG00000005609 | -0.81679 | 0.000246 | 0.003724 | Ctr9 |
| ENSMUSG00000036825 | 0.95805 | 0.000246 | 0.003724 | Ssx2ip |
| ENSMUSG00000029272 | -3.20596 | 0.000246 | 0.003731 | Sult1e1 |
| ENSMUSG00000031283 | -2.07568 | 0.000247 | 0.003737 | Chrdl1 |
| ENSMUSG00000039865 | -1.99697 | 0.000247 | 0.00374 | Slc44a3 |
| ENSMUSG00000011305 | -2.51169 | 0.000248 | 0.003742 | Plin5 |
| ENSMUSG00000061878 | 1.490891 | 0.000248 | 0.003752 | Sphk1 |
| ENSMUSG00000027288 | 1.047812 | 0.000253 | 0.003814 | Zfp106 |
| ENSMUSG00000058297 | -2.75033 | 0.000253 | 0.003815 | Spock2 |
| ENSMUSG00000028943 | -1.43525 | 0.000253 | 0.003817 | Espn |
| ENSMUSG00000062901 | 0.78289 | 0.000255 | 0.003837 | Klhl24 |
| ENSMUSG00000020902 | -1.97185 | 0.000256 | 0.00385 | Ntn1 |
| ENSMUSG00000029016 | -0.89404 | 0.00026 | 0.003901 | Clcn6 |
| ENSMUSG00000004947 | -0.78274 | 0.00026 | 0.003907 | Dtx2 |
| ENSMUSG00000097284 | -2.1086 | 0.00026 | 0.003907 | 4930480K23Rik |
| ENSMUSG00000031851 | 0.938478 | 0.000262 | 0.003928 | Ntpcr |
| ENSMUSG00000006782 | -1.70522 | 0.000262 | 0.00393 | Cnp |
| ENSMUSG00000056899 | -1.79408 | 0.000264 | 0.003945 | Immp2l |
| ENSMUSG00000025979 | 0.811117 | 0.000264 | 0.003945 | Mob4 |
| ENSMUSG00000027187 | -0.94781 | 0.000264 | 0.003945 | Cat |
| ENSMUSG00000028221 | 0.824231 | 0.000266 | 0.00398 | Pip4p2 |
| ENSMUSG00000079487 | -0.9711 | 0.000269 | 0.004008 | Med12 |
| ENSMUSG00000032842 | -1.09345 | 0.000269 | 0.004008 | Abcc10 |
| ENSMUSG00000027217 | -1.77046 | 0.000269 | 0.004008 | Tspan18 |
| ENSMUSG00000086844 | -4.79952 | 0.000269 | 0.004008 | B230206H07Rik |
| ENSMUSG00000110697 | 2.76346 | 0.000271 | 0.004033 | Gm31718 |
| ENSMUSG00000041556 | -2.58312 | 0.000275 | 0.004092 | Fbxo2 |
| ENSMUSG00000032114 | 1.523205 | 0.000277 | 0.004114 | Slc37a4 |
| ENSMUSG00000021236 | -1.26872 | 0.000284 | 0.004211 | Entpd5 |
| ENSMUSG00000034285 | -1.45513 | 0.000284 | 0.004214 | Nipsnap1 |
| ENSMUSG00000078815 | 5.623926 | 0.000285 | 0.004229 | Cacng6 |
| ENSMUSG00000028098 | 1.045953 | 0.000286 | 0.004229 | Rnf115 |
| ENSMUSG00000051346 | 0.998679 | 0.000288 | 0.004259 | Spryd4 |
| ENSMUSG00000085438 | 0.98987 | 0.00029 | 0.004284 | 1700020I14Rik |
| ENSMUSG00000034459 | 2.832988 | 0.000292 | 0.004315 | Ifit1 |
| ENSMUSG00000032268 | -2.56123 | 0.000292 | 0.004318 | Tmprss5 |
| ENSMUSG00000055489 | 5.705143 | 0.000293 | 0.00432 | Ano5 |
| ENSMUSG00000047187 | 0.651284 | 0.000294 | 0.004331 | Rab2a |
| ENSMUSG00000073608 | -5.60604 | 0.000294 | 0.004332 | Gal3st2c |
| ENSMUSG00000028932 | 0.649919 | 0.000298 | 0.004393 | Psmc2 |
| ENSMUSG00000022684 | 0.633288 | 0.000301 | 0.00442 | Bfar |
| ENSMUSG00000026571 | 1.207576 | 0.000302 | 0.004446 | Dcaf6 |
| ENSMUSG00000030786 | 1.674969 | 0.000304 | 0.004465 | Itgam |
| ENSMUSG00000038174 | -1.08977 | 0.000304 | 0.004466 | Fam126b |
| ENSMUSG00000066842 | 1.381327 | 0.000306 | 0.004481 | Hmcn1 |
| ENSMUSG00000032010 | 1.979698 | 0.000307 | 0.004493 | Usp2 |
| ENSMUSG00000029762 | 1.218887 | 0.000307 | 0.004501 | Akr1b8 |
| ENSMUSG00000021260 | 1.339348 | 0.000308 | 0.004506 | Hhipl1 |
| ENSMUSG00000059182 | 0.726165 | 0.000309 | 0.004514 | Skap2 |
| ENSMUSG00000043557 | 1.954466 | 0.000312 | 0.004557 | Mdga1 |
| ENSMUSG00000035093 | -1.09479 | 0.000312 | 0.004557 | Secisbp2l |
| ENSMUSG00000030116 | 1.617737 | 0.000314 | 0.00458 | Mfap5 |
| ENSMUSG00000050425 | -2.08725 | 0.000315 | 0.004594 | Mrgprb2 |
| ENSMUSG00000110235 | 2.34169 | 0.000317 | 0.004614 | Gm5086 |
| ENSMUSG00000047330 | 1.596986 | 0.000318 | 0.004629 | Kcne4 |
| ENSMUSG00000035283 | -2.28977 | 0.000319 | 0.004644 | Adrb1 |
| ENSMUSG00000028434 | -1.57189 | 0.00032 | 0.004656 | Epb41l4b |
| ENSMUSG00000051355 | 0.724861 | 0.000322 | 0.004669 | Commd1 |
| ENSMUSG00000076939 | -4.67002 | 0.000324 | 0.004696 | Iglv3 |
| ENSMUSG00000020774 | -1.89219 | 0.000324 | 0.004697 | Aspa |
| ENSMUSG00000059857 | -3.3848 | 0.000327 | 0.004738 | Ntng1 |
| ENSMUSG00000086587 | -2.37046 | 0.000328 | 0.004748 | Gm11837 |
| ENSMUSG00000013584 | -1.50722 | 0.000329 | 0.004756 | Aldh1a2 |
| ENSMUSG00000025503 | -1.04987 | 0.000329 | 0.004761 | Taldo1 |
| ENSMUSG00000068747 | -1.30269 | 0.00033 | 0.00477 | Sort1 |
| ENSMUSG00000049565 | 2.077775 | 0.000331 | 0.004779 | Aknad1 |
| ENSMUSG00000115388 | -2.57517 | 0.000332 | 0.004793 | Eppk1 |
| ENSMUSG00000044122 | -1.87266 | 0.000335 | 0.004823 | Proca1 |
| ENSMUSG00000026834 | -2.49873 | 0.000336 | 0.004837 | Acvr1c |
| ENSMUSG00000010609 | -0.6889 | 0.000336 | 0.004837 | Psen2 |
| ENSMUSG00000039954 | -2.27232 | 0.000336 | 0.004837 | Stk32a |
| ENSMUSG00000026527 | -3.15672 | 0.000338 | 0.004849 | Rgs7 |
| ENSMUSG00000034634 | -3.79848 | 0.000339 | 0.004862 | Ly6d |
| ENSMUSG00000042357 | 1.560566 | 0.000342 | 0.004901 | Gjb5 |
| ENSMUSG00000039873 | 2.273214 | 0.000345 | 0.004947 | Neurl2 |
| ENSMUSG00000022635 | 0.947395 | 0.000346 | 0.004952 | Zcrb1 |
| ENSMUSG00000016477 | 1.077188 | 0.000346 | 0.004953 | E2f3 |
| ENSMUSG00000026889 | 0.749447 | 0.000348 | 0.004982 | Rbm18 |
| ENSMUSG00000032332 | 1.739423 | 0.00035 | 0.005002 | Col12a1 |
| ENSMUSG00000085022 | 3.674969 | 0.00035 | 0.005002 | Gm5860 |
| ENSMUSG00000038248 | 1.834951 | 0.000352 | 0.005023 | Sobp |
| ENSMUSG00000108801 | 1.918007 | 0.000353 | 0.00503 | Gm39090 |
| ENSMUSG00000040713 | 0.840557 | 0.000354 | 0.00504 | Creg1 |
| ENSMUSG00000028692 | 0.706897 | 0.000355 | 0.005052 | Akr1a1 |
| ENSMUSG00000035530 | 0.823242 | 0.000356 | 0.005061 | Eif1 |
| ENSMUSG00000037031 | -1.32314 | 0.000359 | 0.005103 | Tspan15 |
| ENSMUSG00000002996 | 0.682569 | 0.00036 | 0.005114 | Hbp1 |
| ENSMUSG00000068122 | 3.988495 | 0.00036 | 0.005117 | Agtr2 |
| ENSMUSG00000031144 | -1.79524 | 0.000361 | 0.005119 | Syp |
| ENSMUSG00000102037 | 2.74501 | 0.000361 | 0.00512 | Bcl2a1a |
| ENSMUSG00000035914 | 1.77097 | 0.000362 | 0.005123 | Cd276 |
| ENSMUSG00000030155 | 2.563445 | 0.000362 | 0.005123 | Clec2e |
| ENSMUSG00000045008 | 3.032252 | 0.000364 | 0.005145 | 9030612E09Rik |
| ENSMUSG00000050821 | 1.934277 | 0.000364 | 0.005146 | Fam131a |
| ENSMUSG00000026577 | 0.917545 | 0.000366 | 0.005165 | Blzf1 |
| ENSMUSG00000062380 | 1.860382 | 0.000366 | 0.005165 | Tubb3 |
| ENSMUSG00000029408 | -1.31986 | 0.000366 | 0.005165 | Abcb9 |
| ENSMUSG00000020334 | -1.39966 | 0.000367 | 0.005166 | Slc22a4 |
| ENSMUSG00000032497 | 1.061007 | 0.000367 | 0.005168 | Lrrfip2 |
| ENSMUSG00000002028 | -0.87732 | 0.000369 | 0.005191 | Kmt2a |
| ENSMUSG00000097666 | 3.451985 | 0.000369 | 0.005191 | A330094K24Rik |
| ENSMUSG00000024227 | -4.79786 | 0.000371 | 0.005214 | Pdzph1 |
| ENSMUSG00000034394 | -1.29057 | 0.000373 | 0.005243 | Lif |
| ENSMUSG00000062661 | 1.218586 | 0.000376 | 0.005272 | Ncs1 |
| ENSMUSG00000036913 | -3.76088 | 0.000377 | 0.005279 | Trim67 |
| ENSMUSG00000044037 | -1.64258 | 0.000377 | 0.005285 | Als2cl |
| ENSMUSG00000084957 | 0.786313 | 0.00038 | 0.005319 | Bbip1 |
| ENSMUSG00000029472 | 1.001012 | 0.000381 | 0.005323 | Anapc5 |
| ENSMUSG00000047976 | -3.32792 | 0.000381 | 0.005327 | Kcna1 |
| ENSMUSG00000025066 | 0.714456 | 0.000382 | 0.00533 | Sfr1 |
| ENSMUSG00000063450 | -1.15764 | 0.000382 | 0.005334 | Syne2 |
| ENSMUSG00000031444 | 2.831498 | 0.000383 | 0.005337 | F10 |
| ENSMUSG00000055413 | -1.6457 | 0.000383 | 0.005337 | H2-Q5 |
| ENSMUSG00000068617 | 3.762514 | 0.000384 | 0.005344 | Efcab1 |
| ENSMUSG00000040850 | 1.14383 | 0.000385 | 0.005361 | Psme4 |
| ENSMUSG00000031367 | 1.163347 | 0.000386 | 0.005364 | Ap1s2 |
| ENSMUSG00000021591 | 0.712096 | 0.000393 | 0.005456 | Glrx |
| ENSMUSG00000025366 | -0.82142 | 0.000393 | 0.005456 | Esyt1 |
| ENSMUSG00000040701 | -1.17541 | 0.000393 | 0.005456 | Ap1g2 |
| ENSMUSG00000064294 | -2.37697 | 0.000396 | 0.005489 | Aox3 |
| ENSMUSG00000016262 | 1.381356 | 0.000398 | 0.005517 | Sertad4 |
| ENSMUSG00000098188 | -1.29385 | 0.000399 | 0.005519 | Sowahc |
| ENSMUSG00000004609 | 0.90493 | 0.000399 | 0.005519 | Cd33 |
| ENSMUSG00000049130 | 1.104549 | 0.0004 | 0.005533 | C5ar1 |
| ENSMUSG00000032816 | 1.387585 | 0.000404 | 0.005585 | Igdcc4 |
| ENSMUSG00000002266 | 2.407687 | 0.000405 | 0.005598 | Zim1 |
| ENSMUSG00000063524 | -1.20379 | 0.000407 | 0.005622 | Eno1 |
| ENSMUSG00000109293 | -2.20534 | 0.000412 | 0.005682 | Dcst2 |
| ENSMUSG00000097462 | -1.67882 | 0.000412 | 0.005682 | 9530026P05Rik |
| ENSMUSG00000068391 | 0.697525 | 0.000413 | 0.005682 | Chrac1 |
| ENSMUSG00000089781 | 5.694435 | 0.000417 | 0.005732 | Gm15756 |
| ENSMUSG00000056174 | 2.096475 | 0.000419 | 0.005761 | Col8a2 |
| ENSMUSG00000027861 | 2.23014 | 0.000419 | 0.005761 | Casq2 |
| ENSMUSG00000034295 | 2.140563 | 0.000421 | 0.005776 | Fhod3 |
| ENSMUSG00000031134 | -0.84094 | 0.000423 | 0.005804 | Rbmx |
| ENSMUSG00000111818 | 4.049903 | 0.000423 | 0.005804 | Gm17749 |
| ENSMUSG00000032401 | -3.02653 | 0.000426 | 0.005836 | Lctl |
| ENSMUSG00000022803 | 1.830486 | 0.000428 | 0.005865 | Popdc2 |
| ENSMUSG00000002565 | -1.44412 | 0.000429 | 0.005869 | Scin |
| ENSMUSG00000021917 | 0.696842 | 0.000431 | 0.005889 | Spcs1 |
| ENSMUSG00000000730 | 1.883778 | 0.000438 | 0.00598 | Dnmt3l |
| ENSMUSG00000048355 | -2.72514 | 0.000442 | 0.006027 | Arxes1 |
| ENSMUSG00000042700 | -1.22983 | 0.000444 | 0.006055 | Sipa1l1 |
| ENSMUSG00000025938 | 2.912746 | 0.000444 | 0.006055 | Slco5a1 |
| ENSMUSG00000033762 | -2.06611 | 0.000448 | 0.006101 | Recql4 |
| ENSMUSG00000025576 | -2.63973 | 0.000448 | 0.006101 | Rbfox3 |
| ENSMUSG00000052688 | 1.190377 | 0.000451 | 0.006129 | Rab7b |
| ENSMUSG00000110018 | 5.256309 | 0.000451 | 0.006129 | 5430437J10Rik |
| ENSMUSG00000056427 | -1.66478 | 0.000452 | 0.006143 | Slit3 |
| ENSMUSG00000022562 | -0.99624 | 0.000453 | 0.006153 | Oplah |
| ENSMUSG00000063193 | 2.20098 | 0.000456 | 0.006181 | Cd300lb |
| ENSMUSG00000036943 | 0.751476 | 0.000457 | 0.006197 | Rab8b |
| ENSMUSG00000029304 | 4.42933 | 0.000458 | 0.006198 | Spp1 |
| ENSMUSG00000011752 | -0.71881 | 0.00046 | 0.00622 | Pgam1 |
| ENSMUSG00000024411 | 5.09804 | 0.000461 | 0.006238 | Aqp4 |
| ENSMUSG00000029641 | -2.10371 | 0.000462 | 0.006244 | Rasl11a |
| ENSMUSG00000034881 | -1.38146 | 0.000464 | 0.006263 | Tbxa2r |
| ENSMUSG00000043673 | -1.78063 | 0.000468 | 0.006316 | Kcns3 |
| ENSMUSG00000027784 | 1.824332 | 0.000469 | 0.006316 | Ppm1l |
| ENSMUSG00000026638 | -1.9343 | 0.000469 | 0.006316 | Irf6 |
| ENSMUSG00000091264 | 1.052107 | 0.000472 | 0.006353 | Smim13 |
| ENSMUSG00000014164 | -1.44615 | 0.000474 | 0.006373 | Klhl3 |
| ENSMUSG00000091694 | -4.55485 | 0.000474 | 0.006377 | Apol11b |
| ENSMUSG00000050377 | -2.9642 | 0.000477 | 0.00641 | Il31ra |
| ENSMUSG00000035296 | 3.151315 | 0.000481 | 0.006455 | Sgcg |
| ENSMUSG00000104350 | -4.37386 | 0.000483 | 0.006478 | Gm38244 |
| ENSMUSG00000042961 | 1.741646 | 0.000483 | 0.006484 | Egflam |
| ENSMUSG00000107244 | 5.618088 | 0.000484 | 0.00649 | Gm43699 |
| ENSMUSG00000024048 | 0.767046 | 0.000486 | 0.006504 | Myl12a |
| ENSMUSG00000042507 | -0.74615 | 0.000488 | 0.006527 | Elmsan1 |
| ENSMUSG00000030275 | -0.751 | 0.000488 | 0.006532 | Etnk1 |
| ENSMUSG00000020949 | 1.149889 | 0.00049 | 0.006542 | Fkbp3 |
| ENSMUSG00000039686 | -0.69837 | 0.00049 | 0.006545 | Zer1 |
| ENSMUSG00000021712 | 0.812025 | 0.000491 | 0.006557 | Trim23 |
| ENSMUSG00000047945 | 0.975513 | 0.000492 | 0.006561 | Marcksl1 |
| ENSMUSG00000022905 | 1.351252 | 0.000492 | 0.006561 | Kpna1 |
| ENSMUSG00000074109 | -2.48581 | 0.000494 | 0.006576 | Mrgprx2 |
| ENSMUSG00000024914 | 1.104867 | 0.000497 | 0.006608 | Drap1 |
| ENSMUSG00000074206 | -2.16539 | 0.000497 | 0.006608 | Adh6b |
| ENSMUSG00000085558 | -1.36615 | 0.000498 | 0.006608 | 4930412C18Rik |
| ENSMUSG00000078122 | 1.187428 | 0.000498 | 0.006608 | F630028O10Rik |
| ENSMUSG00000019982 | -3.53541 | 0.000498 | 0.006608 | Myb |
| ENSMUSG00000025347 | -3.70861 | 0.000499 | 0.006615 | Mettl7b |
| ENSMUSG00000041650 | -1.38358 | 0.000502 | 0.006652 | Pcca |
| ENSMUSG00000059213 | -4.62082 | 0.000502 | 0.006652 | Ddn |
| ENSMUSG00000028911 | -0.77806 | 0.000505 | 0.006683 | Srsf4 |
| ENSMUSG00000031740 | 1.656893 | 0.000509 | 0.006735 | Mmp2 |
| ENSMUSG00000032338 | -2.78032 | 0.000512 | 0.006766 | Hcn4 |
| ENSMUSG00000109773 | -4.44965 | 0.000514 | 0.00679 | Gm34474 |
| ENSMUSG00000024163 | -0.82843 | 0.000521 | 0.006882 | Mapk8ip3 |
| ENSMUSG00000028953 | 0.873121 | 0.000529 | 0.006971 | Abcf2 |
| ENSMUSG00000028420 | 1.096308 | 0.000536 | 0.007063 | Tmem38b |
| ENSMUSG00000001333 | 2.163101 | 0.000537 | 0.007067 | Sync |
| ENSMUSG00000032500 | -2.37127 | 0.000537 | 0.007067 | Dclk3 |
| ENSMUSG00000020794 | 1.054963 | 0.000541 | 0.007122 | Ube2g1 |
| ENSMUSG00000056602 | -1.08458 | 0.000546 | 0.007178 | Fry |
| ENSMUSG00000032777 | -0.70546 | 0.000547 | 0.00719 | Gtf3c1 |
| ENSMUSG00000043795 | 1.538623 | 0.000552 | 0.007249 | Prr33 |
| ENSMUSG00000027068 | 1.29893 | 0.000558 | 0.007326 | Dhrs9 |
| ENSMUSG00000031007 | 0.694329 | 0.000559 | 0.007326 | Atp6ap2 |
| ENSMUSG00000057286 | -1.42376 | 0.000559 | 0.007326 | St6galnac2 |
| ENSMUSG00000057672 | -0.8122 | 0.00056 | 0.007326 | Pkn1 |
| ENSMUSG00000043873 | -2.84786 | 0.000561 | 0.007342 | Chil5 |
| ENSMUSG00000028251 | 1.17535 | 0.000563 | 0.007356 | Tstd3 |
| ENSMUSG00000028088 | -1.13513 | 0.000564 | 0.007366 | Fmo5 |
| ENSMUSG00000044162 | 2.140812 | 0.000564 | 0.007368 | Tnip3 |
| ENSMUSG00000040740 | -2.14579 | 0.000565 | 0.007373 | Slc25a34 |
| ENSMUSG00000024664 | -1.19146 | 0.000566 | 0.007378 | Fads3 |
| ENSMUSG00000028238 | 2.867852 | 0.000567 | 0.007384 | Atp6v0d2 |
| ENSMUSG00000062270 | 0.664166 | 0.000569 | 0.007406 | Morf4l1 |
| ENSMUSG00000009214 | 4.699077 | 0.000571 | 0.00743 | Mymk |
| ENSMUSG00000036093 | 0.718678 | 0.000578 | 0.007509 | Arl5a |
| ENSMUSG00000030329 | 2.190245 | 0.000578 | 0.007509 | Pianp |
| ENSMUSG00000051029 | 3.514036 | 0.000578 | 0.007509 | Serpinb1b |
| ENSMUSG00000045318 | -3.54483 | 0.000579 | 0.007515 | Adra2c |
| ENSMUSG00000018287 | 0.788218 | 0.00058 | 0.007516 | Spag7 |
| ENSMUSG00000027452 | -1.07205 | 0.00058 | 0.00752 | Acss1 |
| ENSMUSG00000037922 | -2.64721 | 0.000581 | 0.007529 | Bank1 |
| ENSMUSG00000034164 | 1.472456 | 0.000586 | 0.007585 | Emid1 |
| ENSMUSG00000095335 | -7.34394 | 0.000587 | 0.007586 | Igkv3-5 |
| ENSMUSG00000037904 | 1.975256 | 0.000587 | 0.007588 | Ankrd9 |
| ENSMUSG00000019795 | 0.891705 | 0.000592 | 0.007647 | Pcmt1 |
| ENSMUSG00000026150 | 0.70442 | 0.000595 | 0.00767 | Mff |
| ENSMUSG00000018362 | 1.052973 | 0.000595 | 0.00767 | Kpna2 |
| ENSMUSG00000030470 | 6.447417 | 0.000595 | 0.007672 | Csrp3 |
| ENSMUSG00000006998 | 0.694907 | 0.000596 | 0.007683 | Psmd2 |
| ENSMUSG00000057469 | 1.323068 | 0.000599 | 0.007704 | E2f6 |
| ENSMUSG00000024621 | 0.69188 | 0.0006 | 0.007718 | Csf1r |
| ENSMUSG00000026177 | 1.192982 | 0.000601 | 0.007726 | Slc11a1 |
| ENSMUSG00000042770 | -1.07108 | 0.000602 | 0.007726 | Hebp1 |
| ENSMUSG00000023959 | 1.772092 | 0.000602 | 0.007726 | Clic5 |
| ENSMUSG00000087090 | 5.527683 | 0.000603 | 0.00773 | Nctc1 |
| ENSMUSG00000003477 | -2.07826 | 0.000606 | 0.007772 | Inmt |
| ENSMUSG00000018599 | 1.22542 | 0.00061 | 0.007813 | Mief2 |
| ENSMUSG00000070469 | -1.96566 | 0.000611 | 0.007813 | Adamtsl3 |
| ENSMUSG00000019762 | -5.34133 | 0.000611 | 0.007813 | Iyd |
| ENSMUSG00000021559 | -1.33465 | 0.000614 | 0.007846 | Dapk1 |
| ENSMUSG00000020328 | 0.72418 | 0.000615 | 0.007855 | Nudcd2 |
| ENSMUSG00000068749 | 0.715009 | 0.000617 | 0.007878 | Psma5 |
| ENSMUSG00000038145 | -0.85335 | 0.000618 | 0.00788 | Snrk |
| ENSMUSG00000024130 | -0.9467 | 0.000627 | 0.007992 | Abca3 |
| ENSMUSG00000042129 | 0.908367 | 0.000631 | 0.008036 | Rassf4 |
| ENSMUSG00000037138 | -1.13619 | 0.000631 | 0.008036 | Aff3 |
| ENSMUSG00000047363 | 2.472893 | 0.000638 | 0.008117 | Cstad |
| ENSMUSG00000032528 | -2.81529 | 0.000639 | 0.008124 | Vipr1 |
| ENSMUSG00000051985 | 6.519209 | 0.000642 | 0.008164 | Igfn1 |
| ENSMUSG00000056698 | -0.84008 | 0.000644 | 0.008173 | Elmod3 |
| ENSMUSG00000001376 | 0.724385 | 0.000645 | 0.008184 | Vps50 |
| ENSMUSG00000017897 | -1.53183 | 0.000646 | 0.008193 | Eya2 |
| ENSMUSG00000062609 | -1.26386 | 0.000646 | 0.008193 | Kcnj15 |
| ENSMUSG00000021774 | 0.753724 | 0.000647 | 0.008197 | Ube2e1 |
| ENSMUSG00000047992 | -3.04544 | 0.000648 | 0.008208 | Fam69c |
| ENSMUSG00000021646 | -1.15624 | 0.000655 | 0.008281 | Mccc2 |
| ENSMUSG00000064280 | 3.481979 | 0.000658 | 0.008317 | Ccdc146 |
| ENSMUSG00000030688 | 0.96519 | 0.000661 | 0.008347 | Stard10 |
| ENSMUSG00000111535 | 2.632412 | 0.000661 | 0.008348 | Gm35154 |
| ENSMUSG00000020173 | 2.192375 | 0.000665 | 0.008386 | Cobl |
| ENSMUSG00000051255 | 0.727517 | 0.000666 | 0.008401 | Gm6563 |
| ENSMUSG00000019054 | 0.664323 | 0.000669 | 0.008426 | Fis1 |
| ENSMUSG00000044083 | -3.66135 | 0.000672 | 0.008464 | Efcab8 |
| ENSMUSG00000040350 | 1.998638 | 0.000675 | 0.00849 | Trim7 |
| ENSMUSG00000034205 | 1.534729 | 0.000677 | 0.008511 | Loxl2 |
| ENSMUSG00000028964 | 1.088148 | 0.000681 | 0.008552 | Park7 |
| ENSMUSG00000023968 | 2.669853 | 0.000682 | 0.008564 | Crip3 |
| ENSMUSG00000037725 | 2.038579 | 0.000685 | 0.008602 | Ckap2 |
| ENSMUSG00000047517 | -6.88376 | 0.000689 | 0.008645 | Dmbt1 |
| ENSMUSG00000029176 | 0.607447 | 0.000693 | 0.008692 | Anapc4 |
| ENSMUSG00000021238 | -1.4862 | 0.000696 | 0.008723 | Aldh6a1 |
| ENSMUSG00000087380 | 1.257711 | 0.000704 | 0.008818 | 2210408F21Rik |
| ENSMUSG00000040133 | 2.494178 | 0.000705 | 0.008818 | Gpr176 |
| ENSMUSG00000052776 | 1.160501 | 0.000711 | 0.008886 | Oas1a |
| ENSMUSG00000030223 | 0.999018 | 0.000711 | 0.008886 | Ptpro |
| ENSMUSG00000054874 | -0.77081 | 0.000716 | 0.008943 | Pcnx3 |
| ENSMUSG00000056666 | -1.06937 | 0.000717 | 0.008949 | Retsat |
| ENSMUSG00000029036 | -0.72342 | 0.000718 | 0.008952 | Atad3a |
| ENSMUSG00000117104 | 3.695534 | 0.00072 | 0.008976 | AC166164.1 |
| ENSMUSG00000018428 | -1.24241 | 0.000721 | 0.008981 | Akap1 |
| ENSMUSG00000042249 | -1.102 | 0.000723 | 0.008994 | Grk3 |
| ENSMUSG00000039629 | -1.16785 | 0.000728 | 0.009055 | Strip2 |
| ENSMUSG00000071424 | -3.33953 | 0.000729 | 0.009055 | Grid2 |
| ENSMUSG00000026253 | 5.831964 | 0.00073 | 0.009064 | Chrng |
| ENSMUSG00000023571 | -0.95212 | 0.000732 | 0.009085 | C1qtnf12 |
| ENSMUSG00000110206 | -1.11184 | 0.000733 | 0.009091 | Flt3l |
| ENSMUSG00000006221 | 2.362076 | 0.000742 | 0.009194 | Hspb7 |
| ENSMUSG00000087611 | -2.25328 | 0.000744 | 0.009215 | 4930458D05Rik |
| ENSMUSG00000028289 | -1.95605 | 0.000746 | 0.009231 | Epha7 |
| ENSMUSG00000035279 | 1.416771 | 0.000746 | 0.009233 | Ssc5d |
| ENSMUSG00000030117 | 2.552012 | 0.000751 | 0.009289 | Gdf3 |
| ENSMUSG00000041329 | 2.169342 | 0.000757 | 0.009353 | Atp1b2 |
| ENSMUSG00000044026 | -1.23248 | 0.00076 | 0.009379 | Slc35g1 |
| ENSMUSG00000063296 | 2.562656 | 0.000763 | 0.009411 | Tmem117 |
| ENSMUSG00000065952 | -6.04533 | 0.000765 | 0.00944 | C330021F23Rik |
| ENSMUSG00000035578 | 1.931763 | 0.000766 | 0.009446 | Iqcg |
| ENSMUSG00000026678 | 1.433663 | 0.000769 | 0.009469 | Rgs5 |
| ENSMUSG00000022407 | 1.099651 | 0.000771 | 0.009487 | Adsl |
| ENSMUSG00000051111 | -2.8699 | 0.000775 | 0.00953 | Sv2c |
| ENSMUSG00000081684 | 0.828005 | 0.000782 | 0.009619 | Rps2-ps13 |
| ENSMUSG00000022340 | -1.66565 | 0.000783 | 0.00962 | Sybu |
| ENSMUSG00000018559 | 0.953623 | 0.000787 | 0.009654 | Ctdnep1 |
| ENSMUSG00000097339 | -4.47894 | 0.000787 | 0.009654 | Gm26671 |
| ENSMUSG00000033253 | -0.84658 | 0.000792 | 0.009713 | Szt2 |
| ENSMUSG00000040746 | -1.1556 | 0.000796 | 0.009753 | Rnf167 |
| ENSMUSG00000052825 | 0.784524 | 0.000796 | 0.009753 | Gm9892 |
| ENSMUSG00000018387 | -2.17217 | 0.000799 | 0.00978 | Shroom1 |
| ENSMUSG00000028121 | -0.94858 | 0.000802 | 0.009805 | Bcar3 |
| ENSMUSG00000055128 | 0.750956 | 0.000806 | 0.009851 | Cgrrf1 |
| ENSMUSG00000014606 | 0.960747 | 0.000806 | 0.009851 | Slc25a11 |
| ENSMUSG00000100807 | 4.356204 | 0.000808 | 0.009867 | Gm29521 |
| ENSMUSG00000028975 | -0.96172 | 0.000811 | 0.009893 | Pex14 |
| ENSMUSG00000034171 | -2.83295 | 0.000812 | 0.009901 | Faah |
| ENSMUSG00000036086 | 1.340746 | 0.000816 | 0.00994 | Zranb3 |
| ENSMUSG00000031980 | -2.11431 | 0.000817 | 0.009943 | Agt |
| ENSMUSG00000021765 | -1.09326 | 0.000817 | 0.009944 | Fst |
| ENSMUSG00000052629 | -2.72898 | 0.000818 | 0.009944 | Gm9885 |
| ENSMUSG00000031295 | -0.95477 | 0.000819 | 0.009958 | Phka2 |
| ENSMUSG00000044433 | -1.63584 | 0.000821 | 0.009968 | Camsap3 |
| ENSMUSG00000030143 | -9.05352 | 0.000831 | 0.010088 | Gm8882 |
| ENSMUSG00000095351 | -5.20617 | 0.000832 | 0.010088 | Igkv3-2 |
| ENSMUSG00000013523 | -2.74608 | 0.000832 | 0.010088 | Bcas1 |
| ENSMUSG00000019832 | 0.803653 | 0.000835 | 0.01011 | Rab32 |
| ENSMUSG00000091345 | 1.803109 | 0.000838 | 0.010144 | Col6a5 |
| ENSMUSG00000018774 | 1.130752 | 0.000839 | 0.010152 | Cd68 |
| ENSMUSG00000091509 | 1.126636 | 0.00084 | 0.010152 | Gm17066 |
| ENSMUSG00000034165 | -0.62277 | 0.000842 | 0.010172 | Ccnd3 |
| ENSMUSG00000057329 | -1.10984 | 0.000843 | 0.010176 | Bcl2 |
| ENSMUSG00000072980 | 1.868146 | 0.000843 | 0.010179 | Oip5 |
| ENSMUSG00000106353 | 6.243231 | 0.000845 | 0.010191 | Gm43726 |
| ENSMUSG00000026251 | 5.014006 | 0.000846 | 0.010193 | Chrnd |
| ENSMUSG00000031467 | 0.87407 | 0.000848 | 0.010215 | Agpat5 |
| ENSMUSG00000027401 | -2.7825 | 0.00085 | 0.010236 | Tgm3 |
| ENSMUSG00000039114 | -2.1845 | 0.000853 | 0.010257 | Nrn1 |
| ENSMUSG00000032349 | -0.83144 | 0.000853 | 0.010257 | Elovl5 |
| ENSMUSG00000029245 | -3.00836 | 0.000856 | 0.010265 | Epha5 |
| ENSMUSG00000005803 | -0.79321 | 0.000856 | 0.010265 | Sqor |
| ENSMUSG00000023232 | 3.062211 | 0.000856 | 0.010265 | Serinc2 |
| ENSMUSG00000060288 | 2.068023 | 0.000856 | 0.010265 | Ppih |
| ENSMUSG00000042763 | -2.81257 | 0.000856 | 0.010265 | Maneal |
| ENSMUSG00000007613 | 0.805264 | 0.000862 | 0.010324 | Tgfbr1 |
| ENSMUSG00000021943 | -1.84619 | 0.000863 | 0.010326 | Gdf10 |
| ENSMUSG00000020741 | -1.19976 | 0.000865 | 0.010351 | Cluh |
| ENSMUSG00000019920 | 0.729688 | 0.000866 | 0.010352 | Lims1 |
| ENSMUSG00000040522 | 1.107334 | 0.000868 | 0.010371 | Tlr8 |
| ENSMUSG00000071708 | 1.874637 | 0.00087 | 0.010387 | Sms |
| ENSMUSG00000101941 | 5.758846 | 0.000872 | 0.010411 | Gm28979 |
| ENSMUSG00000031266 | 1.345942 | 0.000875 | 0.01043 | Gla |
| ENSMUSG00000003352 | 1.388249 | 0.000878 | 0.010462 | Cacnb3 |
| ENSMUSG00000074604 | -2.40946 | 0.00088 | 0.010481 | Mgst2 |
| ENSMUSG00000039057 | 3.825992 | 0.000885 | 0.010536 | Myo16 |
| ENSMUSG00000027210 | -0.84405 | 0.000891 | 0.010595 | Meis2 |
| ENSMUSG00000021319 | 2.245746 | 0.000896 | 0.010642 | Sfrp4 |
| ENSMUSG00000035041 | 2.372276 | 0.000896 | 0.010642 | Creb3l3 |
| ENSMUSG00000039166 | -0.87048 | 0.000896 | 0.010642 | Akap7 |
| ENSMUSG00000041688 | 1.281394 | 0.000899 | 0.010659 | Amot |
| ENSMUSG00000030515 | 1.504057 | 0.000899 | 0.010659 | Tarsl2 |
| ENSMUSG00000028980 | -0.7378 | 0.000903 | 0.010707 | H6pd |
| ENSMUSG00000055320 | 1.095976 | 0.000908 | 0.010757 | Tead1 |
| ENSMUSG00000089703 | 5.810858 | 0.000912 | 0.010799 | Gm15833 |
| ENSMUSG00000001020 | 2.089751 | 0.000914 | 0.010816 | S100a4 |
| ENSMUSG00000073700 | 1.248063 | 0.000918 | 0.010848 | Klhl21 |
| ENSMUSG00000027580 | -1.08658 | 0.00092 | 0.010866 | Helz2 |
| ENSMUSG00000025092 | -1.25878 | 0.000923 | 0.010894 | Hspa12a |
| ENSMUSG00000062515 | -1.94657 | 0.000927 | 0.010935 | Fabp4 |
| ENSMUSG00000026494 | 1.670397 | 0.000929 | 0.010962 | Kif26b |
| ENSMUSG00000029335 | -1.57743 | 0.000931 | 0.010973 | Bmp3 |
| ENSMUSG00000020440 | 1.01351 | 0.000934 | 0.011001 | Arf5 |
| ENSMUSG00000044103 | 3.414496 | 0.000935 | 0.011011 | Il1f9 |
| ENSMUSG00000070577 | 3.294079 | 0.000936 | 0.011018 | Gm572 |
| ENSMUSG00000045569 | -2.55282 | 0.000939 | 0.011035 | Mc2r |
| ENSMUSG00000000732 | -1.15058 | 0.00094 | 0.011035 | Icosl |
| ENSMUSG00000042616 | -0.95632 | 0.00094 | 0.011035 | Oscp1 |
| ENSMUSG00000025612 | 0.615958 | 0.00094 | 0.011035 | Bach1 |
| ENSMUSG00000025332 | -0.66896 | 0.000941 | 0.011039 | Kdm5c |
| ENSMUSG00000021662 | -1.31872 | 0.000942 | 0.011039 | Arhgef28 |
| ENSMUSG00000109598 | -6.25697 | 0.000943 | 0.011039 | Gm36356 |
| ENSMUSG00000020682 | -2.027 | 0.000943 | 0.011039 | Mmp28 |
| ENSMUSG00000005951 | -1.64781 | 0.000943 | 0.011039 | Shpk |
| ENSMUSG00000036766 | -3.21977 | 0.000948 | 0.01108 | Dner |
| ENSMUSG00000022996 | -1.44957 | 0.000949 | 0.011089 | Wnt10b |
| ENSMUSG00000098882 | -6.04027 | 0.000951 | 0.011102 | Mir6392 |
| ENSMUSG00000039660 | -0.76062 | 0.000951 | 0.011102 | Spout1 |
| ENSMUSG00000026459 | 5.245186 | 0.000959 | 0.011185 | Myog |
| ENSMUSG00000059895 | 1.47825 | 0.000963 | 0.01123 | Ptp4a3 |
| ENSMUSG00000025020 | 2.485314 | 0.000969 | 0.011284 | Slit1 |
| ENSMUSG00000022759 | 2.55717 | 0.000969 | 0.011284 | Lrrc74b |
| ENSMUSG00000055421 | -3.32453 | 0.000971 | 0.011297 | Pcdh9 |
| ENSMUSG00000097391 | 1.908147 | 0.000972 | 0.011306 | Mirg |
| ENSMUSG00000102715 | 5.478825 | 0.000986 | 0.011454 | Gm6209 |
| ENSMUSG00000070407 | -2.38542 | 0.000986 | 0.011454 | Hs3st3b1 |
| ENSMUSG00000038644 | -1.03636 | 0.000988 | 0.011473 | Pold1 |
| ENSMUSG00000018417 | 0.78109 | 0.000991 | 0.011501 | Myo1b |
| ENSMUSG00000044694 | -1.94918 | 0.000998 | 0.011566 | 2010007H06Rik |
| ENSMUSG00000081769 | -1.01837 | 0.000999 | 0.01157 | Gm12216 |
| ENSMUSG00000028937 | 0.943297 | 0.001005 | 0.011633 | Acot7 |
| ENSMUSG00000022677 | 0.811208 | 0.001007 | 0.011648 | Fopnl |
| ENSMUSG00000116879 | 2.366012 | 0.00101 | 0.011683 | AC125141.3 |
| ENSMUSG00000071647 | -0.64778 | 0.001014 | 0.01172 | Eml3 |
| ENSMUSG00000032387 | -1.63355 | 0.001014 | 0.01172 | Rbpms2 |
| ENSMUSG00000037370 | 1.195533 | 0.001019 | 0.011769 | Enpp1 |
| ENSMUSG00000028766 | -1.80453 | 0.00102 | 0.011772 | Alpl |
| ENSMUSG00000030417 | 0.772759 | 0.001021 | 0.011773 | Pdcd5 |
| ENSMUSG00000049999 | -1.56891 | 0.001025 | 0.011807 | Ppp1r3d |
| ENSMUSG00000068874 | -0.97486 | 0.001025 | 0.011807 | Selenbp1 |
| ENSMUSG00000028430 | -0.74084 | 0.00103 | 0.011857 | Nol6 |
| ENSMUSG00000022667 | 1.625031 | 0.001034 | 0.011897 | Cd200r1 |
| ENSMUSG00000101585 | 1.868832 | 0.001038 | 0.011926 | 1600010M07Rik |
| ENSMUSG00000027777 | 2.115601 | 0.001038 | 0.011926 | Schip1 |
| ENSMUSG00000037541 | -3.01347 | 0.001044 | 0.011984 | Shank2 |
| ENSMUSG00000025369 | -0.6625 | 0.001046 | 0.011998 | Smarcc2 |
| ENSMUSG00000046159 | -1.63783 | 0.001049 | 0.012029 | Chrm3 |
| ENSMUSG00000030956 | -0.68762 | 0.001053 | 0.012064 | Fam53b |
| ENSMUSG00000045802 | -2.92599 | 0.001056 | 0.012097 | Hsf3 |
| ENSMUSG00000019312 | -2.34187 | 0.001059 | 0.012127 | Grb7 |
| ENSMUSG00000086604 | 2.074808 | 0.00106 | 0.012132 | Gm15510 |
| ENSMUSG00000025035 | 0.763409 | 0.001064 | 0.012162 | Arl3 |
| ENSMUSG00000021196 | -0.64331 | 0.001066 | 0.012188 | Pfkp |
| ENSMUSG00000061535 | 1.540049 | 0.001068 | 0.012201 | C1qtnf7 |
| ENSMUSG00000069792 | 1.412408 | 0.001069 | 0.012207 | Wfdc17 |
| ENSMUSG00000028643 | 0.857612 | 0.001071 | 0.012215 | Svbp |
| ENSMUSG00000091512 | 0.631588 | 0.001077 | 0.012283 | Lamtor3 |
| ENSMUSG00000031278 | 0.8242 | 0.001079 | 0.012293 | Acsl4 |
| ENSMUSG00000026691 | -3.0895 | 0.00108 | 0.012293 | Fmo3 |
| ENSMUSG00000020431 | -2.31371 | 0.001083 | 0.012326 | Adcy1 |
| ENSMUSG00000028086 | 0.874575 | 0.001085 | 0.012337 | Fbxw7 |
| ENSMUSG00000099137 | -2.62918 | 0.001086 | 0.012347 | Gm10603 |
| ENSMUSG00000063894 | -0.64745 | 0.001087 | 0.012347 | Zkscan8 |
| ENSMUSG00000000915 | -1.42897 | 0.00109 | 0.012377 | Hip1r |
| ENSMUSG00000038059 | 0.871181 | 0.001092 | 0.012391 | Smim3 |
| ENSMUSG00000028884 | 1.027027 | 0.001093 | 0.012399 | Rpa2 |
| ENSMUSG00000097378 | -2.80518 | 0.001096 | 0.012417 | B230208H11Rik |
| ENSMUSG00000031016 | 1.311135 | 0.001098 | 0.012435 | Wee1 |
| ENSMUSG00000054752 | 1.518931 | 0.001104 | 0.012491 | Fsd1l |
| ENSMUSG00000020961 | 0.812421 | 0.001104 | 0.012491 | Ston2 |
| ENSMUSG00000028576 | 0.929736 | 0.001108 | 0.012528 | Ift74 |
| ENSMUSG00000004568 | -1.23539 | 0.00111 | 0.012549 | Arhgef18 |
| ENSMUSG00000064193 | -1.2753 | 0.001113 | 0.012565 | Gm4735 |
| ENSMUSG00000044751 | 1.25811 | 0.001114 | 0.012565 | Gm12231 |
| ENSMUSG00000063531 | -1.34452 | 0.001114 | 0.012565 | Sema3e |
| ENSMUSG00000061119 | 1.120755 | 0.00112 | 0.012629 | Prcp |
| ENSMUSG00000001211 | -1.04532 | 0.001122 | 0.012638 | Agpat3 |
| ENSMUSG00000100658 | -1.93351 | 0.001123 | 0.01264 | F730311O21Rik |
| ENSMUSG00000002475 | -1.20997 | 0.001133 | 0.012753 | Abhd3 |
| ENSMUSG00000024640 | -0.75985 | 0.001134 | 0.012754 | Psat1 |
| ENSMUSG00000024210 | 1.670905 | 0.001138 | 0.012795 | Ip6k3 |
| ENSMUSG00000027582 | -0.74101 | 0.001144 | 0.012847 | Zgpat |
| ENSMUSG00000038193 | -4.35178 | 0.001145 | 0.012853 | Hand2 |
| ENSMUSG00000026679 | 1.399517 | 0.001148 | 0.012884 | Enkur |
| ENSMUSG00000018821 | 0.839456 | 0.00115 | 0.012886 | Avpi1 |
| ENSMUSG00000066632 | 1.868824 | 0.00115 | 0.012886 | Pgk1-rs7 |
| ENSMUSG00000044303 | 2.605138 | 0.001152 | 0.012902 | Cdkn2a |
| ENSMUSG00000000811 | -0.87099 | 0.001153 | 0.012902 | Txnrd3 |
| ENSMUSG00000050052 | -2.12827 | 0.001154 | 0.012913 | Tdrp |
| ENSMUSG00000011382 | -1.30413 | 0.001158 | 0.012946 | Dhdh |
| ENSMUSG00000049577 | -1.20666 | 0.001159 | 0.012946 | Zfpm1 |
| ENSMUSG00000042265 | 4.22559 | 0.001161 | 0.012957 | Trem1 |
| ENSMUSG00000074896 | 2.070464 | 0.001161 | 0.012957 | Ifit3 |
| ENSMUSG00000035944 | -0.86804 | 0.001161 | 0.012957 | Ttc38 |
| ENSMUSG00000027961 | 1.696783 | 0.001163 | 0.012966 | Lrrc39 |
| ENSMUSG00000087064 | -3.87038 | 0.001168 | 0.013016 | Sap30bpos |
| ENSMUSG00000027810 | 0.580716 | 0.001172 | 0.013055 | Eif2a |
| ENSMUSG00000090667 | 2.623466 | 0.001174 | 0.013061 | Gm765 |
| ENSMUSG00000021234 | -1.64948 | 0.001178 | 0.013107 | Fam161b |
| ENSMUSG00000026924 | -0.60597 | 0.001185 | 0.013173 | Sec16a |
| ENSMUSG00000024781 | 1.155019 | 0.00119 | 0.013217 | Lipa |
| ENSMUSG00000041084 | 0.811814 | 0.00119 | 0.013217 | Ostc |
| ENSMUSG00000078862 | 0.854177 | 0.001193 | 0.013223 | Gm14326 |
| ENSMUSG00000024948 | -1.42962 | 0.001193 | 0.013223 | Map4k2 |
| ENSMUSG00000021816 | 1.06919 | 0.001193 | 0.013223 | Ppp3cb |
| ENSMUSG00000030301 | 1.006362 | 0.001199 | 0.013285 | Ccdc91 |
| ENSMUSG00000053453 | 0.819381 | 0.001202 | 0.013309 | Thoc7 |
| ENSMUSG00000038000 | -0.7213 | 0.001203 | 0.013316 | Acd |
| ENSMUSG00000033192 | 0.903593 | 0.001215 | 0.013438 | Lpcat2 |
| ENSMUSG00000072915 | -1.07388 | 0.001222 | 0.013503 | Gm12258 |
| ENSMUSG00000031838 | 1.324296 | 0.001239 | 0.013689 | Ifi30 |
| ENSMUSG00000020598 | -2.00525 | 0.001242 | 0.013713 | Nrcam |
| ENSMUSG00000032554 | -0.72261 | 0.001246 | 0.013752 | Trf |
| ENSMUSG00000026473 | -0.86409 | 0.001248 | 0.013753 | Glul |
| ENSMUSG00000017756 | -1.27212 | 0.001248 | 0.013753 | Slc12a7 |
| ENSMUSG00000084960 | -3.02337 | 0.001254 | 0.013807 | B430010I23Rik |
| ENSMUSG00000048915 | -1.95086 | 0.001256 | 0.013824 | Efna5 |
| ENSMUSG00000097174 | -1.69028 | 0.001257 | 0.013828 | Gm4890 |
| ENSMUSG00000045620 | -2.21857 | 0.001259 | 0.013841 | Odf3l1 |
| ENSMUSG00000031609 | 1.313178 | 0.001265 | 0.013906 | Sap30 |
| ENSMUSG00000074483 | -5.80075 | 0.00127 | 0.013949 | Bglap |
| ENSMUSG00000018661 | -0.66885 | 0.001271 | 0.013949 | Cog1 |
| ENSMUSG00000044703 | 1.804203 | 0.001273 | 0.01396 | Phf11a |
| ENSMUSG00000109643 | 5.724277 | 0.001288 | 0.014122 | Gm31545 |
| ENSMUSG00000021573 | -0.8767 | 0.001289 | 0.014124 | Tppp |
| ENSMUSG00000027513 | -2.45066 | 0.00129 | 0.014126 | Pck1 |
| ENSMUSG00000015947 | 1.544509 | 0.001291 | 0.014126 | Fcgr1 |
| ENSMUSG00000106871 | -3.64729 | 0.001292 | 0.014128 | Gm3289 |
| ENSMUSG00000032231 | 1.012601 | 0.001307 | 0.014287 | Anxa2 |
| ENSMUSG00000007617 | 1.630034 | 0.001308 | 0.014288 | Homer1 |
| ENSMUSG00000030930 | -0.83817 | 0.001308 | 0.014288 | Chst15 |
| ENSMUSG00000026986 | -0.84072 | 0.001319 | 0.014396 | Hnmt |
| ENSMUSG00000036030 | 2.519847 | 0.001322 | 0.01442 | Prtg |
| ENSMUSG00000002625 | -0.58628 | 0.001324 | 0.014431 | Akap8l |
| ENSMUSG00000068823 | 0.992969 | 0.001326 | 0.014452 | Csde1 |
| ENSMUSG00000099583 | 2.3415 | 0.00133 | 0.014481 | Hist1h3d |
| ENSMUSG00000031425 | -2.33737 | 0.001338 | 0.014564 | Plp1 |
| ENSMUSG00000036578 | -2.81627 | 0.001341 | 0.014583 | Fxyd7 |
| ENSMUSG00000023902 | -6.53244 | 0.001343 | 0.014593 | Zscan10 |
| ENSMUSG00000013367 | 2.044729 | 0.001343 | 0.014593 | Iglon5 |
| ENSMUSG00000021134 | -0.63354 | 0.001354 | 0.014699 | Srsf5 |
| ENSMUSG00000021037 | 0.73409 | 0.001356 | 0.014721 | Ahsa1 |
| ENSMUSG00000035642 | 1.069355 | 0.00136 | 0.014752 | Aamdc |
| ENSMUSG00000024750 | 1.076911 | 0.001361 | 0.014753 | Zfand5 |
| ENSMUSG00000036067 | 1.699114 | 0.00137 | 0.014845 | Slc2a6 |
| ENSMUSG00000049047 | 0.954475 | 0.001378 | 0.01492 | Armcx3 |
| ENSMUSG00000003721 | 0.656837 | 0.00138 | 0.014933 | Insig2 |
| ENSMUSG00000028367 | 0.648958 | 0.001382 | 0.014948 | Txn1 |
| ENSMUSG00000045237 | 0.797073 | 0.00139 | 0.015027 | 1110012L19Rik |
| ENSMUSG00000028068 | 1.920289 | 0.001392 | 0.015036 | Iqgap3 |
| ENSMUSG00000033953 | 0.790852 | 0.001393 | 0.015036 | Ppp3r1 |
| ENSMUSG00000024587 | 0.564869 | 0.001398 | 0.01509 | Nars |
| ENSMUSG00000041912 | -1.11809 | 0.001413 | 0.015229 | Tdrkh |
| ENSMUSG00000022129 | -2.46957 | 0.001413 | 0.015229 | Dct |
| ENSMUSG00000030591 | 0.706024 | 0.001414 | 0.015237 | Psmd8 |
| ENSMUSG00000038213 | -0.6959 | 0.001419 | 0.015276 | Tapbpl |
| ENSMUSG00000027215 | -0.74404 | 0.00142 | 0.015276 | Cd82 |
| ENSMUSG00000114665 | -3.08728 | 0.001421 | 0.015283 | Gm30918 |
| ENSMUSG00000026730 | 0.961292 | 0.001422 | 0.015287 | Pter |
| ENSMUSG00000022421 | -1.47238 | 0.00143 | 0.015363 | Nptxr |
| ENSMUSG00000029343 | 2.721755 | 0.001439 | 0.015451 | Crybb1 |
| ENSMUSG00000035885 | -1.319 | 0.001442 | 0.015474 | Cox8a |
| ENSMUSG00000026919 | -5.76191 | 0.001444 | 0.015489 | Lcn4 |
| ENSMUSG00000055692 | -1.68013 | 0.001449 | 0.015536 | Tmem191c |
| ENSMUSG00000028445 | 1.417597 | 0.001452 | 0.015557 | Enho |
| ENSMUSG00000060923 | 1.437902 | 0.001454 | 0.015566 | Acyp2 |
| ENSMUSG00000040165 | -2.01389 | 0.001455 | 0.015566 | Cd209c |
| ENSMUSG00000009394 | -1.28479 | 0.001456 | 0.015567 | Syn2 |
| ENSMUSG00000006014 | 1.698782 | 0.001461 | 0.015616 | Prg4 |
| ENSMUSG00000064115 | -2.55146 | 0.001476 | 0.015766 | Cadm2 |
| ENSMUSG00000025574 | 1.64013 | 0.001477 | 0.015766 | Tk1 |
| ENSMUSG00000032301 | 0.682554 | 0.001478 | 0.015776 | Psma4 |
| ENSMUSG00000022416 | -4.61049 | 0.00148 | 0.015788 | Cacna1i |
| ENSMUSG00000048939 | 4.478666 | 0.001484 | 0.015822 | Atp13a5 |
| ENSMUSG00000051986 | -6.95514 | 0.001486 | 0.015829 | A530006G24Rik |
| ENSMUSG00000028745 | 0.679262 | 0.001489 | 0.015855 | Capzb |
| ENSMUSG00000072849 | -2.37344 | 0.001492 | 0.015873 | Serpina1e |
| ENSMUSG00000028072 | -2.43928 | 0.001493 | 0.015874 | Ntrk1 |
| ENSMUSG00000111995 | -6.01171 | 0.001493 | 0.015874 | Gm48697 |
| ENSMUSG00000028601 | -0.92641 | 0.001495 | 0.015874 | Echdc2 |
| ENSMUSG00000015962 | 3.199165 | 0.001495 | 0.015874 | 1700016C15Rik |
| ENSMUSG00000030737 | -1.11646 | 0.001497 | 0.015883 | Slco2b1 |
| ENSMUSG00000022024 | 0.73569 | 0.001498 | 0.015883 | Sugt1 |
| ENSMUSG00000037095 | -1.67538 | 0.001508 | 0.015986 | Lrg1 |
| ENSMUSG00000021973 | 0.60812 | 0.00151 | 0.015994 | Micu2 |
| ENSMUSG00000015340 | 1.720097 | 0.001511 | 0.016001 | Cybb |
| ENSMUSG00000054555 | 1.455352 | 0.001522 | 0.016112 | Adam12 |
| ENSMUSG00000027677 | -0.9416 | 0.001526 | 0.016143 | Ttc14 |
| ENSMUSG00000027579 | -2.66924 | 0.001529 | 0.016162 | Srms |
| ENSMUSG00000097974 | 1.772806 | 0.001533 | 0.016199 | Gm10605 |
| ENSMUSG00000039414 | -0.92155 | 0.001534 | 0.016202 | Heatr5b |
| ENSMUSG00000026100 | 4.757249 | 0.001543 | 0.016287 | Mstn |
| ENSMUSG00000076598 | -6.2116 | 0.001548 | 0.016326 | Igkv3-7 |
| ENSMUSG00000013539 | 0.66977 | 0.001549 | 0.016326 | Tango2 |
| ENSMUSG00000025197 | 3.479064 | 0.001551 | 0.016342 | Cyp2c23 |
| ENSMUSG00000074918 | -0.78001 | 0.001557 | 0.016385 | Inafm2 |
| ENSMUSG00000024302 | 1.922292 | 0.001557 | 0.016385 | Dtna |
| ENSMUSG00000040205 | 5.635038 | 0.001566 | 0.01647 | Cuzd1 |
| ENSMUSG00000003184 | -0.72848 | 0.00158 | 0.016612 | Irf3 |
| ENSMUSG00000020954 | 0.985346 | 0.001583 | 0.016628 | Strn3 |
| ENSMUSG00000034311 | 2.280889 | 0.001584 | 0.016637 | Kif4 |
| ENSMUSG00000042293 | -1.06089 | 0.001587 | 0.01666 | Gm5617 |
| ENSMUSG00000019368 | -2.44762 | 0.001593 | 0.016708 | Sec14l4 |
| ENSMUSG00000027562 | -2.03689 | 0.001597 | 0.016745 | Car2 |
| ENSMUSG00000041548 | 1.233558 | 0.001609 | 0.016859 | Hspb8 |
| ENSMUSG00000021807 | 0.621416 | 0.001616 | 0.01692 | Rtraf |
| ENSMUSG00000113216 | 4.446335 | 0.001617 | 0.016925 | Gm40841 |
| ENSMUSG00000020911 | -2.98565 | 0.001622 | 0.01697 | Krt19 |
| ENSMUSG00000085839 | 2.847319 | 0.001623 | 0.016971 | Gm15949 |
| ENSMUSG00000015127 | -0.95561 | 0.001638 | 0.017112 | Unkl |
| ENSMUSG00000017002 | 2.276764 | 0.001644 | 0.017172 | Slpi |
| ENSMUSG00000097413 | 1.652237 | 0.00165 | 0.017227 | A830052D11Rik |
| ENSMUSG00000028497 | 0.975533 | 0.001654 | 0.017244 | Hacd4 |
| ENSMUSG00000037035 | -1.2773 | 0.001655 | 0.017244 | Inhbb |
| ENSMUSG00000042757 | -3.21539 | 0.001655 | 0.017244 | Tmem108 |
| ENSMUSG00000003949 | 0.967703 | 0.001656 | 0.017244 | Hlf |
| ENSMUSG00000027994 | 1.120926 | 0.00166 | 0.017283 | Mcub |
| ENSMUSG00000035284 | -0.93533 | 0.001667 | 0.017343 | Vps13c |
| ENSMUSG00000022425 | -1.18126 | 0.001668 | 0.017343 | Enpp2 |
| ENSMUSG00000027180 | 0.846897 | 0.001669 | 0.017346 | Fbxo3 |
| ENSMUSG00000022383 | -2.239 | 0.001681 | 0.01746 | Ppara |
| ENSMUSG00000085151 | -1.70091 | 0.001682 | 0.01746 | 1110018N20Rik |
| ENSMUSG00000022358 | 0.983985 | 0.001687 | 0.017502 | Fbxo32 |
| ENSMUSG00000026414 | 2.461595 | 0.00169 | 0.017532 | Tnnt2 |
| ENSMUSG00000024907 | 3.407982 | 0.001693 | 0.017544 | Gal |
| ENSMUSG00000017929 | 0.996614 | 0.001694 | 0.017544 | B4galt5 |
| ENSMUSG00000026021 | 0.69809 | 0.001696 | 0.017562 | Sumo1 |
| ENSMUSG00000061540 | -3.23561 | 0.001697 | 0.017563 | Orm2 |
| ENSMUSG00000054320 | 1.788019 | 0.001701 | 0.017588 | Lrrc36 |
| ENSMUSG00000017639 | -2.10433 | 0.001714 | 0.017709 | Rab11fip4 |
| ENSMUSG00000028179 | -1.50997 | 0.001714 | 0.017709 | Cth |
| ENSMUSG00000039917 | -0.82812 | 0.001718 | 0.017745 | Rhbdd2 |
| ENSMUSG00000038914 | -0.81147 | 0.001724 | 0.017783 | Dido1 |
| ENSMUSG00000024659 | 1.092878 | 0.001724 | 0.017783 | Anxa1 |
| ENSMUSG00000027981 | -0.88964 | 0.001729 | 0.017828 | Rnpc3 |
| ENSMUSG00000047417 | -0.77765 | 0.001731 | 0.017831 | Rexo1 |
| ENSMUSG00000036052 | 1.554251 | 0.001732 | 0.017833 | Dnajb5 |
| ENSMUSG00000026062 | 2.739259 | 0.001734 | 0.017844 | Slc9a2 |
| ENSMUSG00000050315 | 1.772012 | 0.001736 | 0.01786 | Synpo2 |
| ENSMUSG00000029370 | -2.41987 | 0.001747 | 0.017958 | Rassf6 |
| ENSMUSG00000020978 | 0.858933 | 0.001753 | 0.018013 | Klhdc2 |
| ENSMUSG00000019851 | -1.70168 | 0.001759 | 0.018063 | Perp |
| ENSMUSG00000050565 | 0.657157 | 0.001763 | 0.018101 | Tor1aip2 |
| ENSMUSG00000041124 | 0.612165 | 0.001773 | 0.01819 | Msantd4 |
| ENSMUSG00000050982 | -6.87787 | 0.001776 | 0.018211 | Apol10a |
| ENSMUSG00000033579 | -2.04947 | 0.001785 | 0.018292 | Fa2h |
| ENSMUSG00000079363 | -1.24387 | 0.001786 | 0.018292 | Gbp4 |
| ENSMUSG00000057054 | -1.55258 | 0.001788 | 0.018303 | Inca1 |
| ENSMUSG00000022500 | 0.658632 | 0.001793 | 0.01835 | Litaf |
| ENSMUSG00000028772 | 0.60645 | 0.001796 | 0.01836 | Zcchc17 |
| ENSMUSG00000038619 | 0.685864 | 0.001796 | 0.01836 | Ensa |
| ENSMUSG00000070394 | 0.769913 | 0.001799 | 0.018377 | Tmem256 |
| ENSMUSG00000025103 | 0.930421 | 0.001809 | 0.018466 | Btbd1 |
| ENSMUSG00000052423 | -0.88856 | 0.00181 | 0.018466 | B4galt3 |
| ENSMUSG00000020777 | -0.99959 | 0.001819 | 0.018555 | Acox1 |
| ENSMUSG00000096278 | -8.88026 | 0.001822 | 0.01857 | Dcpp2 |
| ENSMUSG00000005198 | -0.68567 | 0.001838 | 0.018724 | Polr2a |
| ENSMUSG00000046215 | -5.18459 | 0.001849 | 0.018828 | Rprml |
| ENSMUSG00000076552 | -6.92017 | 0.00185 | 0.018828 | Igkv4-61 |
| ENSMUSG00000032478 | 1.098769 | 0.001853 | 0.018847 | Nme6 |
| ENSMUSG00000021987 | 0.53149 | 0.001864 | 0.018945 | Mtmr6 |
| ENSMUSG00000023092 | 1.59853 | 0.001864 | 0.018945 | Fhl1 |
| ENSMUSG00000020720 | 0.651067 | 0.001865 | 0.018945 | Psmd12 |
| ENSMUSG00000033849 | -2.06915 | 0.001868 | 0.018955 | B3galt2 |
| ENSMUSG00000050556 | -1.6784 | 0.001868 | 0.018955 | Kcnb1 |
| ENSMUSG00000015950 | 1.33293 | 0.00187 | 0.018966 | Ncf1 |
| ENSMUSG00000108726 | 5.632196 | 0.001874 | 0.018992 | Gm30684 |
| ENSMUSG00000101304 | 1.323539 | 0.001877 | 0.019009 | Plet1os |
| ENSMUSG00000027698 | 1.12454 | 0.001881 | 0.019042 | Nceh1 |
| ENSMUSG00000026567 | -2.57579 | 0.001884 | 0.019066 | Adcy10 |
| ENSMUSG00000050010 | -2.34833 | 0.001887 | 0.019081 | Shisa3 |
| ENSMUSG00000021719 | -1.32733 | 0.001888 | 0.019081 | Rgs7bp |
| ENSMUSG00000027878 | -0.58663 | 0.001897 | 0.019161 | Notch2 |
| ENSMUSG00000025172 | 3.517853 | 0.001898 | 0.019163 | Ankrd2 |
| ENSMUSG00000092564 | -1.08735 | 0.001902 | 0.019195 | BC051226 |
| ENSMUSG00000037994 | 1.705833 | 0.001905 | 0.019216 | Slc9b2 |
| ENSMUSG00000073758 | -1.40492 | 0.001909 | 0.019246 | Sh3d21 |
| ENSMUSG00000109015 | -1.86089 | 0.001912 | 0.019263 | Gm31024 |
| ENSMUSG00000093765 | 3.647368 | 0.001926 | 0.0194 | Gm20658 |
| ENSMUSG00000027387 | 1.171957 | 0.001932 | 0.019446 | Zc3h8 |
| ENSMUSG00000068742 | -0.71087 | 0.001939 | 0.019505 | Cry2 |
| ENSMUSG00000030826 | -1.00138 | 0.001941 | 0.019518 | Bcat2 |
| ENSMUSG00000028093 | -0.97175 | 0.001945 | 0.019546 | Acp6 |
| ENSMUSG00000042978 | -1.18512 | 0.001946 | 0.019546 | Sbk1 |
| ENSMUSG00000067276 | 1.918971 | 0.001956 | 0.019636 | Capn6 |
| ENSMUSG00000108624 | -1.51402 | 0.001957 | 0.019636 | Gm45091 |
| ENSMUSG00000024041 | 4.772255 | 0.001959 | 0.019646 | Cryaa |
| ENSMUSG00000026207 | 1.807837 | 0.00196 | 0.019646 | Speg |
| ENSMUSG00000042717 | 5.518387 | 0.001962 | 0.019657 | Ppp1r3a |
| ENSMUSG00000097368 | 5.332885 | 0.001963 | 0.019657 | Gm10390 |
| ENSMUSG00000076543 | -6.63634 | 0.001972 | 0.019734 | Igkv4-74 |
| ENSMUSG00000002409 | 1.329907 | 0.001986 | 0.019867 | Dyrk1b |
| ENSMUSG00000090063 | -3.84747 | 0.002 | 0.019998 | Dlx6os1 |
| ENSMUSG00000040213 | -1.04422 | 0.002001 | 0.019998 | Kyat3 |
| ENSMUSG00000065592 | -4.48221 | 0.002019 | 0.02016 | Mir145a |
| ENSMUSG00000025766 | -1.09679 | 0.002022 | 0.020169 | D3Ertd751e |
| ENSMUSG00000027488 | 1.60757 | 0.002022 | 0.020169 | Snta1 |
| ENSMUSG00000028457 | -1.794 | 0.002038 | 0.020309 | Atp8b5 |
| ENSMUSG00000030583 | -1.09063 | 0.002038 | 0.020309 | Sipa1l3 |
| ENSMUSG00000024680 | -1.95817 | 0.00204 | 0.020319 | Ms4a2 |
| ENSMUSG00000112352 | -4.22291 | 0.002053 | 0.020442 | Gm40617 |
| ENSMUSG00000044583 | 0.893371 | 0.002066 | 0.020546 | Tlr7 |
| ENSMUSG00000057265 | -1.65204 | 0.002066 | 0.020546 | Bbof1 |
| ENSMUSG00000067106 | -1.40578 | 0.002075 | 0.02063 | Gm5529 |
| ENSMUSG00000032051 | -0.72903 | 0.002084 | 0.020704 | Fdx1 |
| ENSMUSG00000022246 | 0.980742 | 0.002085 | 0.020704 | Rai14 |
| ENSMUSG00000078302 | -5.64489 | 0.002093 | 0.020773 | Foxd1 |
| ENSMUSG00000034903 | -1.20302 | 0.00213 | 0.021124 | Cobll1 |
| ENSMUSG00000025746 | 2.89334 | 0.002131 | 0.021124 | Il6 |
| ENSMUSG00000015016 | -1.05577 | 0.002137 | 0.021171 | Acsf3 |
| ENSMUSG00000055817 | -0.70407 | 0.002138 | 0.021171 | Mta3 |
| ENSMUSG00000031701 | 0.731777 | 0.002142 | 0.021198 | Dnaja2 |
| ENSMUSG00000033186 | 0.801212 | 0.002143 | 0.021198 | Mzt1 |
| ENSMUSG00000041720 | -0.65931 | 0.002144 | 0.021198 | Pi4ka |
| ENSMUSG00000005800 | 2.867484 | 0.002156 | 0.021306 | Mmp8 |
| ENSMUSG00000029821 | 1.070587 | 0.002161 | 0.021353 | Gsdme |
| ENSMUSG00000020067 | 4.918282 | 0.002165 | 0.021375 | Mypn |
| ENSMUSG00000028655 | -3.05615 | 0.00218 | 0.021511 | Mfsd2a |
| ENSMUSG00000028427 | -2.27098 | 0.002182 | 0.021511 | Aqp7 |
| ENSMUSG00000031386 | -0.56909 | 0.002183 | 0.021511 | Hcfc1 |
| ENSMUSG00000109282 | -3.72407 | 0.002183 | 0.021511 | Gm45188 |
| ENSMUSG00000034022 | -0.83216 | 0.002184 | 0.021511 | Cpsf1 |
| ENSMUSG00000097092 | -5.5375 | 0.002188 | 0.021533 | Gm26725 |
| ENSMUSG00000057092 | -2.88422 | 0.002192 | 0.021567 | Fxyd3 |
| ENSMUSG00000050578 | 2.963407 | 0.002195 | 0.021587 | Mmp13 |
| ENSMUSG00000027171 | -1.88078 | 0.002196 | 0.021587 | Prrg4 |
| ENSMUSG00000027018 | 0.744467 | 0.002204 | 0.021649 | Hat1 |
| ENSMUSG00000047497 | 0.999543 | 0.002216 | 0.021754 | Adamts12 |
| ENSMUSG00000043903 | 1.889375 | 0.002222 | 0.021802 | Zfp469 |
| ENSMUSG00000109724 | 1.119162 | 0.002223 | 0.021802 | Gm18194 |
| ENSMUSG00000006641 | -1.19871 | 0.002229 | 0.021852 | Slc5a6 |
| ENSMUSG00000109695 | 2.646735 | 0.002233 | 0.021879 | Gm31166 |
| ENSMUSG00000062488 | 1.839499 | 0.002235 | 0.021892 | Ifit3b |
| ENSMUSG00000000739 | -1.12794 | 0.002245 | 0.021979 | Sult5a1 |
| ENSMUSG00000032475 | 0.62544 | 0.00225 | 0.022016 | Nck1 |
| ENSMUSG00000033773 | 0.864586 | 0.002255 | 0.022045 | Rpap2 |
| ENSMUSG00000070923 | 0.5895 | 0.002264 | 0.022122 | Klhl9 |
| ENSMUSG00000047694 | -0.62003 | 0.002269 | 0.022161 | Yipf6 |
| ENSMUSG00000060935 | 0.841246 | 0.002273 | 0.022193 | Tmem263 |
| ENSMUSG00000013878 | 0.606499 | 0.002287 | 0.022313 | Rnf170 |
| ENSMUSG00000050357 | -3.15294 | 0.0023 | 0.022429 | Carmil2 |
| ENSMUSG00000025352 | 1.231116 | 0.002302 | 0.022441 | Gdf11 |
| ENSMUSG00000039105 | 0.564388 | 0.002311 | 0.02251 | Atp6v1g1 |
| ENSMUSG00000027469 | 1.543517 | 0.002312 | 0.02251 | Tpx2 |
| ENSMUSG00000038482 | 0.552482 | 0.002315 | 0.02253 | Tfdp1 |
| ENSMUSG00000042045 | 5.894142 | 0.002318 | 0.022545 | Sln |
| ENSMUSG00000039419 | 3.485513 | 0.002319 | 0.022545 | Cntnap2 |
| ENSMUSG00000037995 | -1.26143 | 0.002321 | 0.022545 | Igsf9 |
| ENSMUSG00000074219 | -3.37864 | 0.002321 | 0.022545 | Gm10644 |
| ENSMUSG00000000486 | -2.5145 | 0.002334 | 0.022662 | 1-Sep |
| ENSMUSG00000026683 | 1.908526 | 0.002337 | 0.022672 | Nuf2 |
| ENSMUSG00000052387 | -1.68714 | 0.002338 | 0.022672 | Trpm3 |
| ENSMUSG00000048878 | -0.73002 | 0.002341 | 0.022683 | Hexim1 |
| ENSMUSG00000014602 | -2.68365 | 0.002341 | 0.022683 | Kif1a |
| ENSMUSG00000032363 | 1.417438 | 0.002343 | 0.022684 | Adamts7 |
| ENSMUSG00000021536 | 1.978014 | 0.002344 | 0.022684 | Adcy2 |
| ENSMUSG00000005320 | 3.054599 | 0.002352 | 0.022752 | Fgfr4 |
| ENSMUSG00000041313 | -0.85834 | 0.002353 | 0.022752 | Slc7a1 |
| ENSMUSG00000049739 | -0.75781 | 0.002356 | 0.022762 | Zfp646 |
| ENSMUSG00000027984 | -1.31055 | 0.002357 | 0.022762 | Hadh |
| ENSMUSG00000021990 | -1.43497 | 0.002358 | 0.022762 | Spata13 |
| ENSMUSG00000024507 | -0.84106 | 0.002359 | 0.022765 | Hsd17b4 |
| ENSMUSG00000043687 | -3.46752 | 0.002364 | 0.022799 | 1190005I06Rik |
| ENSMUSG00000027536 | -1.72358 | 0.002373 | 0.022878 | Chmp4c |
| ENSMUSG00000026023 | 2.282206 | 0.002388 | 0.023005 | Cdk15 |
| ENSMUSG00000039519 | 1.13508 | 0.002397 | 0.023083 | Cyp7b1 |
| ENSMUSG00000078452 | 2.100933 | 0.002403 | 0.023131 | Raet1d |
| ENSMUSG00000083844 | 0.723717 | 0.002413 | 0.023216 | Ube2d-ps |
| ENSMUSG00000042401 | -3.76193 | 0.002419 | 0.023262 | Crtac1 |
| ENSMUSG00000031111 | -3.06784 | 0.002423 | 0.023291 | Igsf1 |
| ENSMUSG00000090086 | -1.13076 | 0.002425 | 0.023295 | AI480526 |
| ENSMUSG00000035352 | 2.186816 | 0.002434 | 0.023369 | Ccl12 |
| ENSMUSG00000056328 | 4.56339 | 0.002435 | 0.023371 | Myh1 |
| ENSMUSG00000043467 | -0.96055 | 0.002439 | 0.023398 | Zbtb37 |
| ENSMUSG00000028865 | -3.03656 | 0.002451 | 0.023499 | Cd164l2 |
| ENSMUSG00000038147 | 1.626902 | 0.002463 | 0.023602 | Cd84 |
| ENSMUSG00000086784 | -0.88067 | 0.002464 | 0.023602 | Isoc2a |
| ENSMUSG00000017716 | 2.095827 | 0.002466 | 0.023602 | Birc5 |
| ENSMUSG00000042408 | -0.67759 | 0.002467 | 0.023606 | Zmym6 |
| ENSMUSG00000004043 | -0.94627 | 0.00247 | 0.023625 | Stat5a |
| ENSMUSG00000115230 | 3.040999 | 0.002473 | 0.023643 | AU022793 |
| ENSMUSG00000030852 | 1.328685 | 0.002476 | 0.023655 | Tacc2 |
| ENSMUSG00000024537 | 0.65131 | 0.002479 | 0.023673 | Psmg2 |
| ENSMUSG00000061666 | 1.01268 | 0.00248 | 0.023673 | Gdpd1 |
| ENSMUSG00000030291 | 0.854506 | 0.002486 | 0.023712 | Med21 |
| ENSMUSG00000062797 | 0.801741 | 0.002488 | 0.023718 | Hikeshi |
| ENSMUSG00000105606 | -4.98817 | 0.002505 | 0.023869 | Igkv2-109 |
| ENSMUSG00000023047 | 2.350435 | 0.002507 | 0.023875 | Amhr2 |
| ENSMUSG00000056054 | 2.14669 | 0.002509 | 0.023875 | S100a8 |
| ENSMUSG00000031618 | -0.78005 | 0.002509 | 0.023875 | Nr3c2 |
| ENSMUSG00000032625 | -1.54734 | 0.00252 | 0.023972 | Thsd7a |
| ENSMUSG00000000276 | -0.91604 | 0.002522 | 0.023973 | Dgke |
| ENSMUSG00000085925 | 4.788165 | 0.002524 | 0.023982 | Rtl1 |
| ENSMUSG00000009549 | 0.712082 | 0.002529 | 0.024016 | Srp14 |
| ENSMUSG00000042246 | 1.530603 | 0.002534 | 0.024055 | Tmc7 |
| ENSMUSG00000000916 | -0.95197 | 0.002543 | 0.024128 | Nsun5 |
| ENSMUSG00000001642 | 0.652456 | 0.002548 | 0.024155 | Akr1b3 |
| ENSMUSG00000048078 | -1.70889 | 0.00255 | 0.024155 | Tenm4 |
| ENSMUSG00000050144 | -1.07379 | 0.00255 | 0.024155 | Slc25a44 |
| ENSMUSG00000038349 | 1.028716 | 0.002551 | 0.024155 | Plcl1 |
| ENSMUSG00000020623 | 1.462686 | 0.002554 | 0.024175 | Map2k6 |
| ENSMUSG00000072941 | -1.31724 | 0.002559 | 0.024203 | Sod3 |
| ENSMUSG00000032803 | 0.802967 | 0.00257 | 0.024293 | Cdv3 |
| ENSMUSG00000006638 | -3.02413 | 0.002571 | 0.024293 | Abhd1 |
| ENSMUSG00000044068 | -0.78315 | 0.002572 | 0.024294 | Zrsr1 |
| ENSMUSG00000009535 | 0.917279 | 0.002576 | 0.02431 | Rnmt |
| ENSMUSG00000025138 | -0.87732 | 0.002576 | 0.02431 | Sirt7 |
| ENSMUSG00000036427 | 1.001116 | 0.002578 | 0.024311 | Gpi1 |
| ENSMUSG00000095789 | -1.43384 | 0.002584 | 0.024355 | Nupr1l |
| ENSMUSG00000100615 | -5.90762 | 0.002598 | 0.02448 | Gm5511 |
| ENSMUSG00000030315 | -0.63741 | 0.002603 | 0.024514 | Vgll4 |
| ENSMUSG00000083179 | 1.276303 | 0.002615 | 0.024619 | Gm12693 |
| ENSMUSG00000022554 | -1.03418 | 0.00263 | 0.024748 | Hgh1 |
| ENSMUSG00000018927 | 1.299077 | 0.002636 | 0.024784 | Ccl6 |
| ENSMUSG00000025170 | 1.169602 | 0.002645 | 0.024857 | Rab40b |
| ENSMUSG00000019797 | 0.814861 | 0.002647 | 0.024857 | 1700021F05Rik |
| ENSMUSG00000037754 | -1.24498 | 0.002647 | 0.024857 | Ppp1r16b |
| ENSMUSG00000019210 | 0.585821 | 0.002648 | 0.024857 | Atp6v1e1 |
| ENSMUSG00000063889 | 0.687052 | 0.002661 | 0.024955 | Crem |
| ENSMUSG00000032556 | -3.86542 | 0.002662 | 0.024955 | Bfsp2 |
| ENSMUSG00000038943 | 1.801454 | 0.002663 | 0.024955 | Prc1 |
| ENSMUSG00000029661 | 1.250058 | 0.00267 | 0.025011 | Col1a2 |
| ENSMUSG00000040229 | 1.0841 | 0.002672 | 0.025013 | Gpr34 |
| ENSMUSG00000052593 | 0.52322 | 0.002677 | 0.025047 | Adam17 |
| ENSMUSG00000046694 | -1.78332 | 0.002683 | 0.025094 | Tent5b |
| ENSMUSG00000097289 | 5.513855 | 0.002686 | 0.025099 | 2010300F17Rik |
| ENSMUSG00000058975 | 2.482375 | 0.002686 | 0.025099 | Kcnc1 |
| ENSMUSG00000022394 | -0.84522 | 0.002695 | 0.025173 | L3mbtl2 |
| ENSMUSG00000017999 | -0.64456 | 0.002698 | 0.025189 | Ddx27 |
| ENSMUSG00000036281 | -0.79842 | 0.0027 | 0.025191 | Snapc4 |
| ENSMUSG00000109868 | -5.55221 | 0.002702 | 0.025202 | Gm39244 |
| ENSMUSG00000031767 | -1.3508 | 0.002709 | 0.025238 | Nudt7 |
| ENSMUSG00000036986 | -0.69545 | 0.002709 | 0.025238 | Pml |
| ENSMUSG00000081752 | 1.618607 | 0.00271 | 0.025238 | Sms-ps |
| ENSMUSG00000033152 | 1.517134 | 0.002722 | 0.025333 | Podxl2 |
| ENSMUSG00000057337 | -2.6983 | 0.002723 | 0.025333 | Chst3 |
| ENSMUSG00000033099 | 0.586614 | 0.002732 | 0.0254 | Nol12 |
| ENSMUSG00000051166 | -1.54746 | 0.002744 | 0.025502 | Eml5 |
| ENSMUSG00000026548 | 1.272637 | 0.002746 | 0.025511 | Slamf9 |
| ENSMUSG00000039691 | -5.63132 | 0.002752 | 0.025552 | Tspan10 |
| ENSMUSG00000003283 | 1.413836 | 0.002755 | 0.025566 | Hck |
| ENSMUSG00000032434 | -0.69065 | 0.002781 | 0.025802 | Cmtm6 |
| ENSMUSG00000047344 | -2.99661 | 0.002788 | 0.025855 | Lancl3 |
| ENSMUSG00000000094 | 3.600823 | 0.002806 | 0.026005 | Tbx4 |
| ENSMUSG00000031708 | -0.93049 | 0.002811 | 0.026041 | Tecr |
| ENSMUSG00000028373 | -1.96966 | 0.002817 | 0.026077 | Astn2 |
| ENSMUSG00000049686 | 0.840281 | 0.002818 | 0.026077 | Orai1 |
| ENSMUSG00000027978 | 1.167441 | 0.002835 | 0.026219 | Prss12 |
| ENSMUSG00000062563 | 1.451364 | 0.00285 | 0.026352 | Cys1 |
| ENSMUSG00000020184 | 0.720824 | 0.002858 | 0.026412 | Mdm2 |
| ENSMUSG00000048787 | -0.7162 | 0.00288 | 0.0266 | Dcun1d3 |
| ENSMUSG00000032492 | -1.0447 | 0.002883 | 0.026608 | Pth1r |
| ENSMUSG00000025473 | 1.997562 | 0.002884 | 0.026608 | Adam8 |
| ENSMUSG00000052336 | 1.529296 | 0.002887 | 0.026627 | Cx3cr1 |
| ENSMUSG00000034308 | -1.13923 | 0.002889 | 0.02663 | Sdr42e1 |
| ENSMUSG00000003559 | 0.961812 | 0.002892 | 0.026648 | As3mt |
| ENSMUSG00000103088 | -1.55971 | 0.002909 | 0.026788 | Pcdhgb6 |
| ENSMUSG00000049281 | -1.72109 | 0.002916 | 0.026841 | Scn3b |
| ENSMUSG00000024947 | -0.74592 | 0.002924 | 0.026906 | Men1 |
| ENSMUSG00000022816 | 1.337044 | 0.002941 | 0.027049 | Fstl1 |
| ENSMUSG00000022748 | 1.134111 | 0.002958 | 0.027186 | Cmss1 |
| ENSMUSG00000074994 | 0.907109 | 0.00296 | 0.027198 | Qser1 |
| ENSMUSG00000039361 | 0.513922 | 0.002963 | 0.027206 | Picalm |
| ENSMUSG00000031431 | 1.04071 | 0.002965 | 0.027216 | Tsc22d3 |
| ENSMUSG00000066861 | 1.493703 | 0.002969 | 0.027217 | Oas1g |
| ENSMUSG00000031924 | -0.83539 | 0.002969 | 0.027217 | Cyb5b |
| ENSMUSG00000095028 | 1.600129 | 0.00297 | 0.027217 | Sirpb1b |
| ENSMUSG00000026828 | 2.536707 | 0.002974 | 0.027244 | Galnt5 |
| ENSMUSG00000043110 | -5.02318 | 0.002998 | 0.027454 | Lrrn4 |
| ENSMUSG00000022982 | -0.84888 | 0.003007 | 0.027519 | Sod1 |
| ENSMUSG00000041187 | -0.95774 | 0.003025 | 0.027673 | Prkd2 |
| ENSMUSG00000027399 | 2.345311 | 0.00303 | 0.027709 | Il1a |
| ENSMUSG00000051839 | -4.31161 | 0.003034 | 0.027728 | Gypa |
| ENSMUSG00000044600 | 0.55411 | 0.003037 | 0.027734 | Smim7 |
| ENSMUSG00000049001 | 1.226957 | 0.003037 | 0.027734 | Ndnf |
| ENSMUSG00000037791 | -0.6435 | 0.00304 | 0.027746 | Phf12 |
| ENSMUSG00000005615 | 0.838945 | 0.003045 | 0.027776 | Pcyt1a |
| ENSMUSG00000030004 | 3.197081 | 0.003054 | 0.027844 | Nat8 |
| ENSMUSG00000041729 | -0.88494 | 0.003056 | 0.027848 | Coro2b |
| ENSMUSG00000058492 | -1.00638 | 0.003059 | 0.027861 | Scp2-ps2 |
| ENSMUSG00000036934 | 0.679675 | 0.00306 | 0.027861 | 4921524J17Rik |
| ENSMUSG00000022949 | -2.503 | 0.003078 | 0.027998 | Clic6 |
| ENSMUSG00000027287 | 0.583936 | 0.003078 | 0.027998 | Snap23 |
| ENSMUSG00000108723 | 4.7883 | 0.003083 | 0.028016 | Gm44837 |
| ENSMUSG00000021771 | 0.737658 | 0.003083 | 0.028016 | Vdac2 |
| ENSMUSG00000041638 | -0.67311 | 0.003091 | 0.02808 | Gcn1l1 |
| ENSMUSG00000096150 | -6.94112 | 0.003095 | 0.028088 | Ighv1-85 |
| ENSMUSG00000047696 | -2.7156 | 0.003095 | 0.028088 | Ccdc144b |
| ENSMUSG00000031137 | 1.443953 | 0.003103 | 0.028143 | Fgf13 |
| ENSMUSG00000040658 | 1.487223 | 0.003109 | 0.028188 | Dnph1 |
| ENSMUSG00000022490 | -1.72466 | 0.003127 | 0.028329 | Ppp1r1a |
| ENSMUSG00000002108 | -0.92483 | 0.003128 | 0.028329 | Nr1h3 |
| ENSMUSG00000095362 | 0.613613 | 0.003143 | 0.028454 | Gm14325 |
| ENSMUSG00000022534 | 2.141207 | 0.003155 | 0.028531 | Mefv |
| ENSMUSG00000112148 | 1.679617 | 0.003156 | 0.028531 | Lilrb4a |
| ENSMUSG00000032458 | 0.601004 | 0.003156 | 0.028531 | Copb2 |
| ENSMUSG00000043644 | 1.132029 | 0.003166 | 0.028605 | 0610009L18Rik |
| ENSMUSG00000054978 | 1.222247 | 0.003181 | 0.028729 | Kbtbd13 |
| ENSMUSG00000015354 | -1.55465 | 0.003184 | 0.028745 | Pcolce2 |
| ENSMUSG00000037972 | -1.09145 | 0.003201 | 0.028862 | Snn |
| ENSMUSG00000076608 | -5.83647 | 0.003201 | 0.028862 | Igkj5 |
| ENSMUSG00000097203 | -2.94069 | 0.003202 | 0.028862 | 4732419C18Rik |
| ENSMUSG00000046160 | -4.23537 | 0.003211 | 0.028928 | Olig1 |
| ENSMUSG00000065037 | 2.260476 | 0.003238 | 0.029162 | Rn7sk |
| ENSMUSG00000036863 | -0.97042 | 0.003247 | 0.029233 | Syde2 |
| ENSMUSG00000001999 | 0.640683 | 0.003259 | 0.029319 | Blvra |
| ENSMUSG00000030732 | 5.588616 | 0.003275 | 0.02945 | Chrdl2 |
| ENSMUSG00000021892 | -0.5925 | 0.003286 | 0.029537 | Sh3bp5 |
| ENSMUSG00000041168 | -0.68352 | 0.00332 | 0.029827 | Lonp1 |
| ENSMUSG00000056888 | 1.860125 | 0.003321 | 0.029827 | Glipr1 |
| ENSMUSG00000033209 | -1.14718 | 0.003332 | 0.029914 | Ttc28 |
| ENSMUSG00000025534 | 0.660804 | 0.003335 | 0.029923 | Gusb |
| ENSMUSG00000069806 | 1.214056 | 0.003348 | 0.030026 | Cacng7 |
| ENSMUSG00000021109 | 0.722931 | 0.00335 | 0.030026 | Hif1a |
| ENSMUSG00000024186 | -1.52296 | 0.003355 | 0.030059 | Rgs11 |
| ENSMUSG00000007891 | 0.698166 | 0.003359 | 0.030085 | Ctsd |
| ENSMUSG00000040356 | -0.616 | 0.003366 | 0.030132 | Skiv2l |
| ENSMUSG00000036112 | 0.66966 | 0.003395 | 0.030376 | Metap2 |
| ENSMUSG00000076606 | -6.9466 | 0.003398 | 0.030389 | Igkj3 |
| ENSMUSG00000022044 | -1.27225 | 0.003405 | 0.030424 | Stmn4 |
| ENSMUSG00000038422 | -1.39708 | 0.003407 | 0.030424 | Hdhd3 |
| ENSMUSG00000036123 | -2.94966 | 0.003407 | 0.030424 | Slc9a3 |
| ENSMUSG00000066595 | 0.742995 | 0.003408 | 0.030424 | Flvcr1 |
| ENSMUSG00000029119 | -1.00503 | 0.00342 | 0.030517 | Man2b2 |
| ENSMUSG00000043088 | -1.8793 | 0.003423 | 0.030523 | Il17re |
| ENSMUSG00000091712 | 1.774497 | 0.003429 | 0.030562 | Sec14l5 |
| ENSMUSG00000052396 | -2.9541 | 0.003431 | 0.030567 | Mogat2 |
| ENSMUSG00000030041 | 1.409138 | 0.003435 | 0.030567 | M1ap |
| ENSMUSG00000008333 | 0.719021 | 0.003435 | 0.030567 | Snrpb2 |
| ENSMUSG00000026107 | -1.24883 | 0.003436 | 0.030567 | Nabp1 |
| ENSMUSG00000022131 | 0.594774 | 0.003437 | 0.030567 | Gpr180 |
| ENSMUSG00000040820 | -0.79558 | 0.003443 | 0.030604 | Hlcs |
| ENSMUSG00000037664 | 1.315008 | 0.003446 | 0.030615 | Cdkn1c |
| ENSMUSG00000019659 | 0.727064 | 0.00346 | 0.030728 | Ccdc12 |
| ENSMUSG00000037772 | 0.560768 | 0.003476 | 0.03085 | Mrpl23 |
| ENSMUSG00000021287 | -1.15251 | 0.003477 | 0.03085 | Xrcc3 |
| ENSMUSG00000023995 | -5.12528 | 0.003483 | 0.030892 | Tspo2 |
| ENSMUSG00000032381 | 0.807254 | 0.003493 | 0.03096 | Fam96a |
| ENSMUSG00000078521 | 3.398017 | 0.003499 | 0.030999 | Aunip |
| ENSMUSG00000038344 | -1.00416 | 0.003513 | 0.031115 | Txlng |
| ENSMUSG00000019782 | 0.739453 | 0.003515 | 0.031115 | Rwdd1 |
| ENSMUSG00000107768 | 5.510151 | 0.003518 | 0.031128 | Gm44275 |
| ENSMUSG00000025040 | 0.575817 | 0.00352 | 0.031128 | Fundc1 |
| ENSMUSG00000053964 | -0.93994 | 0.003528 | 0.031187 | Lgals4 |
| ENSMUSG00000018848 | 0.651424 | 0.003542 | 0.031299 | Rars |
| ENSMUSG00000032802 | 0.900129 | 0.003561 | 0.031439 | Srxn1 |
| ENSMUSG00000040528 | 1.154534 | 0.003564 | 0.031439 | Milr1 |
| ENSMUSG00000027163 | 0.613157 | 0.003565 | 0.031439 | Commd9 |
| ENSMUSG00000021094 | -0.85965 | 0.003565 | 0.031439 | Dhrs7 |
| ENSMUSG00000006462 | 1.411364 | 0.003575 | 0.031512 | A530013C23Rik |
| ENSMUSG00000024601 | -0.66931 | 0.003589 | 0.031622 | Isoc1 |
| ENSMUSG00000109341 | 2.903656 | 0.003592 | 0.031633 | Gm30873 |
| ENSMUSG00000029312 | 1.024377 | 0.003601 | 0.031698 | Klhl8 |
| ENSMUSG00000078648 | 4.680548 | 0.003606 | 0.031726 | Gm17546 |
| ENSMUSG00000097715 | 0.82809 | 0.003613 | 0.031765 | Gpr137b-ps |
| ENSMUSG00000037259 | -1.56229 | 0.003613 | 0.031765 | Dzank1 |
| ENSMUSG00000095609 | 1.064067 | 0.003626 | 0.031867 | Gm21188 |
| ENSMUSG00000003778 | -0.60375 | 0.003633 | 0.031908 | Brd8 |
| ENSMUSG00000032081 | -3.1666 | 0.003649 | 0.032033 | Apoc3 |
| ENSMUSG00000073940 | -4.68983 | 0.003665 | 0.032147 | Hbb-bt |
| ENSMUSG00000015714 | -0.74428 | 0.003665 | 0.032147 | Cers2 |
| ENSMUSG00000021178 | 0.556452 | 0.003691 | 0.03236 | Psmc1 |
| ENSMUSG00000089783 | -2.08167 | 0.003694 | 0.03237 | Gm454 |
| ENSMUSG00000114608 | 0.963692 | 0.003724 | 0.032604 | Gm36161 |
| ENSMUSG00000022885 | -0.74675 | 0.003726 | 0.032604 | St6gal1 |
| ENSMUSG00000029780 | 0.891042 | 0.003726 | 0.032604 | Nt5c3 |
| ENSMUSG00000087362 | 2.957141 | 0.003727 | 0.032604 | Gm13710 |
| ENSMUSG00000016529 | 1.885305 | 0.003731 | 0.032623 | Il10 |
| ENSMUSG00000015745 | 0.802426 | 0.003739 | 0.032674 | Plekho1 |
| ENSMUSG00000101972 | 4.646874 | 0.003744 | 0.032706 | Hist1h3i |
| ENSMUSG00000031710 | -7.62226 | 0.003749 | 0.032735 | Ucp1 |
| ENSMUSG00000049037 | 1.087386 | 0.003754 | 0.032766 | Clec4a1 |
| ENSMUSG00000087505 | -6.48411 | 0.003756 | 0.032766 | Gm15241 |
| ENSMUSG00000024132 | -1.28187 | 0.003773 | 0.032899 | Eci1 |
| ENSMUSG00000111954 | -1.99704 | 0.003781 | 0.032951 | Gm7001 |
| ENSMUSG00000069515 | 1.343591 | 0.003783 | 0.032959 | Lyz1 |
| ENSMUSG00000022475 | -1.08501 | 0.003786 | 0.032969 | Hdac7 |
| ENSMUSG00000026271 | 1.415885 | 0.003811 | 0.033171 | Gpr35 |
| ENSMUSG00000018648 | 1.021435 | 0.003826 | 0.033283 | Dusp14 |
| ENSMUSG00000092341 | -0.819 | 0.003835 | 0.033351 | Malat1 |
| ENSMUSG00000097754 | 1.279596 | 0.003848 | 0.033445 | Ptgs2os2 |
| ENSMUSG00000060376 | -0.95316 | 0.003871 | 0.033616 | Bckdha |
| ENSMUSG00000028156 | 0.776875 | 0.003871 | 0.033616 | Eif4e |
| ENSMUSG00000044916 | -1.49155 | 0.003891 | 0.033776 | 1700029I15Rik |
| ENSMUSG00000022498 | -0.65464 | 0.003894 | 0.033786 | Txndc11 |
| ENSMUSG00000027776 | 2.326705 | 0.003909 | 0.033891 | Il12a |
| ENSMUSG00000052302 | -1.54953 | 0.00391 | 0.033891 | Tbc1d30 |
| ENSMUSG00000058793 | -0.75173 | 0.003918 | 0.033946 | Cds2 |
| ENSMUSG00000059263 | 0.8196 | 0.003921 | 0.033955 | Usp47 |
| ENSMUSG00000064202 | -1.43326 | 0.003925 | 0.03398 | 4430402I18Rik |
| ENSMUSG00000030609 | 0.788871 | 0.003929 | 0.033993 | Aen |
| ENSMUSG00000020303 | -1.63202 | 0.003938 | 0.034062 | Stc2 |
| ENSMUSG00000023079 | -0.78135 | 0.00396 | 0.034234 | Gtf2ird1 |
| ENSMUSG00000028992 | 0.984335 | 0.003962 | 0.034236 | Nmnat1 |
| ENSMUSG00000040724 | -2.07986 | 0.003972 | 0.034309 | Kcna2 |
| ENSMUSG00000010529 | 1.358908 | 0.003977 | 0.034333 | Gm266 |
| ENSMUSG00000025261 | -0.63186 | 0.00398 | 0.034345 | Huwe1 |
| ENSMUSG00000028470 | -0.98088 | 0.003996 | 0.034467 | Hint2 |
| ENSMUSG00000085322 | 5.218302 | 0.004001 | 0.034483 | Gm14261 |
| ENSMUSG00000029638 | -2.25854 | 0.004005 | 0.034483 | Glcci1 |
| ENSMUSG00000031805 | -0.90686 | 0.004005 | 0.034483 | Jak3 |
| ENSMUSG00000016534 | 0.781961 | 0.004005 | 0.034483 | Lamp2 |
| ENSMUSG00000033526 | -0.79381 | 0.00401 | 0.034507 | Ppip5k1 |
| ENSMUSG00000026475 | 2.156272 | 0.004014 | 0.034532 | Rgs16 |
| ENSMUSG00000086918 | 2.142022 | 0.004025 | 0.034609 | 4930429F24Rik |
| ENSMUSG00000044250 | -1.62968 | 0.004028 | 0.034621 | Pced1b |
| ENSMUSG00000021022 | 0.67102 | 0.00403 | 0.034621 | Ppp2r3c |
| ENSMUSG00000041126 | 0.609867 | 0.004034 | 0.034637 | H2afv |
| ENSMUSG00000051341 | 0.706402 | 0.004053 | 0.034788 | Zfp52 |
| ENSMUSG00000069670 | -3.5067 | 0.004077 | 0.034975 | Nkain2 |
| ENSMUSG00000034161 | 1.550794 | 0.004084 | 0.035017 | Scx |
| ENSMUSG00000060090 | 0.70377 | 0.004085 | 0.035017 | Rp2 |
| ENSMUSG00000049721 | -2.69307 | 0.00409 | 0.035041 | Gal3st1 |
| ENSMUSG00000086938 | -1.24147 | 0.004096 | 0.035068 | 4930481A15Rik |
| ENSMUSG00000029028 | -0.82258 | 0.004097 | 0.035068 | Lrrc47 |
| ENSMUSG00000080662 | 5.229496 | 0.0041 | 0.035079 | Mir1b |
| ENSMUSG00000036492 | -1.80592 | 0.004111 | 0.035148 | Rnf39 |
| ENSMUSG00000057897 | 1.387622 | 0.004112 | 0.035148 | Camk2b |
| ENSMUSG00000020387 | -0.62614 | 0.004121 | 0.035215 | Jade2 |
| ENSMUSG00000031661 | -1.45361 | 0.004125 | 0.035228 | Nkd1 |
| ENSMUSG00000097187 | -1.67448 | 0.004127 | 0.035228 | Gm19426 |
| ENSMUSG00000053040 | 1.091797 | 0.00413 | 0.035243 | Aph1c |
| ENSMUSG00000105961 | 5.384478 | 0.004134 | 0.035258 | Gm40123 |
| ENSMUSG00000022951 | 0.879857 | 0.00415 | 0.035377 | Rcan1 |
| ENSMUSG00000030499 | 1.187271 | 0.004164 | 0.035484 | Kctd15 |
| ENSMUSG00000032232 | -1.09256 | 0.004168 | 0.0355 | Cgnl1 |
| ENSMUSG00000010830 | 1.093521 | 0.00417 | 0.0355 | Kdelr3 |
| ENSMUSG00000104839 | -6.52629 | 0.004189 | 0.035653 | Gm42794 |
| ENSMUSG00000045180 | -1.75918 | 0.004211 | 0.035823 | Shroom2 |
| ENSMUSG00000073380 | -4.46339 | 0.004214 | 0.035834 | Arrdc5 |
| ENSMUSG00000074472 | -2.12935 | 0.004218 | 0.03585 | Zfp872 |
| ENSMUSG00000013419 | 1.1035 | 0.004229 | 0.035925 | Zfp651 |
| ENSMUSG00000033335 | -0.55422 | 0.004232 | 0.035938 | Dnm2 |
| ENSMUSG00000045775 | -3.00147 | 0.004243 | 0.036016 | Slc16a5 |
| ENSMUSG00000020089 | -1.05672 | 0.004249 | 0.036049 | Ppa1 |
| ENSMUSG00000096225 | -0.95885 | 0.004282 | 0.036286 | Lhx8 |
| ENSMUSG00000075705 | 1.095854 | 0.004282 | 0.036286 | Msrb1 |
| ENSMUSG00000023176 | -3.74887 | 0.004283 | 0.036286 | Cpn2 |
| ENSMUSG00000097125 | 4.291751 | 0.004304 | 0.036442 | Gm26885 |
| ENSMUSG00000046610 | 3.671875 | 0.004306 | 0.036442 | Oacyl |
| ENSMUSG00000110185 | 0.781659 | 0.004309 | 0.036442 | Igip |
| ENSMUSG00000043613 | 1.509787 | 0.004309 | 0.036442 | Mmp3 |
| ENSMUSG00000018567 | 0.577199 | 0.004313 | 0.036459 | Gabarap |
| ENSMUSG00000020704 | -5.36986 | 0.004319 | 0.036495 | Asic2 |
| ENSMUSG00000029348 | -1.32457 | 0.00433 | 0.036572 | Asphd2 |
| ENSMUSG00000066154 | -2.25887 | 0.004336 | 0.036602 | Mup3 |
| ENSMUSG00000000197 | -2.2911 | 0.004337 | 0.036602 | Nalcn |
| ENSMUSG00000049303 | -1.63104 | 0.004346 | 0.036657 | Syt12 |
| ENSMUSG00000002944 | -1.28457 | 0.004349 | 0.036668 | Cd36 |
| ENSMUSG00000057596 | 0.782108 | 0.004354 | 0.036693 | Trim30d |
| ENSMUSG00000029446 | -0.82939 | 0.004356 | 0.036698 | Psph |
| ENSMUSG00000025268 | 1.303244 | 0.00436 | 0.036711 | Maged2 |
| ENSMUSG00000074656 | 0.739639 | 0.004365 | 0.036726 | Eif2s2 |
| ENSMUSG00000034853 | -1.41081 | 0.004365 | 0.036726 | Acot11 |
| ENSMUSG00000029379 | 4.674665 | 0.004368 | 0.036729 | Cxcl3 |
| ENSMUSG00000022801 | -0.94705 | 0.00437 | 0.036734 | Lrch3 |
| ENSMUSG00000026697 | 1.681621 | 0.004376 | 0.036764 | Myoc |
| ENSMUSG00000037005 | -1.30425 | 0.004395 | 0.036912 | Xpnpep2 |
| ENSMUSG00000054793 | -1.97292 | 0.00441 | 0.037022 | Cadm4 |
| ENSMUSG00000027495 | -0.90367 | 0.004432 | 0.037191 | Fam210b |
| ENSMUSG00000034121 | -1.05848 | 0.004437 | 0.037213 | Mks1 |
| ENSMUSG00000024969 | -0.61783 | 0.004442 | 0.037232 | Mark2 |
| ENSMUSG00000037364 | -0.62589 | 0.004443 | 0.037232 | Srrt |
| ENSMUSG00000061244 | 0.496641 | 0.004453 | 0.037298 | Exoc5 |
| ENSMUSG00000008450 | 0.588053 | 0.004464 | 0.037378 | Nutf2 |
| ENSMUSG00000018166 | -1.53805 | 0.004469 | 0.037401 | Erbb3 |
| ENSMUSG00000020792 | 0.838427 | 0.004476 | 0.037444 | Exoc7 |
| ENSMUSG00000024231 | 0.794105 | 0.004482 | 0.037466 | Cul2 |
| ENSMUSG00000017386 | -1.07375 | 0.004485 | 0.037466 | Traf4 |
| ENSMUSG00000020850 | -0.70752 | 0.004485 | 0.037466 | Prpf8 |
| ENSMUSG00000026065 | 2.370614 | 0.004487 | 0.037466 | Slc9a4 |
| ENSMUSG00000076538 | -6.50399 | 0.004489 | 0.037473 | Igkv13-84 |
| ENSMUSG00000020396 | -5.44857 | 0.004492 | 0.037473 | Nefh |
| ENSMUSG00000090785 | -5.56335 | 0.004493 | 0.037473 | Gm17116 |
| ENSMUSG00000028961 | -0.90041 | 0.004515 | 0.037635 | Pgd |
| ENSMUSG00000028333 | 0.534484 | 0.004524 | 0.0377 | Anp32b |
| ENSMUSG00000031994 | 2.92918 | 0.004529 | 0.037723 | Adamts8 |
| ENSMUSG00000038216 | 5.268635 | 0.004537 | 0.037769 | Pnmt |
| ENSMUSG00000060860 | 0.545286 | 0.00454 | 0.037784 | Ube2s |
| ENSMUSG00000001473 | 1.014163 | 0.004545 | 0.037806 | Tubb6 |
| ENSMUSG00000111059 | -2.66589 | 0.004556 | 0.037877 | Gm33838 |
| ENSMUSG00000112346 | -1.1826 | 0.004568 | 0.037967 | Gm48768 |
| ENSMUSG00000034591 | 0.7286 | 0.004579 | 0.03804 | Slc41a2 |
| ENSMUSG00000022012 | 1.113614 | 0.004587 | 0.038086 | Enox1 |
| ENSMUSG00000094800 | -2.68027 | 0.004591 | 0.038103 | Gm9780 |
| ENSMUSG00000048387 | -1.32594 | 0.004613 | 0.038275 | Osr1 |
| ENSMUSG00000030753 | 0.767514 | 0.004621 | 0.038323 | Thap12 |
| ENSMUSG00000062328 | 1.183012 | 0.004624 | 0.038333 | Rpl17 |
| ENSMUSG00000094991 | -6.45551 | 0.004635 | 0.0384 | Gm21718 |
| ENSMUSG00000016200 | -4.12756 | 0.004642 | 0.038444 | Syt14 |
| ENSMUSG00000021268 | 1.334088 | 0.004645 | 0.038455 | Meg3 |
| ENSMUSG00000092397 | 5.325064 | 0.004653 | 0.038504 | C130080G10Rik |
| ENSMUSG00000059810 | -0.73215 | 0.004677 | 0.038687 | Rgs3 |
| ENSMUSG00000000058 | -0.74251 | 0.004679 | 0.038688 | Cav2 |
| ENSMUSG00000025425 | 2.827827 | 0.004702 | 0.038862 | St8sia5 |
| ENSMUSG00000033170 | -1.43526 | 0.004709 | 0.038893 | Card10 |
| ENSMUSG00000024325 | -0.77284 | 0.00471 | 0.038893 | Ring1 |
| ENSMUSG00000040940 | -0.85393 | 0.004745 | 0.039161 | Arhgef1 |
| ENSMUSG00000025370 | -2.10946 | 0.004749 | 0.039182 | Cdh9 |
| ENSMUSG00000023010 | -0.52988 | 0.004754 | 0.039206 | Tmbim6 |
| ENSMUSG00000025877 | 1.236432 | 0.004757 | 0.03921 | Hk3 |
| ENSMUSG00000032485 | -0.59402 | 0.004799 | 0.039538 | Scap |
| ENSMUSG00000085614 | 1.406099 | 0.004801 | 0.039538 | 1700123M08Rik |
| ENSMUSG00000022187 | -5.77517 | 0.004806 | 0.039544 | Gm5546 |
| ENSMUSG00000038777 | 1.365383 | 0.004807 | 0.039544 | Sema6c |
| ENSMUSG00000066175 | -1.36382 | 0.004808 | 0.039544 | 2510046G10Rik |
| ENSMUSG00000029211 | -3.36064 | 0.004859 | 0.039952 | Gabra4 |
| ENSMUSG00000033416 | -0.79139 | 0.004865 | 0.039983 | Gucd1 |
| ENSMUSG00000039813 | -1.01951 | 0.004877 | 0.04005 | Tbc1d2 |
| ENSMUSG00000044788 | -1.41198 | 0.004877 | 0.04005 | Fads6 |
| ENSMUSG00000024500 | -1.47852 | 0.004888 | 0.040117 | Ppp2r2b |
| ENSMUSG00000026175 | -2.81287 | 0.004899 | 0.040195 | Vil1 |
| ENSMUSG00000020183 | -1.99217 | 0.004904 | 0.040205 | Cpm |
| ENSMUSG00000030092 | -3.5045 | 0.004905 | 0.040205 | Cntn6 |
| ENSMUSG00000062329 | -2.5912 | 0.004916 | 0.04028 | Cytl1 |
| ENSMUSG00000026676 | -1.7069 | 0.004924 | 0.040332 | Ccdc3 |
| ENSMUSG00000039457 | -1.1597 | 0.004929 | 0.040355 | Ppl |
| ENSMUSG00000000049 | 3.491266 | 0.004939 | 0.040414 | Apoh |
| ENSMUSG00000114693 | -5.76524 | 0.004964 | 0.040605 | Gm41177 |
| ENSMUSG00000028476 | -1.43469 | 0.004974 | 0.040672 | Reck |
| ENSMUSG00000025348 | -0.96843 | 0.004986 | 0.040747 | Itga7 |
| ENSMUSG00000020732 | -2.53226 | 0.004996 | 0.040811 | Rab37 |
| ENSMUSG00000054556 | -1.76812 | 0.005002 | 0.040811 | Gm4876 |
| ENSMUSG00000024846 | 1.59399 | 0.005003 | 0.040811 | Cst6 |
| ENSMUSG00000031262 | 1.556945 | 0.005004 | 0.040811 | Cenpi |
| ENSMUSG00000030287 | -0.83742 | 0.005004 | 0.040811 | Itpr2 |
| ENSMUSG00000112792 | 2.747273 | 0.005015 | 0.040884 | Gm38407 |
| ENSMUSG00000000340 | -0.82195 | 0.005019 | 0.040901 | Dbt |
| ENSMUSG00000006517 | -0.62838 | 0.005026 | 0.040938 | Mvd |
| ENSMUSG00000071646 | -0.51657 | 0.005042 | 0.041052 | Mta2 |
| ENSMUSG00000038060 | -2.00408 | 0.005049 | 0.041088 | Dlec1 |
| ENSMUSG00000017692 | 1.292871 | 0.005054 | 0.04111 | Rhbdl3 |
| ENSMUSG00000028064 | -0.62364 | 0.005065 | 0.041167 | Sema4a |
| ENSMUSG00000028600 | -1.1035 | 0.005065 | 0.041167 | Podn |
| ENSMUSG00000025270 | -4.5987 | 0.005088 | 0.041322 | Alas2 |
| ENSMUSG00000079494 | 2.542212 | 0.005088 | 0.041322 | Nat8f5 |
| ENSMUSG00000021565 | -3.27958 | 0.005096 | 0.041366 | Slc6a19 |
| ENSMUSG00000030154 | 1.846896 | 0.00511 | 0.041459 | Klrb1f |
| ENSMUSG00000033788 | 1.136091 | 0.005125 | 0.041564 | Dysf |
| ENSMUSG00000032596 | -0.76938 | 0.005133 | 0.041616 | Uba7 |
| ENSMUSG00000025340 | 1.101272 | 0.005158 | 0.041802 | Rabgef1 |
| ENSMUSG00000061080 | -1.66869 | 0.005177 | 0.041923 | Lsamp |
| ENSMUSG00000090124 | 1.010249 | 0.005178 | 0.041923 | Ugt1a7c |
| ENSMUSG00000021730 | -3.53404 | 0.005181 | 0.041934 | Hcn1 |
| ENSMUSG00000031098 | 4.644985 | 0.005184 | 0.041936 | Syt8 |
| ENSMUSG00000016520 | -0.69125 | 0.005189 | 0.041959 | Lnx2 |
| ENSMUSG00000067158 | -1.92863 | 0.005205 | 0.042074 | Col4a4 |
| ENSMUSG00000021208 | -2.9302 | 0.005216 | 0.042133 | Ifi27l2b |
| ENSMUSG00000027472 | 0.653668 | 0.005217 | 0.042133 | Pdrg1 |
| ENSMUSG00000030844 | 1.097529 | 0.005223 | 0.042162 | Rgs10 |
| ENSMUSG00000061099 | -1.93293 | 0.005226 | 0.042175 | Gapdhs |
| ENSMUSG00000089812 | 1.914943 | 0.005238 | 0.042253 | Gm15867 |
| ENSMUSG00000109392 | 3.459193 | 0.005245 | 0.042282 | Gm5737 |
| ENSMUSG00000062248 | 1.589106 | 0.005246 | 0.042282 | Cks2 |
| ENSMUSG00000030727 | -0.75032 | 0.005269 | 0.042443 | Rabep2 |
| ENSMUSG00000083567 | 1.201586 | 0.005274 | 0.042471 | Gm11451 |
| ENSMUSG00000018168 | -2.72816 | 0.005296 | 0.042625 | Ikzf3 |
| ENSMUSG00000027227 | 0.654319 | 0.005305 | 0.042684 | Sord |
| ENSMUSG00000030043 | 3.009458 | 0.005321 | 0.04278 | Tacr1 |
| ENSMUSG00000033880 | -0.88561 | 0.005321 | 0.04278 | Lgals3bp |
| ENSMUSG00000034040 | 1.114559 | 0.005334 | 0.04286 | Galnt17 |
| ENSMUSG00000031821 | 1.217302 | 0.005345 | 0.042931 | Gins2 |
| ENSMUSG00000028949 | 1.52654 | 0.005357 | 0.042989 | Smarcd3 |
| ENSMUSG00000017057 | 0.896445 | 0.005357 | 0.042989 | Il13ra1 |
| ENSMUSG00000028053 | -0.55073 | 0.005359 | 0.042989 | Ash1l |
| ENSMUSG00000002052 | -0.57188 | 0.005364 | 0.043003 | Supt6 |
| ENSMUSG00000021614 | 0.989704 | 0.005365 | 0.043003 | Vcan |
| ENSMUSG00000017760 | 0.540279 | 0.005375 | 0.043067 | Ctsa |
| ENSMUSG00000078300 | 1.73775 | 0.005385 | 0.043108 | Gm2606 |
| ENSMUSG00000006456 | -0.7146 | 0.005386 | 0.043108 | Rbm14 |
| ENSMUSG00000069919 | -3.86307 | 0.005387 | 0.043108 | Hba-a1 |
| ENSMUSG00000023460 | 1.138811 | 0.005397 | 0.043171 | Rab12 |
| ENSMUSG00000022022 | 1.020102 | 0.005415 | 0.043296 | Mtrf1 |
| ENSMUSG00000032679 | -0.82757 | 0.005421 | 0.043328 | Cd59a |
| ENSMUSG00000029189 | 1.521072 | 0.005424 | 0.043335 | Sel1l3 |
| ENSMUSG00000027931 | -1.60323 | 0.005432 | 0.043377 | Npr1 |
| ENSMUSG00000115099 | -2.0118 | 0.005436 | 0.043393 | AC114585.1 |
| ENSMUSG00000074217 | 2.559866 | 0.005447 | 0.043458 | 2210011C24Rik |
| ENSMUSG00000028945 | 0.503544 | 0.005504 | 0.0439 | Rheb |
| ENSMUSG00000063142 | 1.160144 | 0.005507 | 0.043901 | Kcnma1 |
| ENSMUSG00000018401 | -0.73067 | 0.005512 | 0.043921 | Mtmr4 |
| ENSMUSG00000001158 | 0.59851 | 0.005524 | 0.044005 | Snrnp27 |
| ENSMUSG00000031298 | -1.02948 | 0.005535 | 0.044069 | Adgrg2 |
| ENSMUSG00000039693 | 0.793284 | 0.005539 | 0.044083 | Msantd3 |
| ENSMUSG00000021754 | -0.743 | 0.005542 | 0.044092 | Map3k1 |
| ENSMUSG00000050812 | 0.817237 | 0.00556 | 0.044213 | Ecpas |
| ENSMUSG00000038256 | -0.80518 | 0.005567 | 0.044253 | Bcl9 |
| ENSMUSG00000105176 | 1.838409 | 0.00557 | 0.044256 | Gm43668 |
| ENSMUSG00000032547 | 0.667778 | 0.005584 | 0.044335 | Ryk |
| ENSMUSG00000039427 | -0.73719 | 0.005585 | 0.044335 | Alg1 |
| ENSMUSG00000052459 | 0.669243 | 0.005587 | 0.044335 | Atp6v1a |
| ENSMUSG00000034640 | -0.61457 | 0.005591 | 0.044352 | Tiparp |
| ENSMUSG00000055691 | -5.51477 | 0.005605 | 0.044447 | Gja6 |
| ENSMUSG00000022099 | -1.16299 | 0.005611 | 0.044474 | Dmtn |
| ENSMUSG00000102496 | -2.6057 | 0.00562 | 0.04451 | Gm36989 |
| ENSMUSG00000041426 | -1.22865 | 0.00562 | 0.04451 | Hibch |
| ENSMUSG00000032030 | 0.730486 | 0.005626 | 0.044538 | Cul5 |
| ENSMUSG00000030978 | 0.741352 | 0.005639 | 0.044618 | Rrm1 |
| ENSMUSG00000039037 | -1.40047 | 0.005642 | 0.044626 | St6galnac5 |
| ENSMUSG00000060639 | 1.630671 | 0.005649 | 0.044663 | Hist1h4i |
| ENSMUSG00000095753 | -6.09274 | 0.005662 | 0.04474 | Igkv4-53 |
| ENSMUSG00000114019 | 2.149517 | 0.005663 | 0.04474 | Gm47155 |
| ENSMUSG00000040430 | 0.939459 | 0.005672 | 0.044786 | Pitpnc1 |
| ENSMUSG00000044982 | -0.64617 | 0.005678 | 0.044816 | Sft2d3 |
| ENSMUSG00000038765 | -2.75265 | 0.005688 | 0.04488 | Lmx1b |
| ENSMUSG00000028016 | 0.679861 | 0.00569 | 0.04488 | Ints12 |
| ENSMUSG00000102098 | 4.703843 | 0.005708 | 0.044999 | 2310016D03Rik |
| ENSMUSG00000040363 | -0.69405 | 0.005715 | 0.045039 | Bcor |
| ENSMUSG00000100199 | -4.14134 | 0.00572 | 0.045054 | Gm20324 |
| ENSMUSG00000021750 | -2.33586 | 0.005725 | 0.045076 | Fam107a |
| ENSMUSG00000116895 | -1.2243 | 0.005727 | 0.045077 | Gm3435 |
| ENSMUSG00000056091 | 1.311821 | 0.005738 | 0.04514 | St3gal5 |
| ENSMUSG00000032719 | -2.46532 | 0.005742 | 0.045156 | Sbspon |
| ENSMUSG00000040883 | -0.74234 | 0.005754 | 0.045234 | Tmem205 |
| ENSMUSG00000032295 | -0.70813 | 0.005761 | 0.04527 | Man2c1 |
| ENSMUSG00000031258 | -2.47153 | 0.005787 | 0.045451 | Xkrx |
| ENSMUSG00000020695 | 1.006347 | 0.0058 | 0.045535 | Mrc2 |
| ENSMUSG00000029570 | -0.97917 | 0.005803 | 0.045542 | Lfng |
| ENSMUSG00000025779 | 0.944169 | 0.005809 | 0.045569 | Ly96 |
| ENSMUSG00000041431 | 1.943392 | 0.005815 | 0.0456 | Ccnb1 |
| ENSMUSG00000006526 | -1.13303 | 0.005834 | 0.045731 | Tmem110 |
| ENSMUSG00000033740 | 3.93718 | 0.005857 | 0.045888 | St18 |
| ENSMUSG00000025889 | -3.70042 | 0.005865 | 0.045932 | Snca |
| ENSMUSG00000026928 | 0.956427 | 0.005878 | 0.046003 | Card9 |
| ENSMUSG00000037868 | 1.275564 | 0.005879 | 0.046003 | Egr2 |
| ENSMUSG00000028626 | -5.14249 | 0.005886 | 0.046026 | Col9a2 |
| ENSMUSG00000085457 | 0.908432 | 0.005886 | 0.046026 | 1110046J04Rik |
| ENSMUSG00000036810 | 0.594868 | 0.005897 | 0.046087 | Cnep1r1 |
| ENSMUSG00000076605 | -6.19314 | 0.005899 | 0.046087 | Igkj2 |
| ENSMUSG00000026003 | -1.38446 | 0.005945 | 0.046404 | Acadl |
| ENSMUSG00000025465 | -1.12797 | 0.005946 | 0.046404 | Echs1 |
| ENSMUSG00000046324 | -0.71887 | 0.005947 | 0.046404 | Ermp1 |
| ENSMUSG00000102224 | 4.837889 | 0.00595 | 0.046412 | 4930447F24Rik |
| ENSMUSG00000023277 | 0.888958 | 0.005959 | 0.046465 | Twf2 |
| ENSMUSG00000034551 | 1.642711 | 0.005967 | 0.046506 | Hdx |
| ENSMUSG00000032181 | -1.24257 | 0.00599 | 0.046649 | Scg3 |
| ENSMUSG00000048701 | 0.673732 | 0.00599 | 0.046649 | Ccdc6 |
| ENSMUSG00000019942 | 1.985815 | 0.005993 | 0.046649 | Cdk1 |
| ENSMUSG00000072407 | 1.494237 | 0.006002 | 0.046706 | Gm6419 |
| ENSMUSG00000020946 | 0.468479 | 0.006017 | 0.046797 | Gosr2 |
| ENSMUSG00000080824 | 1.053755 | 0.00602 | 0.046803 | Gm9001 |
| ENSMUSG00000027746 | 0.572412 | 0.006023 | 0.046804 | Ufm1 |
| ENSMUSG00000044320 | 2.924287 | 0.006025 | 0.046804 | 1700001O22Rik |
| ENSMUSG00000019066 | -0.70473 | 0.006044 | 0.046937 | Rab3d |
| ENSMUSG00000038843 | -0.87232 | 0.006052 | 0.046974 | Gcnt1 |
| ENSMUSG00000092203 | 0.83351 | 0.006055 | 0.046985 | 1110038B12Rik |
| ENSMUSG00000041959 | 0.680805 | 0.006076 | 0.047122 | S100a10 |
| ENSMUSG00000087579 | 2.104619 | 0.006088 | 0.047196 | Hectd2os |
| ENSMUSG00000087095 | 1.894472 | 0.006112 | 0.047366 | Emx2os |
| ENSMUSG00000040899 | -3.9732 | 0.006157 | 0.047694 | Ccr6 |
| ENSMUSG00000041229 | -0.72051 | 0.006189 | 0.047909 | Phf8 |
| ENSMUSG00000017453 | -2.2216 | 0.00619 | 0.047909 | Pipox |
| ENSMUSG00000067825 | -0.87913 | 0.006194 | 0.047919 | Pex26 |
| ENSMUSG00000061111 | 0.727316 | 0.006198 | 0.047919 | Mcrip1 |
| ENSMUSG00000020150 | 0.736908 | 0.006198 | 0.047919 | Gamt |
| ENSMUSG00000022386 | -0.65546 | 0.006209 | 0.047976 | Trmu |
| ENSMUSG00000052305 | -3.71701 | 0.006211 | 0.047976 | Hbb-bs |
| ENSMUSG00000011179 | 0.956433 | 0.006226 | 0.048074 | Odc1 |
| ENSMUSG00000110665 | -4.75973 | 0.006233 | 0.048111 | Gm31786 |
| ENSMUSG00000031214 | -0.73044 | 0.006241 | 0.048154 | Ophn1 |
| ENSMUSG00000085412 | 2.006622 | 0.00626 | 0.048276 | Halr1 |
| ENSMUSG00000045482 | -0.48165 | 0.006281 | 0.048421 | Trrap |
| ENSMUSG00000048215 | -3.84414 | 0.006294 | 0.0485 | A630023P12Rik |
| ENSMUSG00000105881 | -1.56907 | 0.006313 | 0.048631 | 4932422M17Rik |
| ENSMUSG00000044340 | -0.79067 | 0.00632 | 0.048665 | Phlpp1 |
| ENSMUSG00000006699 | 0.487774 | 0.006325 | 0.048682 | Cdc42 |
| ENSMUSG00000043168 | -6.04889 | 0.00633 | 0.048698 | 4930426D05Rik |
| ENSMUSG00000098715 | -5.55063 | 0.006342 | 0.048772 | Gm28053 |
| ENSMUSG00000049090 | -0.61365 | 0.006346 | 0.048782 | Zadh2 |
| ENSMUSG00000023341 | 1.847154 | 0.006351 | 0.048787 | Mx2 |
| ENSMUSG00000037124 | -5.36684 | 0.006351 | 0.048787 | Trim58 |
| ENSMUSG00000020692 | -0.89622 | 0.006362 | 0.048853 | Nle1 |
| ENSMUSG00000031494 | -1.43165 | 0.006381 | 0.048976 | Cd209a |
| ENSMUSG00000030114 | 1.668227 | 0.006389 | 0.049016 | Klrg1 |
| ENSMUSG00000030208 | 1.143766 | 0.006399 | 0.049076 | Emp1 |
| ENSMUSG00000054200 | -1.41422 | 0.006403 | 0.049088 | Ffar4 |
| ENSMUSG00000029462 | 0.504872 | 0.006407 | 0.049099 | Vps29 |
| ENSMUSG00000021728 | 1.076929 | 0.006417 | 0.04915 | Emb |
| ENSMUSG00000021928 | 0.688955 | 0.006422 | 0.049175 | Ebpl |
| ENSMUSG00000071347 | -1.15472 | 0.006427 | 0.049188 | C1qtnf9 |
| ENSMUSG00000095981 | -5.19383 | 0.006454 | 0.049378 | Ighv10-1 |
| ENSMUSG00000014786 | 1.655183 | 0.006457 | 0.049378 | Slc9a5 |
| ENSMUSG00000102428 | -1.77534 | 0.006495 | 0.049654 | Pcdhga12 |
| ENSMUSG00000117029 | 4.631422 | 0.006502 | 0.049668 | CT030155.1 |
| ENSMUSG00000021103 | 0.549426 | 0.006502 | 0.049668 | Mnat1 |
| ENSMUSG00000090192 | 4.501622 | 0.006506 | 0.049678 | Gm16556 |
| ENSMUSG00000015944 | 0.871318 | 0.006518 | 0.049748 | Castor2 |
| ENSMUSG00000058809 | 1.39094 | 0.006521 | 0.049751 | Hspd1-ps3 |
| ENSMUSG00000104283 | -5.87669 | 0.006534 | 0.049833 | Gm37459 |
| ENSMUSG00000048450 | -1.03677 | 0.006543 | 0.049878 | Msx1 |

**Supplementary table 2** All GO terms of DEmRNAs

| Category | Description | pvalue | padj | Count |
| --- | --- | --- | --- | --- |
| BP | monocarboxylic acid metabolic process | 2.42E-27 | 1.36E-23 | 151 |
| BP | striated muscle cell development | 1.62E-22 | 4.54E-19 | 72 |
| BP | muscle cell development | 5.99E-21 | 1.03E-17 | 74 |
| BP | fatty acid metabolic process | 7.33E-21 | 1.03E-17 | 106 |
| BP | monocarboxylic acid biosynthetic process | 2.26E-19 | 2.53E-16 | 80 |
| BP | striated muscle cell differentiation | 8.16E-18 | 7.61E-15 | 91 |
| BP | organic acid biosynthetic process | 1.40E-17 | 1.12E-14 | 98 |
| BP | carboxylic acid biosynthetic process | 3.49E-17 | 2.44E-14 | 97 |
| BP | muscle system process | 6.90E-17 | 4.29E-14 | 105 |
| BP | muscle tissue development | 2.62E-16 | 1.47E-13 | 114 |
| BP | myofibril assembly | 4.65E-16 | 2.37E-13 | 36 |
| BP | muscle cell differentiation | 1.06E-15 | 4.94E-13 | 103 |
| BP | cofactor metabolic process | 1.70E-15 | 7.32E-13 | 117 |
| BP | striated muscle tissue development | 1.92E-15 | 7.66E-13 | 108 |
| BP | muscle organ development | 5.12E-15 | 1.91E-12 | 103 |
| BP | fatty acid biosynthetic process | 6.79E-14 | 2.37E-11 | 47 |
| BP | coenzyme metabolic process | 5.24E-13 | 1.63E-10 | 83 |
| BP | cellular component assembly involved in morphogenesis | 5.24E-13 | 1.63E-10 | 43 |
| BP | lipid biosynthetic process | 5.58E-13 | 1.64E-10 | 120 |
| BP | carbohydrate catabolic process | 1.07E-12 | 2.99E-10 | 44 |
| BP | regulation of system process | 1.30E-12 | 3.47E-10 | 114 |
| BP | skeletal muscle tissue development | 1.83E-12 | 4.66E-10 | 57 |
| BP | muscle contraction | 3.77E-12 | 9.17E-10 | 77 |
| BP | skeletal muscle organ development | 4.46E-12 | 1.04E-09 | 58 |
| BP | sarcomere organization | 8.31E-12 | 1.86E-09 | 25 |
| BP | striated muscle contraction | 7.15E-11 | 1.54E-08 | 50 |
| BP | cardiac muscle cell development | 1.03E-10 | 2.13E-08 | 33 |
| BP | cardiac cell development | 1.02E-09 | 2.03E-07 | 33 |
| BP | neutral lipid metabolic process | 3.79E-09 | 7.31E-07 | 38 |
| BP | myotube differentiation | 5.04E-09 | 9.40E-07 | 38 |
| BP | muscle fiber development | 5.89E-09 | 1.06E-06 | 27 |
| BP | acylglycerol metabolic process | 7.88E-09 | 1.38E-06 | 37 |
| BP | regulation of muscle system process | 9.89E-09 | 1.68E-06 | 58 |
| BP | pyridine nucleotide metabolic process | 1.17E-08 | 1.93E-06 | 42 |
| BP | ADP metabolic process | 1.64E-08 | 2.63E-06 | 29 |
| BP | purine nucleoside diphosphate metabolic process | 2.09E-08 | 3.17E-06 | 30 |
| BP | purine ribonucleoside diphosphate metabolic process | 2.09E-08 | 3.17E-06 | 30 |
| BP | triglyceride metabolic process | 2.25E-08 | 3.26E-06 | 32 |
| BP | small molecule catabolic process | 2.29E-08 | 3.26E-06 | 70 |
| BP | nicotinamide nucleotide metabolic process | 2.33E-08 | 3.26E-06 | 41 |
| BP | glycogen metabolic process | 2.53E-08 | 3.30E-06 | 28 |
| BP | cellular glucan metabolic process | 2.53E-08 | 3.30E-06 | 28 |
| BP | glucan metabolic process | 2.53E-08 | 3.30E-06 | 28 |
| BP | actomyosin structure organization | 3.99E-08 | 5.07E-06 | 52 |
| BP | regulation of lipid metabolic process | 4.42E-08 | 5.49E-06 | 71 |
| BP | pyridine-containing compound metabolic process | 4.81E-08 | 5.79E-06 | 42 |
| BP | oxidoreduction coenzyme metabolic process | 4.86E-08 | 5.79E-06 | 44 |
| BP | sulfur compound metabolic process | 6.02E-08 | 6.98E-06 | 62 |
| BP | organic acid catabolic process | 6.27E-08 | 6.98E-06 | 54 |
| BP | carboxylic acid catabolic process | 6.27E-08 | 6.98E-06 | 54 |
| BP | skeletal muscle contraction | 6.36E-08 | 6.98E-06 | 18 |
| BP | ribonucleoside diphosphate metabolic process | 7.44E-08 | 8.00E-06 | 30 |
| BP | skeletal muscle cell differentiation | 7.80E-08 | 8.23E-06 | 27 |
| BP | glycolytic process | 8.56E-08 | 8.74E-06 | 26 |
| BP | blood circulation | 8.60E-08 | 8.74E-06 | 95 |
| BP | generation of precursor metabolites and energy | 9.52E-08 | 9.51E-06 | 76 |
| BP | monocarboxylic acid catabolic process | 9.71E-08 | 9.53E-06 | 35 |
| BP | cellular carbohydrate catabolic process | 1.08E-07 | 1.04E-05 | 16 |
| BP | pentose-phosphate shunt | 1.12E-07 | 1.06E-05 | 11 |
| BP | energy reserve metabolic process | 1.16E-07 | 1.08E-05 | 29 |
| BP | ATP generation from ADP | 1.21E-07 | 1.11E-05 | 26 |
| BP | circulatory system process | 1.35E-07 | 1.22E-05 | 95 |
| BP | lipid catabolic process | 1.37E-07 | 1.22E-05 | 65 |
| BP | glucose 6-phosphate metabolic process | 1.50E-07 | 1.31E-05 | 12 |
| BP | regulation of ion transmembrane transport | 1.69E-07 | 1.46E-05 | 90 |
| BP | pyruvate biosynthetic process | 2.35E-07 | 1.99E-05 | 26 |
| BP | steroid metabolic process | 2.47E-07 | 2.06E-05 | 60 |
| BP | pyruvate metabolic process | 2.64E-07 | 2.14E-05 | 31 |
| BP | fatty acid catabolic process | 2.64E-07 | 2.14E-05 | 31 |
| BP | pyridine nucleotide biosynthetic process | 2.82E-07 | 2.23E-05 | 29 |
| BP | positive regulation of lipid biosynthetic process | 2.83E-07 | 2.23E-05 | 27 |
| BP | nucleoside diphosphate metabolic process | 2.86E-07 | 2.23E-05 | 32 |
| BP | regulation of skeletal muscle tissue development | 3.32E-07 | 2.54E-05 | 22 |
| BP | cellular carbohydrate metabolic process | 4.09E-07 | 3.06E-05 | 61 |
| BP | regulation of muscle organ development | 4.11E-07 | 3.06E-05 | 41 |
| BP | nucleoside diphosphate phosphorylation | 4.41E-07 | 3.20E-05 | 28 |
| BP | nicotinamide nucleotide biosynthetic process | 4.41E-07 | 3.20E-05 | 28 |
| BP | cellular lipid catabolic process | 4.64E-07 | 3.33E-05 | 47 |
| BP | regulation of striated muscle tissue development | 6.30E-07 | 4.46E-05 | 40 |
| BP | cardiac muscle cell differentiation | 6.67E-07 | 4.66E-05 | 36 |
| BP | NADP metabolic process | 6.93E-07 | 4.73E-05 | 17 |
| BP | skeletal muscle fiber development | 6.93E-07 | 4.73E-05 | 17 |
| BP | myotube cell development | 7.54E-07 | 5.08E-05 | 18 |
| BP | nucleotide phosphorylation | 7.72E-07 | 5.12E-05 | 28 |
| BP | brown fat cell differentiation | 7.78E-07 | 5.12E-05 | 19 |
| BP | fat cell differentiation | 8.11E-07 | 5.27E-05 | 55 |
| BP | pyridine-containing compound biosynthetic process | 8.45E-07 | 5.43E-05 | 29 |
| BP | ribose phosphate metabolic process | 8.80E-07 | 5.60E-05 | 89 |
| BP | myeloid cell apoptotic process | 9.87E-07 | 6.21E-05 | 16 |
| BP | positive regulation of ion transport | 1.03E-06 | 6.42E-05 | 63 |
| BP | regulation of muscle tissue development | 1.14E-06 | 6.99E-05 | 40 |
| BP | cofactor biosynthetic process | 1.22E-06 | 7.41E-05 | 52 |
| BP | nucleotide catabolic process | 1.28E-06 | 7.59E-05 | 35 |
| BP | regulation of metal ion transport | 1.29E-06 | 7.59E-05 | 80 |
| BP | cardiac muscle fiber development | 1.29E-06 | 7.59E-05 | 9 |
| BP | positive regulation of triglyceride metabolic process | 1.37E-06 | 7.96E-05 | 13 |
| BP | monosaccharide metabolic process | 1.47E-06 | 8.39E-05 | 55 |
| BP | glycerolipid metabolic process | 1.48E-06 | 8.39E-05 | 67 |
| BP | regulation of cation transmembrane transport | 1.48E-06 | 8.39E-05 | 67 |
| BP | nucleoside phosphate catabolic process | 1.53E-06 | 8.54E-05 | 37 |
| BP | regulation of lipid biosynthetic process | 1.74E-06 | 9.61E-05 | 39 |
| BP | glyceraldehyde-3-phosphate metabolic process | 1.81E-06 | 9.94E-05 | 11 |
| BP | lipid oxidation | 1.90E-06 | 0.000103 | 30 |
| BP | cardiac muscle tissue development | 1.96E-06 | 0.000105 | 55 |
| BP | coenzyme biosynthetic process | 2.02E-06 | 0.000108 | 43 |
| BP | regulation of small molecule metabolic process | 2.24E-06 | 0.000118 | 79 |
| BP | sarcoplasmic reticulum calcium ion transport | 2.51E-06 | 0.000131 | 13 |
| BP | ribose phosphate biosynthetic process | 2.82E-06 | 0.000146 | 57 |
| BP | nucleobase-containing small molecule biosynthetic process | 3.05E-06 | 0.000156 | 42 |
| BP | acyl-CoA metabolic process | 3.53E-06 | 0.000178 | 24 |
| BP | thioester metabolic process | 3.53E-06 | 0.000178 | 24 |
| BP | fatty acid oxidation | 3.75E-06 | 0.000187 | 29 |
| BP | regulation of myeloid cell apoptotic process | 5.36E-06 | 0.000265 | 14 |
| BP | response to peptide | 5.57E-06 | 0.000273 | 71 |
| BP | regulation of systemic arterial blood pressure mediated by a chemical signal | 5.61E-06 | 0.000273 | 18 |
| BP | cellular polysaccharide metabolic process | 5.84E-06 | 0.000282 | 28 |
| BP | hexose metabolic process | 6.10E-06 | 0.000292 | 49 |
| BP | multicellular organismal movement | 7.25E-06 | 0.000341 | 19 |
| BP | musculoskeletal movement | 7.25E-06 | 0.000341 | 19 |
| BP | positive regulation of lipid metabolic process | 7.39E-06 | 0.000345 | 36 |
| BP | response to stimulus involved in regulation of muscle adaptation | 7.68E-06 | 0.000355 | 8 |
| BP | response to corticosteroid | 7.98E-06 | 0.000363 | 24 |
| BP | monosaccharide biosynthetic process | 7.98E-06 | 0.000363 | 24 |
| BP | positive regulation of triglyceride biosynthetic process | 8.83E-06 | 0.000395 | 10 |
| BP | muscle cell cellular homeostasis | 8.83E-06 | 0.000395 | 10 |
| BP | purine ribonucleotide biosynthetic process | 1.23E-05 | 0.000544 | 53 |
| BP | response to glucocorticoid | 1.25E-05 | 0.000552 | 23 |
| BP | purine-containing compound biosynthetic process | 1.28E-05 | 0.00056 | 55 |
| BP | organophosphate biosynthetic process | 1.29E-05 | 0.00056 | 93 |
| BP | neutral lipid biosynthetic process | 1.37E-05 | 0.000587 | 15 |
| BP | acylglycerol biosynthetic process | 1.37E-05 | 0.000587 | 15 |
| BP | regulation of triglyceride biosynthetic process | 1.39E-05 | 0.00059 | 11 |
| BP | purine nucleotide biosynthetic process | 1.42E-05 | 0.000599 | 54 |
| BP | purine-containing compound metabolic process | 1.58E-05 | 0.000662 | 89 |
| BP | cellular metal ion homeostasis | 1.65E-05 | 0.000684 | 96 |
| BP | response to insulin | 1.72E-05 | 0.00071 | 47 |
| BP | response to activity | 1.78E-05 | 0.000723 | 12 |
| BP | myoblast differentiation | 1.78E-05 | 0.000723 | 26 |
| BP | ribonucleotide biosynthetic process | 1.83E-05 | 0.000736 | 54 |
| BP | sterol metabolic process | 1.89E-05 | 0.000757 | 32 |
| BP | negative regulation of protein polymerization | 1.97E-05 | 0.00078 | 22 |
| BP | cardiocyte differentiation | 1.98E-05 | 0.00078 | 39 |
| BP | regulation of skeletal muscle contraction | 2.05E-05 | 0.0008 | 8 |
| BP | triglyceride biosynthetic process | 2.08E-05 | 0.000808 | 14 |
| BP | leukocyte chemotaxis | 2.10E-05 | 0.000809 | 45 |
| BP | potassium ion transport | 2.41E-05 | 0.000921 | 49 |
| BP | organic hydroxy compound metabolic process | 2.42E-05 | 0.000921 | 80 |
| BP | response to antibiotic | 2.57E-05 | 0.00097 | 52 |
| BP | polysaccharide metabolic process | 2.60E-05 | 0.000976 | 29 |
| BP | positive regulation of small molecule metabolic process | 2.64E-05 | 0.000984 | 41 |
| BP | regulation of fatty acid metabolic process | 2.67E-05 | 0.000985 | 23 |
| BP | monovalent inorganic cation transport | 2.68E-05 | 0.000985 | 83 |
| BP | regulation of muscle adaptation | 2.78E-05 | 0.001015 | 25 |
| BP | regulation of myoblast differentiation | 2.85E-05 | 0.001035 | 20 |
| BP | positive regulation of transmembrane transport | 2.87E-05 | 0.001036 | 46 |
| BP | response to peptide hormone | 3.02E-05 | 0.001082 | 60 |
| BP | regulation of lipid localization | 3.07E-05 | 0.001095 | 33 |
| BP | lipid storage | 3.09E-05 | 0.001095 | 21 |
| BP | actin filament capping | 3.12E-05 | 0.001099 | 14 |
| BP | alcohol biosynthetic process | 3.16E-05 | 0.001105 | 29 |
| BP | cellular potassium ion transport | 3.22E-05 | 0.001111 | 42 |
| BP | potassium ion transmembrane transport | 3.22E-05 | 0.001111 | 42 |
| BP | muscle adaptation | 3.27E-05 | 0.001124 | 28 |
| BP | ribonucleotide metabolic process | 3.38E-05 | 0.001153 | 81 |
| BP | carbohydrate biosynthetic process | 3.63E-05 | 0.001224 | 45 |
| BP | heart contraction | 3.63E-05 | 0.001224 | 45 |
| BP | actin filament depolymerization | 4.00E-05 | 0.001339 | 18 |
| BP | muscle hypertrophy | 4.23E-05 | 0.001407 | 26 |
| BP | purine ribonucleotide metabolic process | 4.27E-05 | 0.001407 | 79 |
| BP | negative regulation of actin filament depolymerization | 4.28E-05 | 0.001407 | 15 |
| BP | neutrophil chemotaxis | 4.30E-05 | 0.001407 | 25 |
| BP | secondary alcohol metabolic process | 4.39E-05 | 0.001428 | 30 |
| BP | striated muscle myosin thick filament assembly | 4.44E-05 | 0.00143 | 7 |
| BP | calcium ion transport | 4.45E-05 | 0.00143 | 74 |
| BP | cholesterol metabolic process | 4.62E-05 | 0.001478 | 29 |
| BP | regulation of actin filament depolymerization | 4.66E-05 | 0.001482 | 17 |
| BP | neutrophil migration | 4.84E-05 | 0.00153 | 28 |
| BP | organophosphate catabolic process | 4.90E-05 | 0.001542 | 42 |
| BP | cellular response to ketone | 5.38E-05 | 0.001673 | 23 |
| BP | heart process | 5.40E-05 | 0.001673 | 45 |
| BP | myeloid leukocyte migration | 5.41E-05 | 0.001673 | 41 |
| BP | regulation of blood pressure | 5.62E-05 | 0.001729 | 42 |
| BP | cholesterol biosynthetic process | 6.04E-05 | 0.001848 | 15 |
| BP | response to toxic substance | 6.12E-05 | 0.001862 | 63 |
| BP | cellular response to lipid | 6.21E-05 | 0.00187 | 86 |
| BP | glucose metabolic process | 6.22E-05 | 0.00187 | 41 |
| BP | granulocyte migration | 6.51E-05 | 0.001938 | 32 |
| BP | fatty acid beta-oxidation | 6.55E-05 | 0.001938 | 21 |
| BP | hexose biosynthetic process | 6.55E-05 | 0.001938 | 21 |
| BP | alcohol metabolic process | 6.67E-05 | 0.001963 | 53 |
| BP | purine nucleotide metabolic process | 6.97E-05 | 0.002041 | 80 |
| BP | positive regulation of cation transmembrane transport | 7.11E-05 | 0.002071 | 33 |
| BP | regulation of carbohydrate catabolic process | 7.34E-05 | 0.002128 | 16 |
| BP | regulation of transporter activity | 7.46E-05 | 0.002152 | 52 |
| BP | organic hydroxy compound biosynthetic process | 7.75E-05 | 0.002221 | 44 |
| BP | glycogen catabolic process | 7.82E-05 | 0.002221 | 9 |
| BP | glucan catabolic process | 7.82E-05 | 0.002221 | 9 |
| BP | nucleotide biosynthetic process | 7.94E-05 | 0.002244 | 59 |
| BP | striated muscle hypertrophy | 8.01E-05 | 0.002253 | 25 |
| BP | long-chain fatty acid metabolic process | 8.06E-05 | 0.002255 | 20 |
| BP | regulation of triglyceride metabolic process | 8.42E-05 | 0.002344 | 15 |
| BP | regulation of acute inflammatory response | 9.27E-05 | 0.002556 | 18 |
| BP | negative regulation of actin filament polymerization | 9.27E-05 | 0.002556 | 18 |
| BP | energy derivation by oxidation of organic compounds | 9.35E-05 | 0.002566 | 51 |
| BP | purine nucleoside monophosphate biosynthetic process | 9.76E-05 | 0.00265 | 33 |
| BP | purine ribonucleoside monophosphate biosynthetic process | 9.76E-05 | 0.00265 | 33 |
| BP | polysaccharide catabolic process | 9.88E-05 | 0.00267 | 10 |
| BP | myosin filament assembly | 0.000109 | 0.002922 | 7 |
| BP | secondary alcohol biosynthetic process | 0.000116 | 0.003098 | 15 |
| BP | divalent metal ion transport | 0.000119 | 0.00316 | 79 |
| BP | granulocyte chemotaxis | 0.000121 | 0.003205 | 28 |
| BP | regulation of heart rate | 0.000123 | 0.003244 | 24 |
| BP | cardiac muscle hypertrophy | 0.000123 | 0.003244 | 24 |
| BP | regulation of peptide secretion | 0.000126 | 0.003299 | 84 |
| BP | cellular response to hormone stimulus | 0.000131 | 0.003401 | 91 |
| BP | regulation of transmembrane transporter activity | 0.000131 | 0.003401 | 49 |
| BP | alditol metabolic process | 0.000139 | 0.003529 | 9 |
| BP | cellular polysaccharide catabolic process | 0.000139 | 0.003529 | 9 |
| BP | cardiac myofibril assembly | 0.000139 | 0.003529 | 9 |
| BP | regulation of skeletal muscle cell differentiation | 0.000139 | 0.003529 | 9 |
| BP | divalent inorganic cation transport | 0.000139 | 0.003531 | 79 |
| BP | carboxylic acid transport | 0.000142 | 0.003569 | 54 |
| BP | lipid modification | 0.000143 | 0.003576 | 43 |
| BP | nucleoside phosphate biosynthetic process | 0.000145 | 0.003623 | 59 |
| BP | organic acid transmembrane transport | 0.00015 | 0.00372 | 24 |
| BP | carboxylic acid transmembrane transport | 0.00015 | 0.00372 | 24 |
| BP | organic acid transport | 0.000157 | 0.003872 | 54 |
| BP | cardiac muscle contraction | 0.000158 | 0.003879 | 29 |
| BP | negative regulation of lipid catabolic process | 0.000161 | 0.003923 | 10 |
| BP | regulation of muscle contraction | 0.000163 | 0.00396 | 34 |
| BP | leukocyte migration | 0.000172 | 0.004175 | 60 |
| BP | renal system process | 0.000173 | 0.004175 | 25 |
| BP | sterol biosynthetic process | 0.000176 | 0.004216 | 16 |
| BP | positive regulation of blood pressure | 0.000176 | 0.004216 | 16 |
| BP | ribonucleoside monophosphate biosynthetic process | 0.000179 | 0.004258 | 33 |
| BP | regulation of blood circulation | 0.000184 | 0.004367 | 46 |
| BP | response to muscle activity | 0.000187 | 0.004367 | 8 |
| BP | sequestering of triglyceride | 0.000187 | 0.004367 | 8 |
| BP | white fat cell differentiation | 0.000187 | 0.004367 | 8 |
| BP | regulation of protein secretion | 0.000199 | 0.004635 | 78 |
| BP | alpha-amino acid metabolic process | 0.0002 | 0.004654 | 41 |
| BP | maintenance of location | 0.000202 | 0.004669 | 57 |
| BP | response to metal ion | 0.000207 | 0.004756 | 46 |
| BP | cellular calcium ion homeostasis | 0.000214 | 0.0049 | 76 |
| BP | chemokine-mediated signaling pathway | 0.000231 | 0.005185 | 16 |
| BP | physiological muscle hypertrophy | 0.000232 | 0.005185 | 12 |
| BP | physiological cardiac muscle hypertrophy | 0.000232 | 0.005185 | 12 |
| BP | acetyl-CoA metabolic process | 0.000232 | 0.005185 | 12 |
| BP | regulation of fatty acid biosynthetic process | 0.000232 | 0.005185 | 12 |
| BP | cell growth involved in cardiac muscle cell development | 0.000232 | 0.005185 | 12 |
| BP | positive regulation of transporter activity | 0.000233 | 0.005185 | 26 |
| BP | positive regulation of secretion by cell | 0.000241 | 0.005357 | 77 |
| BP | positive regulation of acute inflammatory response | 0.000248 | 0.005453 | 11 |
| BP | positive regulation of skeletal muscle tissue development | 0.000248 | 0.005453 | 11 |
| BP | positive regulation of ion transmembrane transport | 0.000249 | 0.005453 | 34 |
| BP | steroid biosynthetic process | 0.00025 | 0.005453 | 31 |
| BP | negative regulation of protein depolymerization | 0.000254 | 0.005514 | 20 |
| BP | secondary metabolic process | 0.000255 | 0.005514 | 19 |
| BP | lipid homeostasis | 0.000256 | 0.005514 | 29 |
| BP | regulation of cellular ketone metabolic process | 0.000256 | 0.005514 | 27 |
| BP | response to inorganic substance | 0.000267 | 0.005716 | 72 |
| BP | regulation of cation channel activity | 0.000286 | 0.006094 | 34 |
| BP | carbohydrate homeostasis | 0.000288 | 0.006094 | 46 |
| BP | glucose homeostasis | 0.000288 | 0.006094 | 46 |
| BP | positive regulation of fatty acid metabolic process | 0.000289 | 0.006094 | 13 |
| BP | regulation of cytosolic calcium ion concentration | 0.000293 | 0.006153 | 61 |
| BP | cellular response to peptide | 0.000303 | 0.006339 | 51 |
| BP | gluconeogenesis | 0.000319 | 0.00661 | 19 |
| BP | regulation of myotube differentiation | 0.000319 | 0.00661 | 18 |
| BP | positive regulation of cation channel activity | 0.000319 | 0.00661 | 18 |
| BP | ATP biosynthetic process | 0.000326 | 0.006714 | 28 |
| BP | cellular response to xenobiotic stimulus | 0.000326 | 0.006714 | 26 |
| BP | regulation of myoblast proliferation | 0.000332 | 0.006796 | 8 |
| BP | regulation of calcium ion transport | 0.000345 | 0.007046 | 49 |
| BP | lipid localization | 0.000348 | 0.007077 | 64 |
| BP | regulation of protein depolymerization | 0.000354 | 0.007181 | 22 |
| BP | negative regulation of protein transport | 0.00036 | 0.007269 | 39 |
| BP | nucleoside monophosphate biosynthetic process | 0.000362 | 0.007294 | 33 |
| BP | regulation of coenzyme metabolic process | 0.000366 | 0.007341 | 15 |
| BP | ribonucleoside triphosphate biosynthetic process | 0.000368 | 0.007351 | 30 |
| BP | regulation of hormone levels | 0.000369 | 0.007352 | 85 |
| BP | negative regulation of protein complex disassembly | 0.000371 | 0.007359 | 21 |
| BP | regulation of oxidative phosphorylation | 0.000378 | 0.007441 | 9 |
| BP | release of sequestered calcium ion into cytosol by sarcoplasmic reticulum | 0.000378 | 0.007441 | 9 |
| BP | regulation of nucleotide metabolic process | 0.000383 | 0.007524 | 35 |
| BP | cellular response to organonitrogen compound | 0.000395 | 0.007733 | 78 |
| BP | endocrine process | 0.000402 | 0.007845 | 23 |
| BP | positive regulation of cytosolic calcium ion concentration | 0.00041 | 0.007962 | 55 |
| BP | response to wounding | 0.000411 | 0.007962 | 77 |
| BP | negative regulation of cytoskeleton organization | 0.000415 | 0.008002 | 33 |
| BP | response to steroid hormone | 0.000423 | 0.008125 | 49 |
| BP | regulation of developmental growth | 0.000439 | 0.008379 | 67 |
| BP | ATP metabolic process | 0.000446 | 0.008379 | 45 |
| BP | fatty acid elongation | 0.000448 | 0.008379 | 7 |
| BP | myosin filament organization | 0.000448 | 0.008379 | 7 |
| BP | very long-chain fatty acid biosynthetic process | 0.000448 | 0.008379 | 7 |
| BP | regulation of cellular carbohydrate catabolic process | 0.000448 | 0.008379 | 7 |
| BP | short-chain fatty acid metabolic process | 0.000448 | 0.008379 | 7 |
| BP | regulation of skeletal muscle fiber development | 0.000448 | 0.008379 | 7 |
| BP | response to nutrient levels | 0.000482 | 0.008986 | 59 |
| BP | cellular aldehyde metabolic process | 0.000486 | 0.009041 | 19 |
| BP | regulation of ion transmembrane transporter activity | 0.00049 | 0.00907 | 46 |
| BP | cytokine secretion | 0.000506 | 0.009339 | 43 |
| BP | organic hydroxy compound transport | 0.000512 | 0.009416 | 41 |
| BP | positive regulation of secretion | 0.000523 | 0.009601 | 80 |
| BP | regulation of muscle cell differentiation | 0.00054 | 0.00986 | 33 |
| BP | purine ribonucleoside triphosphate biosynthetic process | 0.000541 | 0.00986 | 29 |
| BP | negative regulation of lipid metabolic process | 0.000543 | 0.009866 | 21 |
| BP | negative regulation of myeloid cell apoptotic process | 0.000557 | 0.01005 | 8 |
| BP | positive regulation of fatty acid biosynthetic process | 0.000557 | 0.01005 | 8 |
| BP | regulation of striated muscle cell differentiation | 0.000565 | 0.01013 | 27 |
| BP | monosaccharide catabolic process | 0.000566 | 0.01013 | 10 |
| BP | tissue remodeling | 0.000567 | 0.01013 | 36 |
| BP | regulation of amine transport | 0.000571 | 0.010178 | 23 |
| BP | negative regulation of establishment of protein localization | 0.000575 | 0.010206 | 39 |
| BP | positive regulation of potassium ion transport | 0.00058 | 0.010275 | 14 |
| BP | negative regulation of supramolecular fiber organization | 0.000584 | 0.010294 | 31 |
| BP | toxin metabolic process | 0.000587 | 0.010294 | 9 |
| BP | release of sequestered calcium ion into cytosol by endoplasmic reticulum | 0.000587 | 0.010294 | 9 |
| BP | regulation of hormone secretion | 0.000598 | 0.010459 | 51 |
| BP | purine nucleoside triphosphate biosynthetic process | 0.000624 | 0.010869 | 29 |
| BP | positive regulation of lipid localization | 0.000653 | 0.01134 | 21 |
| BP | calcium ion homeostasis | 0.000663 | 0.011484 | 76 |
| BP | regeneration | 0.000689 | 0.01186 | 28 |
| BP | positive regulation of striated muscle tissue development | 0.000691 | 0.01186 | 20 |
| BP | positive regulation of muscle organ development | 0.000691 | 0.01186 | 20 |
| BP | regulation of lipid storage | 0.000703 | 0.012007 | 13 |
| BP | response to acid chemical | 0.000704 | 0.012007 | 48 |
| BP | leukocyte homeostasis | 0.00072 | 0.012165 | 26 |
| BP | regulation of vascular endothelial growth factor production | 0.000724 | 0.012165 | 11 |
| BP | skeletal muscle tissue regeneration | 0.000724 | 0.012165 | 11 |
| BP | neutral lipid catabolic process | 0.000724 | 0.012165 | 11 |
| BP | acylglycerol catabolic process | 0.000724 | 0.012165 | 11 |
| BP | muscle cell apoptotic process | 0.000729 | 0.012206 | 22 |
| BP | cellular divalent inorganic cation homeostasis | 0.000733 | 0.012211 | 77 |
| BP | heart growth | 0.000736 | 0.012211 | 24 |
| BP | negative regulation of blood vessel diameter | 0.000736 | 0.012211 | 24 |
| BP | regulation of ATP metabolic process | 0.000755 | 0.012496 | 18 |
| BP | nucleoside triphosphate biosynthetic process | 0.000761 | 0.012566 | 31 |
| BP | regulation of muscle cell apoptotic process | 0.000781 | 0.012848 | 21 |
| BP | cellular modified amino acid metabolic process | 0.00079 | 0.012935 | 33 |
| BP | acute inflammatory response | 0.000793 | 0.012935 | 28 |
| BP | oxaloacetate metabolic process | 0.000798 | 0.012935 | 7 |
| BP | adult heart development | 0.000798 | 0.012935 | 7 |
| BP | negative regulation of skeletal muscle tissue development | 0.000798 | 0.012935 | 7 |
| BP | hormone secretion | 0.000801 | 0.012959 | 59 |
| BP | cellular ketone metabolic process | 0.000809 | 0.01304 | 36 |
| BP | regulation of systemic arterial blood pressure by renin-angiotensin | 0.000816 | 0.013081 | 10 |
| BP | positive regulation of myoblast differentiation | 0.000816 | 0.013081 | 10 |
| BP | positive regulation of muscle tissue development | 0.000832 | 0.013281 | 20 |
| BP | negative regulation of developmental growth | 0.000836 | 0.013281 | 26 |
| BP | response to ketone | 0.000836 | 0.013281 | 26 |
| BP | cellular amino acid metabolic process | 0.000865 | 0.013664 | 51 |
| BP | cell chemotaxis | 0.000865 | 0.013664 | 51 |
| BP | regulation of vasoconstriction | 0.000879 | 0.013861 | 19 |
| BP | collagen catabolic process | 0.000882 | 0.013863 | 9 |
| BP | protein dephosphorylation | 0.000891 | 0.013962 | 46 |
| BP | purine ribonucleoside monophosphate metabolic process | 0.000935 | 0.014616 | 48 |
| BP | regulation of epithelial cell migration | 0.00096 | 0.014962 | 42 |
| BP | antigen processing and presentation of peptide antigen | 0.000978 | 0.015204 | 16 |
| BP | angiogenesis | 0.00099 | 0.015347 | 84 |
| BP | very long-chain fatty acid metabolic process | 0.000995 | 0.015376 | 11 |
| BP | response to xenobiotic stimulus | 0.001017 | 0.015672 | 36 |
| BP | muscle organ morphogenesis | 0.00102 | 0.015676 | 22 |
| BP | purine nucleoside monophosphate metabolic process | 0.001026 | 0.015727 | 48 |
| BP | glycolytic process through fructose-6-phosphate | 0.001084 | 0.016564 | 6 |
| BP | amine transport | 0.0011 | 0.016732 | 23 |
| BP | digestive system process | 0.001103 | 0.016732 | 21 |
| BP | negative regulation of secretion | 0.001103 | 0.016732 | 45 |
| BP | antigen processing and presentation of peptide antigen via MHC class I | 0.001119 | 0.016837 | 12 |
| BP | positive regulation of myotube differentiation | 0.001119 | 0.016837 | 12 |
| BP | positive regulation of neutrophil migration | 0.001119 | 0.016837 | 12 |
| BP | ribonucleoside triphosphate metabolic process | 0.001125 | 0.016868 | 48 |
| BP | negative regulation of transport | 0.001154 | 0.017267 | 82 |
| BP | regulation of muscle hypertrophy | 0.001171 | 0.017475 | 17 |
| BP | cardiac muscle tissue growth | 0.0012 | 0.017852 | 22 |
| BP | action potential | 0.001224 | 0.018164 | 29 |
| BP | actin filament organization | 0.001227 | 0.018164 | 68 |
| BP | regulation of action potential | 0.001232 | 0.018192 | 15 |
| BP | inflammatory cell apoptotic process | 0.001287 | 0.018953 | 9 |
| BP | positive regulation of cell migration | 0.001298 | 0.019068 | 85 |
| BP | negative regulation of secretion by cell | 0.001321 | 0.019351 | 41 |
| BP | regulation of systemic arterial blood pressure by circulatory renin-angiotensin | 0.001332 | 0.019361 | 7 |
| BP | glycerol metabolic process | 0.001332 | 0.019361 | 7 |
| BP | response to stilbenoid | 0.001332 | 0.019361 | 7 |
| BP | peroxisome organization | 0.001344 | 0.019422 | 11 |
| BP | vascular endothelial growth factor production | 0.001344 | 0.019422 | 11 |
| BP | ribonucleoside monophosphate metabolic process | 0.001347 | 0.019422 | 48 |
| BP | negative regulation of intracellular transport | 0.001352 | 0.019451 | 18 |
| BP | nucleoside bisphosphate metabolic process | 0.001369 | 0.019544 | 8 |
| BP | ribonucleoside bisphosphate metabolic process | 0.001369 | 0.019544 | 8 |
| BP | purine nucleoside bisphosphate metabolic process | 0.001369 | 0.019544 | 8 |
| BP | purine ribonucleoside triphosphate metabolic process | 0.001386 | 0.019726 | 47 |
| BP | divalent inorganic cation homeostasis | 0.001407 | 0.019977 | 79 |
| BP | syncytium formation | 0.001426 | 0.020198 | 17 |
| BP | signal release | 0.001474 | 0.020823 | 75 |
| BP | superoxide metabolic process | 0.001488 | 0.020968 | 16 |
| BP | tissue regeneration | 0.001532 | 0.021542 | 13 |
| BP | drug transport | 0.001577 | 0.022118 | 36 |
| BP | positive regulation of leukocyte migration | 0.001596 | 0.022325 | 30 |
| BP | regulation of purine nucleotide metabolic process | 0.001611 | 0.022479 | 32 |
| BP | hormone transport | 0.001636 | 0.02267 | 59 |
| BP | antibiotic metabolic process | 0.001637 | 0.02267 | 25 |
| BP | protein depolymerization | 0.001637 | 0.02267 | 25 |
| BP | vasoconstriction | 0.001642 | 0.022684 | 22 |
| BP | nucleoside monophosphate metabolic process | 0.001698 | 0.023398 | 49 |
| BP | regulation of lipase activity | 0.001726 | 0.023693 | 17 |
| BP | regulation of cytokine secretion | 0.00173 | 0.023693 | 37 |
| BP | leukocyte mediated cytotoxicity | 0.001735 | 0.023693 | 23 |
| BP | positive regulation of MAPK cascade | 0.001736 | 0.023693 | 84 |
| BP | regulation of angiogenesis | 0.00175 | 0.023823 | 53 |
| BP | Notch signaling pathway | 0.00179 | 0.024307 | 34 |
| BP | regulation of insulin secretion | 0.001801 | 0.024403 | 33 |
| BP | positive regulation of potassium ion transmembrane transporter activity | 0.00183 | 0.02473 | 9 |
| BP | negative regulation of growth | 0.001849 | 0.024924 | 49 |
| BP | temperature homeostasis | 0.001901 | 0.025456 | 12 |
| BP | regulation of systemic arterial blood pressure by hormone | 0.001901 | 0.025456 | 12 |
| BP | response to oxygen levels | 0.001902 | 0.025456 | 42 |
| BP | positive regulation of ion transmembrane transporter activity | 0.001911 | 0.025457 | 22 |
| BP | regulation of potassium ion transport | 0.001911 | 0.025457 | 22 |
| BP | regulation of sequestering of triglyceride | 0.001935 | 0.025604 | 6 |
| BP | positive regulation of epidermal growth factor-activated receptor activity | 0.001935 | 0.025604 | 6 |
| BP | regulation of organic acid transport | 0.001942 | 0.025604 | 18 |
| BP | striated muscle adaptation | 0.001945 | 0.025604 | 13 |
| BP | regulation of amino acid transport | 0.001945 | 0.025604 | 13 |
| BP | muscle tissue morphogenesis | 0.001962 | 0.025774 | 20 |
| BP | negative regulation of protein complex assembly | 0.002017 | 0.026376 | 29 |
| BP | cytosolic calcium ion transport | 0.002024 | 0.026376 | 32 |
| BP | heat generation | 0.00203 | 0.026376 | 8 |
| BP | myoblast proliferation | 0.00203 | 0.026376 | 8 |
| BP | cellular response to peptide hormone stimulus | 0.002032 | 0.026376 | 43 |
| BP | skeletal system development | 0.002085 | 0.026952 | 80 |
| BP | regulation of systemic arterial blood pressure | 0.002086 | 0.026952 | 24 |
| BP | estrogen metabolic process | 0.002111 | 0.027022 | 7 |
| BP | lipid particle organization | 0.002111 | 0.027022 | 7 |
| BP | neurotransmitter catabolic process | 0.002111 | 0.027022 | 7 |
| BP | hormone catabolic process | 0.002111 | 0.027022 | 7 |
| BP | regulation of cardiac muscle hypertrophy | 0.002204 | 0.028088 | 16 |
| BP | regulation of cofactor metabolic process | 0.002204 | 0.028088 | 16 |
| BP | response to extracellular stimulus | 0.002276 | 0.028943 | 60 |
| BP | monoamine transport | 0.002301 | 0.029037 | 20 |
| BP | dicarboxylic acid transport | 0.00231 | 0.029037 | 18 |
| BP | myeloid cell development | 0.00231 | 0.029037 | 18 |
| BP | regulation of potassium ion transmembrane transport | 0.00231 | 0.029037 | 18 |
| BP | regulation of lipid transport | 0.002313 | 0.029037 | 23 |
| BP | glutathione metabolic process | 0.002315 | 0.029037 | 15 |
| BP | positive regulation of peptide secretion | 0.002403 | 0.030082 | 53 |
| BP | regulation of cysteine-type endopeptidase activity | 0.002434 | 0.030392 | 43 |
| BP | positive regulation of leukocyte chemotaxis | 0.002443 | 0.030397 | 21 |
| BP | glycerolipid catabolic process | 0.002445 | 0.030397 | 13 |
| BP | regulation of endothelial cell proliferation | 0.002499 | 0.031004 | 26 |
| BP | insulin secretion | 0.002531 | 0.031284 | 38 |
| BP | negative regulation of muscle hypertrophy | 0.002541 | 0.031284 | 9 |
| BP | eosinophil migration | 0.002541 | 0.031284 | 9 |
| BP | regulation of endothelial cell migration | 0.002555 | 0.031284 | 30 |
| BP | modulation of chemical synaptic transmission | 0.002555 | 0.031284 | 67 |
| BP | regulation of trans-synaptic signaling | 0.002555 | 0.031284 | 67 |
| BP | digestion | 0.002562 | 0.031301 | 22 |
| BP | ameboidal-type cell migration | 0.002609 | 0.031798 | 65 |
| BP | syncytium formation by plasma membrane fusion | 0.002658 | 0.032324 | 16 |
| BP | second-messenger-mediated signaling | 0.002681 | 0.032536 | 56 |
| BP | positive regulation of muscle cell differentiation | 0.002687 | 0.032544 | 20 |
| BP | acid secretion | 0.002735 | 0.033054 | 24 |
| BP | purine nucleoside triphosphate metabolic process | 0.002764 | 0.033333 | 47 |
| BP | ammonium ion metabolic process | 0.002812 | 0.033839 | 32 |
| BP | positive regulation of ERBB signaling pathway | 0.002864 | 0.034249 | 10 |
| BP | L-alpha-amino acid transmembrane transport | 0.002864 | 0.034249 | 10 |
| BP | negative regulation of peptide secretion | 0.002871 | 0.034249 | 29 |
| BP | positive regulation of cytokine secretion | 0.002871 | 0.034249 | 28 |
| BP | regulation of vasculature development | 0.002886 | 0.03435 | 56 |
| BP | neurological system process involved in regulation of systemic arterial blood pressure | 0.002919 | 0.034378 | 8 |
| BP | hexose catabolic process | 0.002919 | 0.034378 | 8 |
| BP | triglyceride catabolic process | 0.002919 | 0.034378 | 8 |
| BP | response to forskolin | 0.002919 | 0.034378 | 8 |
| BP | cellular response to forskolin | 0.002919 | 0.034378 | 8 |
| BP | neuromuscular junction development | 0.00295 | 0.034603 | 14 |
| BP | regulation of lipid catabolic process | 0.00295 | 0.034603 | 14 |
| BP | regulation of cysteine-type endopeptidase activity involved in apoptotic process | 0.002983 | 0.034916 | 39 |
| BP | regulation of glycolytic process | 0.003031 | 0.035336 | 11 |
| BP | Schwann cell differentiation | 0.003031 | 0.035336 | 11 |
| BP | positive regulation of vasoconstriction | 0.003081 | 0.035839 | 12 |
| BP | regulation of blood vessel endothelial cell migration | 0.003127 | 0.036293 | 20 |
| BP | wound healing | 0.003199 | 0.0368 | 57 |
| BP | Fc receptor signaling pathway | 0.00321 | 0.0368 | 6 |
| BP | dicarboxylic acid catabolic process | 0.00321 | 0.0368 | 6 |
| BP | fatty acid homeostasis | 0.00321 | 0.0368 | 6 |
| BP | negative regulation of lipase activity | 0.00321 | 0.0368 | 6 |
| BP | renal absorption | 0.00321 | 0.0368 | 6 |
| BP | endothelial cell proliferation | 0.003226 | 0.036908 | 28 |
| BP | regulation of steroid metabolic process | 0.003274 | 0.037383 | 21 |
| BP | cellular response to insulin stimulus | 0.003285 | 0.037438 | 35 |
| BP | regulation of cytokine biosynthetic process | 0.003392 | 0.038557 | 22 |
| BP | epithelial cell migration | 0.003397 | 0.038557 | 48 |
| BP | regulation of potassium ion transmembrane transporter activity | 0.0036 | 0.040774 | 14 |
| BP | monocarboxylic acid transport | 0.003617 | 0.040803 | 28 |
| BP | peptide hormone secretion | 0.003623 | 0.040803 | 44 |
| BP | cellular protein complex disassembly | 0.003625 | 0.040803 | 27 |
| BP | epithelium migration | 0.003676 | 0.041296 | 48 |
| BP | positive regulation of muscle cell apoptotic process | 0.003754 | 0.042004 | 10 |
| BP | negative regulation of amine transport | 0.003754 | 0.042004 | 10 |
| BP | homeostasis of number of cells | 0.003779 | 0.04212 | 53 |
| BP | regulation of striated muscle contraction | 0.003779 | 0.04212 | 18 |
| BP | xenobiotic metabolic process | 0.003798 | 0.042164 | 16 |
| BP | regulation of cardiac muscle tissue growth | 0.003798 | 0.042164 | 16 |
| BP | glycogen biosynthetic process | 0.003861 | 0.042698 | 12 |
| BP | glucan biosynthetic process | 0.003861 | 0.042698 | 12 |
| BP | regulation of generation of precursor metabolites and energy | 0.003884 | 0.04284 | 22 |
| BP | regulation of leukocyte migration | 0.00389 | 0.04284 | 36 |
| BP | positive regulation of response to external stimulus | 0.003963 | 0.04342 | 50 |
| BP | negative regulation of angiogenesis | 0.003968 | 0.04342 | 23 |
| BP | positive regulation of protein secretion | 0.003973 | 0.04342 | 48 |
| BP | tissue migration | 0.003973 | 0.04342 | 48 |
| BP | calcium ion transport into cytosol | 0.004014 | 0.043778 | 29 |
| BP | negative regulation of systemic arterial blood pressure | 0.004082 | 0.044317 | 8 |
| BP | response to platelet aggregation inhibitor | 0.004082 | 0.044317 | 8 |
| BP | drug transmembrane transport | 0.004087 | 0.044317 | 15 |
| BP | cellular carbohydrate biosynthetic process | 0.004134 | 0.044485 | 17 |
| BP | macrophage activation | 0.004134 | 0.044485 | 17 |
| BP | positive regulation of striated muscle cell differentiation | 0.004134 | 0.044485 | 17 |
| BP | regulation of heart growth | 0.004134 | 0.044485 | 17 |
| BP | glial cell differentiation | 0.004145 | 0.044513 | 40 |
| BP | regulation of peptide hormone secretion | 0.004154 | 0.044522 | 37 |
| BP | regulation of membrane potential | 0.004207 | 0.045002 | 70 |
| BP | regulation of intracellular transport | 0.004266 | 0.04555 | 71 |
| BP | regulation of synapse assembly | 0.004328 | 0.046041 | 21 |
| BP | regulation of response to drug | 0.004328 | 0.046041 | 21 |
| BP | transmission of nerve impulse | 0.004414 | 0.046859 | 18 |
| BP | urogenital system development | 0.004422 | 0.046859 | 56 |
| BP | organ growth | 0.004497 | 0.047568 | 34 |
| BP | regulation of leukocyte chemotaxis | 0.004555 | 0.048007 | 24 |
| BP | negative regulation of protein secretion | 0.004556 | 0.048007 | 27 |
| BP | response to dietary excess | 0.004606 | 0.04826 | 9 |
| BP | negative regulation of cytokine biosynthetic process | 0.004606 | 0.04826 | 9 |
| BP | response to dexamethasone | 0.004606 | 0.04826 | 9 |
| BP | serotonin transport | 0.004666 | 0.048526 | 7 |
| BP | regulation of release of sequestered calcium ion into cytosol by sarcoplasmic reticulum | 0.004666 | 0.048526 | 7 |
| BP | regulation of interleukin-2 biosynthetic process | 0.004666 | 0.048526 | 7 |
| BP | barbed-end actin filament capping | 0.004666 | 0.048526 | 7 |
| BP | fructose 6-phosphate metabolic process | 0.004749 | 0.048755 | 5 |
| BP | glucose catabolic process | 0.004749 | 0.048755 | 5 |
| BP | muscle atrophy | 0.004749 | 0.048755 | 5 |
| BP | fructose 1,6-bisphosphate metabolic process | 0.004749 | 0.048755 | 5 |
| BP | negative regulation of fatty acid oxidation | 0.004749 | 0.048755 | 5 |
| BP | regulation of cell proliferation in bone marrow | 0.004749 | 0.048755 | 5 |
| BP | regulation of amino acid transmembrane transport | 0.004749 | 0.048755 | 5 |
| BP | organic anion transport | 0.004815 | 0.049255 | 63 |
| BP | regulation of reactive oxygen species metabolic process | 0.004816 | 0.049255 | 35 |
| BP | positive regulation of ATP metabolic process | 0.004849 | 0.04944 | 10 |
| BP | peripheral nervous system development | 0.004851 | 0.04944 | 17 |
| BP | regulation of nucleotide catabolic process | 0.00489 | 0.049747 | 11 |
| CC | myofibril | 2.17E-34 | 1.43E-31 | 97 |
| CC | contractile fiber | 5.59E-33 | 1.73E-30 | 99 |
| CC | sarcomere | 7.88E-33 | 1.73E-30 | 89 |
| CC | contractile fiber part | 2.52E-31 | 4.15E-29 | 91 |
| CC | I band | 7.52E-27 | 9.90E-25 | 68 |
| CC | Z disc | 4.76E-24 | 5.22E-22 | 61 |
| CC | sarcoplasmic reticulum | 2.24E-11 | 2.05E-09 | 30 |
| CC | myofilament | 2.49E-11 | 2.05E-09 | 21 |
| CC | striated muscle thin filament | 5.48E-11 | 4.00E-09 | 20 |
| CC | sarcoplasm | 2.38E-10 | 1.56E-08 | 31 |
| CC | sarcolemma | 2.79E-09 | 1.67E-07 | 47 |
| CC | M band | 1.55E-07 | 8.04E-06 | 14 |
| CC | actin cytoskeleton | 1.59E-07 | 8.04E-06 | 99 |
| CC | A band | 2.14E-07 | 1.00E-05 | 19 |
| CC | lipid droplet | 3.06E-06 | 0.000134 | 25 |
| CC | outer membrane | 4.93E-06 | 0.000191 | 45 |
| CC | organelle outer membrane | 4.93E-06 | 0.000191 | 45 |
| CC | peroxisome | 7.67E-06 | 0.000265 | 36 |
| CC | microbody | 7.67E-06 | 0.000265 | 36 |
| CC | extracellular matrix | 1.19E-05 | 0.000392 | 85 |
| CC | mitochondrial outer membrane | 2.99E-05 | 0.000938 | 40 |
| CC | proteinaceous extracellular matrix | 3.49E-05 | 0.001043 | 70 |
| CC | neuromuscular junction | 4.28E-05 | 0.001225 | 22 |
| CC | T-tubule | 4.98E-05 | 0.001364 | 20 |
| CC | collagen trimer | 0.000164 | 0.004319 | 22 |
| CC | myelin sheath | 0.000214 | 0.005424 | 46 |
| CC | sarcoplasmic reticulum membrane | 0.000255 | 0.00622 | 10 |
| CC | myosin complex | 0.000325 | 0.007608 | 19 |
| CC | muscle myosin complex | 0.000335 | 0.007608 | 8 |
| CC | myosin II complex | 0.000388 | 0.008509 | 10 |
| CC | plasma membrane protein complex | 0.000801 | 0.017002 | 87 |
| CC | voltage-gated potassium channel complex | 0.001016 | 0.0209 | 20 |
| CC | MHC class I protein complex | 0.001093 | 0.021792 | 6 |
| CC | intercalated disc | 0.001192 | 0.023077 | 17 |
| CC | Cul3-RING ubiquitin ligase complex | 0.001378 | 0.025898 | 18 |
| CC | rough endoplasmic reticulum | 0.001528 | 0.026929 | 23 |
| CC | peroxisomal membrane | 0.001555 | 0.026929 | 13 |
| CC | microbody membrane | 0.001555 | 0.026929 | 13 |
| CC | potassium channel complex | 0.001834 | 0.030947 | 21 |
| MF | structural constituent of muscle | 2.77E-10 | 2.90E-07 | 18 |
| MF | actin binding | 5.21E-08 | 2.61E-05 | 89 |
| MF | lyase activity | 9.82E-08 | 2.61E-05 | 48 |
| MF | cofactor binding | 9.98E-08 | 2.61E-05 | 98 |
| MF | CoA-ligase activity | 1.73E-07 | 3.61E-05 | 13 |
| MF | acid-thiol ligase activity | 1.46E-06 | 0.000239 | 13 |
| MF | carbohydrate binding | 1.60E-06 | 0.000239 | 59 |
| MF | coenzyme binding | 2.05E-06 | 0.000267 | 62 |
| MF | actin filament binding | 2.44E-06 | 0.000268 | 45 |
| MF | hydro-lyase activity | 2.56E-06 | 0.000268 | 20 |
| MF | voltage-gated ion channel activity | 3.64E-06 | 0.000317 | 44 |
| MF | voltage-gated channel activity | 3.64E-06 | 0.000317 | 44 |
| MF | muscle alpha-actinin binding | 4.32E-06 | 0.000348 | 10 |
| MF | acylglycerol O-acyltransferase activity | 2.14E-05 | 0.0016 | 9 |
| MF | ligase activity, forming carbon-sulfur bonds | 3.33E-05 | 0.00232 | 14 |
| MF | carbon-oxygen lyase activity | 4.05E-05 | 0.002646 | 20 |
| MF | alpha-actinin binding | 7.51E-05 | 0.004622 | 13 |
| MF | fatty acid ligase activity | 8.17E-05 | 0.004749 | 9 |
| MF | structural constituent of cytoskeleton | 8.99E-05 | 0.004952 | 21 |
| MF | TAP binding | 0.000113 | 0.005891 | 7 |
| MF | actinin binding | 0.000123 | 0.006141 | 15 |
| MF | sulfur compound binding | 0.000134 | 0.006359 | 48 |
| MF | gated channel activity | 0.000141 | 0.006391 | 58 |
| MF | ligase activity | 0.000168 | 0.007309 | 39 |
| MF | ion gated channel activity | 0.000192 | 0.008033 | 57 |
| MF | beta-2-microglobulin binding | 0.00024 | 0.008811 | 7 |
| MF | structural molecule activity conferring elasticity | 0.00024 | 0.008811 | 7 |
| MF | pattern binding | 0.000244 | 0.008811 | 9 |
| MF | polysaccharide binding | 0.000244 | 0.008811 | 9 |
| MF | amide binding | 0.000259 | 0.009018 | 61 |
| MF | carboxylic ester hydrolase activity | 0.000275 | 0.00929 | 31 |
| MF | chemokine activity | 0.000354 | 0.011555 | 14 |
| MF | carboxy-lyase activity | 0.000481 | 0.015242 | 12 |
| MF | adrenergic receptor activity | 0.000569 | 0.017003 | 6 |
| MF | acylglycerol lipase activity | 0.000569 | 0.017003 | 6 |
| MF | scavenger receptor activity | 0.000614 | 0.017844 | 14 |
| MF | lipase activity | 0.000673 | 0.019013 | 26 |
| MF | organic acid binding | 0.000882 | 0.024267 | 41 |
| MF | NADP binding | 0.000972 | 0.026062 | 13 |
| MF | channel activity | 0.001048 | 0.026595 | 72 |
| MF | passive transmembrane transporter activity | 0.001048 | 0.026595 | 72 |
| MF | antioxidant activity | 0.001068 | 0.026595 | 20 |
| MF | oxidoreductase activity, acting on CH-OH group of donors | 0.001173 | 0.027947 | 29 |
| MF | fatty acid binding | 0.001176 | 0.027947 | 12 |
| MF | peptide binding | 0.001202 | 0.027947 | 53 |
| MF | AMP binding | 0.001378 | 0.031323 | 7 |
| MF | voltage-gated cation channel activity | 0.001516 | 0.03304 | 29 |
| MF | monocarboxylic acid binding | 0.001516 | 0.03304 | 17 |
| MF | calmodulin binding | 0.001549 | 0.033065 | 37 |
| MF | oxidoreductase activity, acting on the CH-OH group of donors, NAD or NADP as acceptor | 0.001598 | 0.033438 | 26 |
| MF | cargo receptor activity | 0.001783 | 0.036573 | 20 |
| MF | carboxylic acid binding | 0.001852 | 0.037248 | 39 |
| MF | intramolecular oxidoreductase activity, transposing C=C bonds | 0.001993 | 0.039324 | 6 |
| MF | cation channel activity | 0.002138 | 0.041414 | 53 |
| MF | T cell receptor binding | 0.002181 | 0.041475 | 7 |
| MF | fibronectin binding | 0.002243 | 0.041896 | 10 |
| MF | substrate-specific channel activity | 0.002393 | 0.043908 | 67 |
| MF | flavin adenine dinucleotide binding | 0.002458 | 0.044325 | 20 |
| MF | carbon-carbon lyase activity | 0.002529 | 0.0448 | 14 |
| MF | intramolecular oxidoreductase activity | 0.00257 | 0.0448 | 13 |

**Supplementary table 3** All KEGG pathways of downregulated mRNAs

| Description | pvalue | padj | Count |
| --- | --- | --- | --- |
| PPAR signaling pathway | 3.77E-12 | 1.13E-09 | 26 |
| Valine, leucine and isoleucine degradation | 6.55E-10 | 8.25E-08 | 20 |
| Propanoate metabolism | 8.25E-10 | 8.25E-08 | 15 |
| Regulation of lipolysis in adipocytes | 3.34E-09 | 2.51E-07 | 19 |
| Carbon metabolism | 1.04E-08 | 6.26E-07 | 28 |
| Fatty acid metabolism | 2.35E-08 | 1.17E-06 | 18 |
| Biosynthesis of amino acids | 9.52E-07 | 4.08E-05 | 19 |
| Peroxisome | 2.43E-06 | 8.41E-05 | 20 |
| Glycine, serine and threonine metabolism | 2.52E-06 | 8.41E-05 | 13 |
| AMPK signaling pathway | 1.04E-05 | 0.000312 | 24 |
| Biosynthesis of unsaturated fatty acids | 1.67E-05 | 0.000436 | 10 |
| Butanoate metabolism | 1.75E-05 | 0.000436 | 9 |
| Tryptophan metabolism | 3.72E-05 | 0.000858 | 12 |
| Fatty acid degradation | 6.26E-05 | 0.001342 | 12 |
| Drug metabolism - cytochrome P450 | 8.76E-05 | 0.001753 | 13 |
| Alanine, aspartate and glutamate metabolism | 0.000159 | 0.002982 | 10 |
| Fatty acid elongation | 0.000378 | 0.006672 | 9 |
| Insulin signaling pathway | 0.000501 | 0.008348 | 22 |
| Glycerolipid metabolism | 0.000643 | 0.010148 | 12 |
| Glyoxylate and dicarboxylate metabolism | 0.000689 | 0.010331 | 8 |
| Fat digestion and absorption | 0.0009 | 0.012863 | 8 |
| Pyruvate metabolism | 0.001004 | 0.013689 | 9 |
| Tyrosine metabolism | 0.001541 | 0.020095 | 9 |
| Pantothenate and CoA biosynthesis | 0.001667 | 0.020711 | 6 |
| Renin secretion | 0.001726 | 0.020711 | 13 |
| beta-Alanine metabolism | 0.001862 | 0.020796 | 8 |
| Glucagon signaling pathway | 0.001936 | 0.020796 | 16 |
| Glycolysis / Gluconeogenesis | 0.001993 | 0.020796 | 12 |
| Fatty acid biosynthesis | 0.00201 | 0.020796 | 5 |
| Insulin resistance | 0.002473 | 0.024728 | 17 |

**Supplementary table 4** All KEGG pathways of upregulated mRNAs

| Description | pvalue | padj | Count |
| --- | --- | --- | --- |
| Hypertrophic cardiomyopathy (HCM) | 4.05E-07 | 0.000116 | 21 |
| Tuberculosis | 1.24E-06 | 0.000178 | 31 |
| Adrenergic signaling in cardiomyocytes | 2.13E-05 | 0.002027 | 26 |
| Osteoclast differentiation | 3.57E-05 | 0.002506 | 23 |
| Phagosome | 4.61E-05 | 0.002506 | 27 |
| C-type lectin receptor signaling pathway | 5.67E-05 | 0.002506 | 21 |
| Dilated cardiomyopathy (DCM) | 6.41E-05 | 0.002506 | 18 |
| Leishmaniasis | 7.01E-05 | 0.002506 | 15 |
| Oxytocin signaling pathway | 8.57E-05 | 0.002722 | 25 |
| Starch and sucrose metabolism | 9.91E-05 | 0.002835 | 9 |
| Staphylococcus aureus infection | 0.000165 | 0.004282 | 12 |
| Proteasome | 0.00038 | 0.009055 | 11 |
| Fructose and mannose metabolism | 0.000543 | 0.011324 | 9 |
| Glucagon signaling pathway | 0.000554 | 0.011324 | 17 |
| Pentose and glucuronate interconversions | 0.000602 | 0.011481 | 7 |
| Toll-like receptor signaling pathway | 0.000753 | 0.012505 | 16 |
| IL-17 signaling pathway | 0.000753 | 0.012505 | 16 |
| Galactose metabolism | 0.000787 | 0.012505 | 8 |
| Pertussis | 0.000922 | 0.013885 | 14 |
| Cardiac muscle contraction | 0.001217 | 0.017402 | 14 |
| Cytokine-cytokine receptor interaction | 0.00148 | 0.019479 | 33 |
| Cellular senescence | 0.001498 | 0.019479 | 24 |
| Salmonella infection | 0.001587 | 0.019728 | 14 |
| Glycolysis / Gluconeogenesis | 0.001676 | 0.019978 | 12 |
| Rheumatoid arthritis | 0.001804 | 0.020635 | 14 |
| FoxO signaling pathway | 0.002372 | 0.026092 | 19 |
| Arginine and proline metabolism | 0.002576 | 0.026874 | 10 |
| AGE-RAGE signaling pathway in diabetic complications | 0.002724 | 0.026874 | 16 |
| NOD-like receptor signaling pathway | 0.002725 | 0.026874 | 22 |
| Calcium signaling pathway | 0.002838 | 0.027056 | 24 |
| Legionellosis | 0.003032 | 0.027974 | 11 |
| Fluid shear stress and atherosclerosis | 0.003561 | 0.031825 | 20 |
| HIF-1 signaling pathway | 0.003707 | 0.032131 | 16 |
| Glioma | 0.0062 | 0.049497 | 12 |
| Arrhythmogenic right ventricular cardiomyopathy (ARVC) | 0.0062 | 0.049497 | 12 |
| Chemokine signaling pathway | 0.00623 | 0.049497 | 24 |
